# First-line immunotherapy for advanced hepatocellular carcinoma: a network meta-analysis of randomized trials with overall and HBV/HCV-stratified efficacy and safety

**DOI:** 10.3389/fimmu.2026.1693251

**Published:** 2026-05-14

**Authors:** Wei Chen, Boyan Chen, Chunbin Hu, Qiance Wei, Lili Zhang, Wenwen Fu

**Affiliations:** 1 Department of Pharmacy, Emergency General Hospital, Beijing, China; 2 The First School of Clinical Medicine, Nanjing University of Chinese Medicine, Nanjing, China; 3 School of Basic Medical Sciences, Capital Medical University, Beijing, China; 4 Department of Clinical Nutrition, The Sixth Affiliated Hospital, Sun Yat-sen University, Guangzhou, Guangdong, China; 5 Biomedical Innovation Center, The Sixth Affiliated Hospital, Sun Yat-sen University, Guangzhou, Guangdong, China

**Keywords:** efficacy, HBV/HCV, hepatocellular carcinoma, ICIS, network meta-analysis, safety

## Abstract

**Background:**

Immune checkpoint inhibitors (ICIs) have revolutionized the front-line treatment of advanced hepatocellular carcinoma (HCC). However, the comparative efficacy and safety of different ICI-based regimens—and their consistency across etiologic subgroups [HBV, HCV, and non-HBV/non-HCV (NBNC)]—remain uncertain. This study conducted a Bayesian network meta-analysis (NMA) of recent randomized controlled trials (RCTs) to compare the first-line immunotherapy strategies both overall and by viral etiology.

**Methods:**

A systematic search of PubMed, Embase, the Cochrane Library, and Web of Science was conducted from inception through August 1, 2025. Prespecified primary outcomes were overall survival (OS) and progression-free survival (PFS); secondary outcomes included objective response rate (ORR) and grade ≥3 treatment-related adverse events (TRAEs). Hazard ratios (HRs) and odds ratios (ORs) were pooled in a Bayesian NMA contrasting ICI-based regimens with tyrosine kinase inhibitor (TKI) monotherapy in the overall advanced HCC population and in HBV, HCV, and NBNC strata. Protocol registration: PROSPERO CRD420251131167.

**Results:**

Ten RCTs (n=7,301) evaluating 14 first-line regimens were included. Compared to TKI monotherapy, immunotherapy significantly improved OS (HR = 0.79, 95% CI 0.74-0.84) and PFS (HR = 0.70, 95% CI 0.58-0.85), increased ORR (OR = 3.20, 95% CI 2.49-4.12), and did not significantly increase grade ≥3 TRAEs (OR = 1.16, 95% CI 0.75-1.80). Benefits were more pronounced in HBV-positive patients (OS: HR = 0.73, 95% CI 0.67-0.79; PFS: HR = 0.54, 95% CI 0.48-0.61). HCV-positive patients also derived an OS benefit (HR = 0.82, 95% CI 0.72-0.93), whereas PFS improvement in NBNC patients was not statistically significant (HR = 0.74, 95% CI 0.52-1.06). In the NMA, sintilimab plus a bevacizumab biosimilar (Sinti-Bev) showed the greatest OS improvement versus sorafenib (HR = 0.57, 95% CI 0.43-0.75), followed by camrelizumab plus rivoceranib (Camre-Rivo; HR = 0.62, 95% CI 0.49-0.80). For PFS, anlotinib plus penpulimab (Anlo-Penpu) and Camre-Rivo ranked the highest (both HR = 0.52, 95% CI 0.41-0.66). Tislelizumab and nivolumab were associated with lower risks of grade ≥3 TRAEs. In etiology-stratified network analyses, atezolizumab plus bevacizumab (Atezo-Bev) showed the most consistent efficacy profile across virally mediated subgroups, with significant advantages for both OS and PFS in HBV-positive disease and a significant OS advantage in HCV-positive disease. However, no regimen achieved a statistically significant PFS advantage in the HCV-positive subgroup.

**Conclusions:**

First-line immunotherapy, compared to TKI monotherapy, provides meaningful survival benefits in advanced HCC, particularly in virally mediated disease, without significantly increasing severe toxicity. Among regimens, Sinti-Bev offers the most substantial OS advantage, while Anlo-Penpu and Camre-Rivo rank highest for PFS. Etiology-stratified analyses highlight Atezo-Bev as the most consistent regimen in virally mediated disease, with significant OS and PFS benefits in HBV-positive patients and a significant OS benefit in HCV-positive patients.

**Systematic Review Registration:**

https://www.crd.york.ac.uk/prospero/display_record.php?ID=CRD420251131167, identifier CRD420251131167.

## 1 Introduction

According to the most recent GLOBOCAN report, liver cancer is the sixth most common malignancy worldwide and the third leading cause of cancer death, with HCC comprising 75-85% of primary liver cancers (1). Despite broadened surveillance efforts, roughly 30-40% of patients are diagnosed at an unresectable or otherwise advanced stage (2). Historically, first-line therapy has centered on TKIs, which can delay progression but yield only modest improvements in OS and exhibit substantial heterogeneity in toxicity, underscoring the need for more effective, stratifiable treatment options (3).

ICIs have expanded systemic options for advanced HCC. By inhibiting pathways such as PD-1/PD-L1 or CTLA-4, ICIs restore tumor-specific T-cell activity. However, the coexistence of malignancy with chronic liver disease—marked by intrinsic hepatic immune tolerance, VEGF-driven vascular aberrations, and accumulation of myeloid-derived suppressor cells (MDSCs)—creates an immunosuppressive milieu that may blunt responses to ICI monotherapy (4). In line with the vascular-normalization hypothesis, appropriately dosed and timed anti-VEGF therapy can improve perfusion and permeability, reduce interstitial pressure, upregulate adhesion molecules, and facilitate effector T-cell infiltration, providing a biological rationale for ICI-based combinations (5).

In recent years, several landmark first-line RCTs have reshaped the therapeutic landscape. IMbrave150 showed that atezolizumab plus bevacizumab significantly prolonged OS and improved PFS and ORR versus sorafenib; HIMALAYA demonstrated an OS advantage with the STRIDE regimen (tremelimumab priming followed by durvalumab maintenance) over sorafenib; and ORIENT-32, largely enrolling a Chinese cohort, confirmed significant OS and PFS benefits with sintilimab plus a bevacizumab biosimilar (6–8). By contrast, COSMIC-312 (atezolizumab plus cabozantinib) improved PFS without an OS benefit, and LEAP-002 (lenvatinib plus pembrolizumab) did not achieve its OS superiority target (9, 10). Most recently, the 2025 APOLLO trial reported that anlotinib plus penpulimab improved both OS and PFS versus sorafenib, further broadening first-line options (11).

At the population level, HCC is etiologically heterogeneous (HBV, HCV, and non-HBV/non-HCV), and the relative benefits of immunotherapy-based combinations across these subgroups remain debated. Emerging evidence suggests that patients with HBV- or HCV-related disease may experience more consistent survival gains, heightening the need for etiology-stratified, evidence-based recommendations (12). Recent years have also seen the emergence of network meta-analyses comparing first-line systemic therapies for advanced HCC. Among them, Li et al. conducted a comprehensive NMA of first-line regimens, including both targeted agents and immune checkpoint inhibitor-based combinations, and explored subgroup effects by geographic region and viral etiology (13). However, the subgroup analyses in that study were restricted to OS and did not extend to PFS. In addition, the analysis was not specifically designed as an etiology-stratified comparison of contemporary ICI-based first-line strategies across HBV, HCV, and non-viral HCC, and it did not incorporate the most recent pivotal RCTs such as APOLLO and CheckMate 9DW.

Against this backdrop, a systematic review and Bayesian network meta-analysis of contemporary first-line RCTs was undertaken. OS and PFS were prespecified as primary endpoints, with ORR and grade ≥3 TRAEs as key secondary endpoints. The analysis compares the benefit-risk profiles of ICI-based first-line strategies and quantifies net benefit across HBV, HCV, and NBNC subgroups, providing a robust evidence base to guide regimen selection across diverse clinical contexts.

## 2 Materials and methods

This network meta-analysis adhered to the PRISMA extension for NMAs (**Supplementary Table S1**) (14). Because head-to-head RCTs directly comparing ICI regimens are scarce, a Bayesian NMA was used to synthesize direct and indirect evidence and to estimate comparative effectiveness and safety of first-line immunotherapy for advanced HCC in the overall cohort and by HBV/HCV strata (15). The protocol was preregistered on PROSPERO (CRD420251131167).

### 2.1 Data sources and search strategy

PubMed, Embase, the Cochrane Library, and Web of Science were searched from database inception to August 1, 2025. Search strategies combined controlled vocabulary (MeSH/Emtree) and free-text terms related to hepatocellular carcinoma, randomized clinical/controlled trials, immune checkpoint inhibitors, and specific agents, including “hepatocellular carcinoma,” “randomized clinical trial,” “randomized controlled trial,” “immune checkpoint inhibitors,” “PD-1 inhibitor,” “PD-L1 inhibitor,” “CTLA-4 inhibitor,” “tremelimumab,” “durvalumab,” “camrelizumab,” “tislelizumab,” “pembrolizumab,” “ipilimumab,” “nivolumab,” “sintilimab,” “atezolizumab,” and “penpulimab.” Complete, database-specific strings are provided in **Supplementary Table S2**.

### 2.2 Selection criteria

#### 2.2.1 Inclusion criteria

1. Study design: RCTs—double-blind, single-blind, or open-label.
2. Participants: Adults with unresectable or advanced hepatocellular carcinoma (HCC) confirmed histologically or cytologically; etiologies included HBV, HCV, or non-viral disease. Trials were eligible if etiology-stratified results were available.
3. Interventions: First-line regimens containing ICIs, given as monotherapy or in combination with TKIs, anti-VEGF agents, or chemotherapy.
4. Comparators: Placebo, targeted agents (e.g., sorafenib, lenvatinib), or other standard-of-care therapies.
5. Outcomes (clearly defined and extractable):
6. OS: Time from randomization to death from any cause.; PFS: Time from randomization to first documented disease progression or death; ORR: Proportion of complete response (CR) plus partial response (PR) per RECIST v1.1 or mRECIST; Grade ≥3 treatment-related adverse events (TRAEs): incidence of treatment-related grade 3 or higher adverse events according to the Common Terminology Criteria for Adverse Events (CTCAE), as reported in the original trial publication or **Supplementary Materials**.

#### 2.2.2 Exclusion criteria

1. Duplicate reports: Multiple publications from the same trial cohort at different time points or with overlapping outcomes; when duplicates were identified, the report with the most complete data or the longest follow-up was retained.
2. Irrelevant study designs: Nonrandomized studies (e.g., observational or cohort studies, single-arm trials), case series, case reports, conference abstracts, reviews, and editorials.
3. Incomplete outcomes: Studies lacking clear reporting of OS, PFS, ORR, or Grade ≥3 TRAEs, and for which requisite data could not be recovered from **Supplementary Materials** or through author contact.

### 2.3 Data extraction and risk-of-bias assessment

Data were independently abstracted by three reviewers following PRISMA guidance; any conflicts were settled by consensus with a fourth investigator. Extracted fields comprised: trial identifier; study design characteristics (phase, blinding); allocation ratio; journal and year of publication; disease stage/eligibility criteria; trial registry number; sample size; baseline demographics (age and sex); and full details of experimental and control regimens (drug, dose, schedule).

Risk of bias was appraised independently by two reviewers using the Cochrane Risk of Bias 2.0 (RoB 2) tool across five domains: the randomization process; deviations from intended interventions; missing outcome data; outcome measurement; and selection of the reported result (16). Domain judgments were informed by the RoB 2 signalling questions (Yes/Probably yes/Probably no/No/No information) and summarized as Low risk, some concerns, or High risk, with an overall rating assigned accordingly. Disagreements were arbitrated by a third reviewer.

### 2.4 Statistical analysis

Primary endpoints were OS and PFS; secondary endpoints included ORR and grade ≥3 TRAEs. For time-to-event outcomes (OS, PFS), effects were summarized as hazard ratios (HRs) with 95% confidence intervals (CIs); for binary outcomes (ORR, grade ≥3 TRAEs), odds ratios (ORs) with 95% CIs were used.

A Bayesian NMA was implemented in R using the gemtc (v4.4) and rjags packages. Given the sparse, largely star-shaped treatment network and the fact that most treatment contrasts were informed by a single study, a fixed-effect model was prespecified as the primary NMA approach, because between-study heterogeneity could not be estimated reliably for most contrasts in a random-effects framework (17). A random-effects model was additionally fitted in sensitivity analyses to assess the robustness of the findings under a more conservative modeling assumption (18). Posterior distributions were estimated via Markov chain Monte Carlo (MCMC) using four parallel chains with 25,000 burn-in iterations (thinning interval = 1), followed by 120,000 sampling iterations per chain. Convergence was evaluated visually using trace and density plots and formally with the Gelman-Rubin diagnostic (potential scale reduction factor, PSRF). Posterior ranking probabilities were derived using rank.probability(), and a ranking probability heatmap was generated with pheatmap to visualize comparative ordering across regimens. Consistency was examined by comparing deviance information criterion (DIC) values between consistency and inconsistency models; a DIC difference <5 was interpreted as indicating no meaningful inconsistency. Effect estimates, treatment rankings, and model fit were compared between the primary fixed-effect model and the random-effects sensitivity model.

To reinforce robustness, conventional pairwise meta-analyses of head-to-head comparisons were also conducted in RevMan 5.4. Fixed-effect models were applied when heterogeneity was low (*I²* ≤50% or Cochran’s Q *P* ≥0.10); otherwise, random-effects models were used (*I²* >50% or *P* < 0.10). To explore potential confounding by regional differences, we pre-specified a threshold of ≥ 60% Asian enrolment to define Asia-enriched trials. This threshold was selected to ensure that the Asia-enriched subgroup consisted of trials with a sufficient proportion of Asian participants, while allowing for meaningful comparisons with non-Asia-enriched trials. For results with notable heterogeneity, leave-one-out sensitivity analyses assessed the influence of individual studies on pooled effects. Publication bias was explored by visual inspection of funnel plots. All statistical tests were two-sided with α=0.05.

## 3 Results

### 3.1 Characteristics of included studies and study selection process

**Figure 1:**
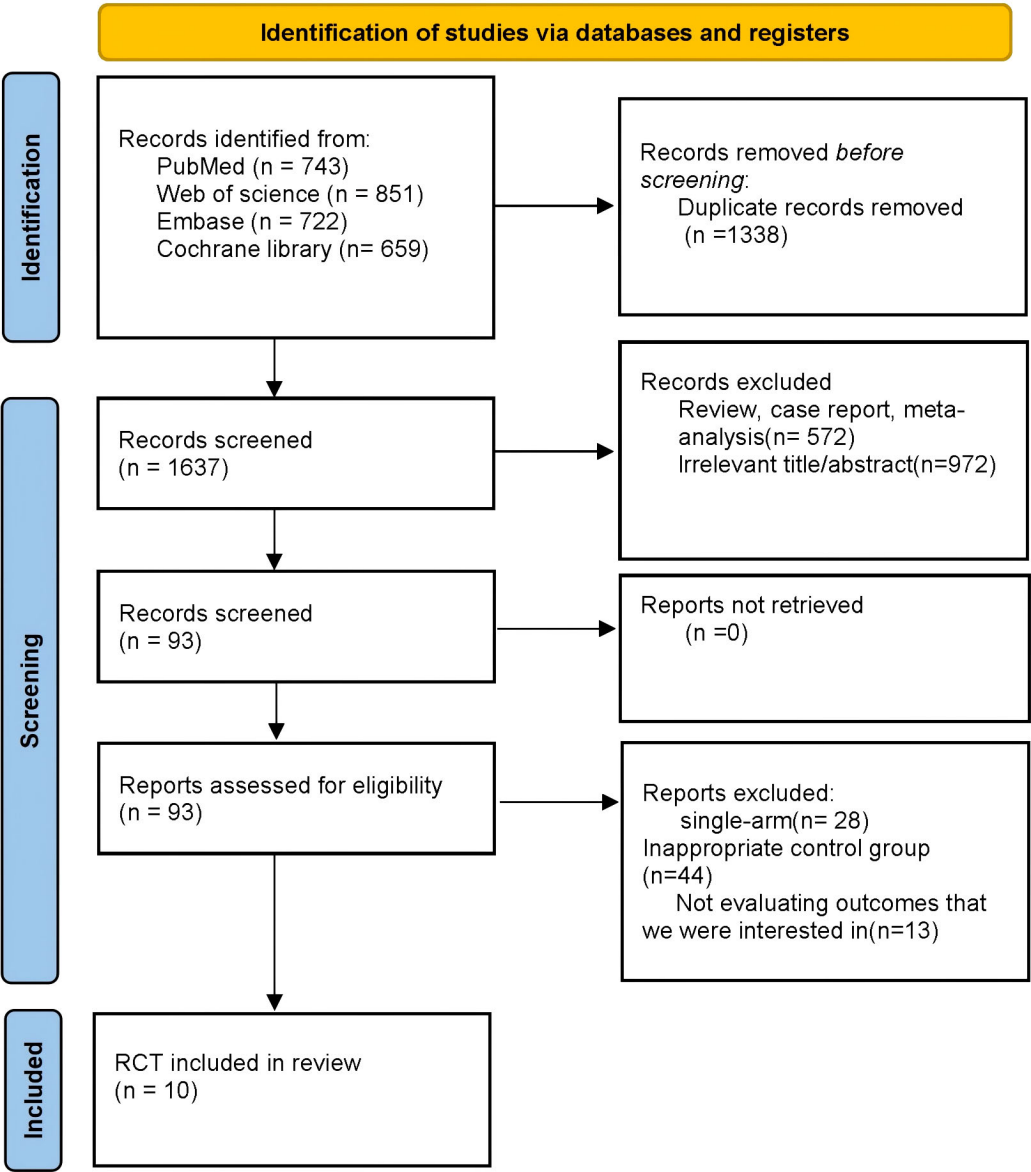
PRISMA 2020 flow diagram of study selection.

A total of 2,975 records were retrieved from four databases—PubMed (n=743), Web of Science (n=851), Embase (n=722), and the Cochrane Library (n=659). After removal of duplicates (n=1,338), 1,637 records were screened by title and abstract, of which 1,544 were excluded. Ninety-three articles underwent full-text assessment; 83 were excluded, and 10 RCTs met the eligibility criteria and were included (**Figure 1**). In aggregate, these 10 RCTs enrolled 7,301 patients with advanced HCC.

Across trials, 14 distinct first-line regimens were evaluated: tremelimumab plus durvalumab (Treme-Durva); durvalumab (Durva); camrelizumab plus rivoceranib (Camre-Rivo); tislelizumab (Tisle); lenvatinib plus pembrolizumab (Lenva-Pem); cabozantinib plus atezolizumab (Cabo-Atezo); cabozantinib (Cabo); anlotinib plus penpulimab (Anlo-Penpu); nivolumab plus ipilimumab (Nivo-Ipi); sintilimab plus bevacizumab (Sinti-Bev); atezolizumab plus bevacizumab (Atezo-Bev); nivolumab (Nivo); lenvatinib (Lenva); and sorafenib (Soraf).

Key study characteristics—study identifier, publication source, registry ID, sample size, line of therapy, phase and design (including randomization scheme), median age, sex distribution, ethnicity, intervention arm(s), and control arm—are summarized in **Table 1**.

**Table 1:** Baseline characteristics of the randomized trials included in the NMA.

Study	Source	Registered ID	Sample size	Line(s) of therapy	Race/ethnicity (%)	Intervention arm(s)	Control arm
Phase, design	(y)	Randomization	Median age/y	Male/female(n)			
HIMALAYA (7)	Ann Oncol	NCT03298451	393/389/389	First-line therapy	Asia(excluding Japan):94(38.2%) Rest of the world:152(61.8%)	Arm1:300 mg of tremelimumab (one dose) plus 1500 mg of durvalumab every 4 weeks	400 mg of sorafenib twice daily
open-label, III	2024	1:1:1	65/64/64	987/184		Arm2:1500 mg of durvalumab monotherapy every 4 weeks	
CARES-310 (19)	Lancet	NCT03764293	272/271	First-line therapy	Asian:450(82.9%) White:90(16.6%) Black or African American:1(0.2%) Other:2(0.4%)	200 mg of camrelizumab intravenously every 2 weeks plus 250 mg of rivoceranib orally once daily	400 mg of sorafenib twice daily
open-label, III	2023	1:1	58/56	455/86			
RATIONALE-301 (20)	JAMA Oncol	NCT03412773	342/332	First-line therapy	Asia (excluding Japan):425(63.1%) Japan:77(11.4%) Europe, the UK, and the US:172(25.5%)	200 mg of tislelizumab intravenously every 3 weeks	400 mg of sorafenib twice daily
open-label, III	2023	1:1	62/60	570/104			
LEAP-002 (10)	Lancet Oncol	NCT03713593	395/399	First-line therapy	Asian:345(43.5%) Black or African American:13(1.6%) White:345(43.5%) American Indian or Alaska native:21(2.6%)	8 mg (for bodyweight <60 kg) or 12 mg (for bodyweight ≥60 kg) administered orally once daily plus 200 mg of pembrolizumab intravenously every 3 weeks	8 mg (for bodyweight <60 kg) or 12 mg (for bodyweight ≥60 kg) administered orally once daily plus placebo intravenously every 3 weeks
double-blind,III	2023	1:1	66/66	644/150			
APOLLO (11)	Lancet Oncol	NCT04344158	433/216	First-line therapy	Chinese:649(100%)	10 mg of anlotinib orally once daily on days 1–14 plus 200 mg of penpulimab intravenously on day 1 of a 21-day cycle	400 mg of sorafenib orally twice daily in a 21-day cycle
open-label, III	2025	2:1	57/56	551/98			
CheckMate 9DW (21)	Lancet	NCT04039607	335/333	First-line therapy	White:353(52.8%) Asian:292(43.71%) Black:15(2.2%) other:8(1.1%)	nivolumab 1 mg/kg plus ipilimumab 3 mg/kg intravenously every 3 weeks for up to four doses, followed by 480 mg of nivolumab monotherapy intravenously every 4 weeks	8 mg of lenvatinib orally once daily (for bodyweight <60 kg) or 12 mg orally once daily (for bodyweight ≥60 kg), or 400 mg of sorafenib orally twice daily
open-label, III	2025	1:1	65/66	548/120			
ORIENT-32 (8)	Lancet Oncol	NCT03794440	380/191	First-line therapy	Chinese:571(100%)	200 mg of sintilimab intravenously over 60 minutes followed by 15 mg/kg of IBI305 (bevacizumab biosimilar) intravenously over 90 minutes (second infusion over 60 minutes, and subsequent infusions over 30 minutes if no infusion reaction occurred) every 3 weeks	400 mg of sorafenib twice daily
open-label, II/III	2021	2:1	53/54	505/66			
IMbrave150 (6)	J Hepatol	NCT03434379	336/165	First-line therapy	Asia(excluding Japan):201(40.1%) United States, Australia, New Zealand, and Japan:300(59.9%)	1,200 mg of atezolizumab plus 15 mg/ kg of bevacizumab intravenously every 3 weeks	400 mg of sorafenib twice daily
open-label, III	2021	2:1	64/66	414/87			
CheckMate 459 (22)	Lancet Oncol	NCT02576509	371/372	First-line therapy	White:395(53.2%) Asian:332(44.7%) black:5(0.67%) other:11(1.5%)	nivolumab 240 mg intravenously every 2 weeks	400 mg of sorafenib twice daily
open-label, III	2022	1:1	65/65	631/112			
COSMIC-312 (9)	Lancet Oncol	NCT03755791	432/188/217	First-line therapy	Asian:263(31.4%) Black:14(1.7%) White:426(50.9%) Other:25(3.4%)	Arm1: 40 mg of cabozantinib orally once daily plus 1200 mg of atezolizumab intravenously every 3 weeks	400 mg of sorafenib twice daily
open-label, III	2024	2:1:1	64/64/64	704/133		Arm2: 60 mg of cabozantinib orally once daily	

### 3.2 Risk of bias assessment

**Figure 2:**
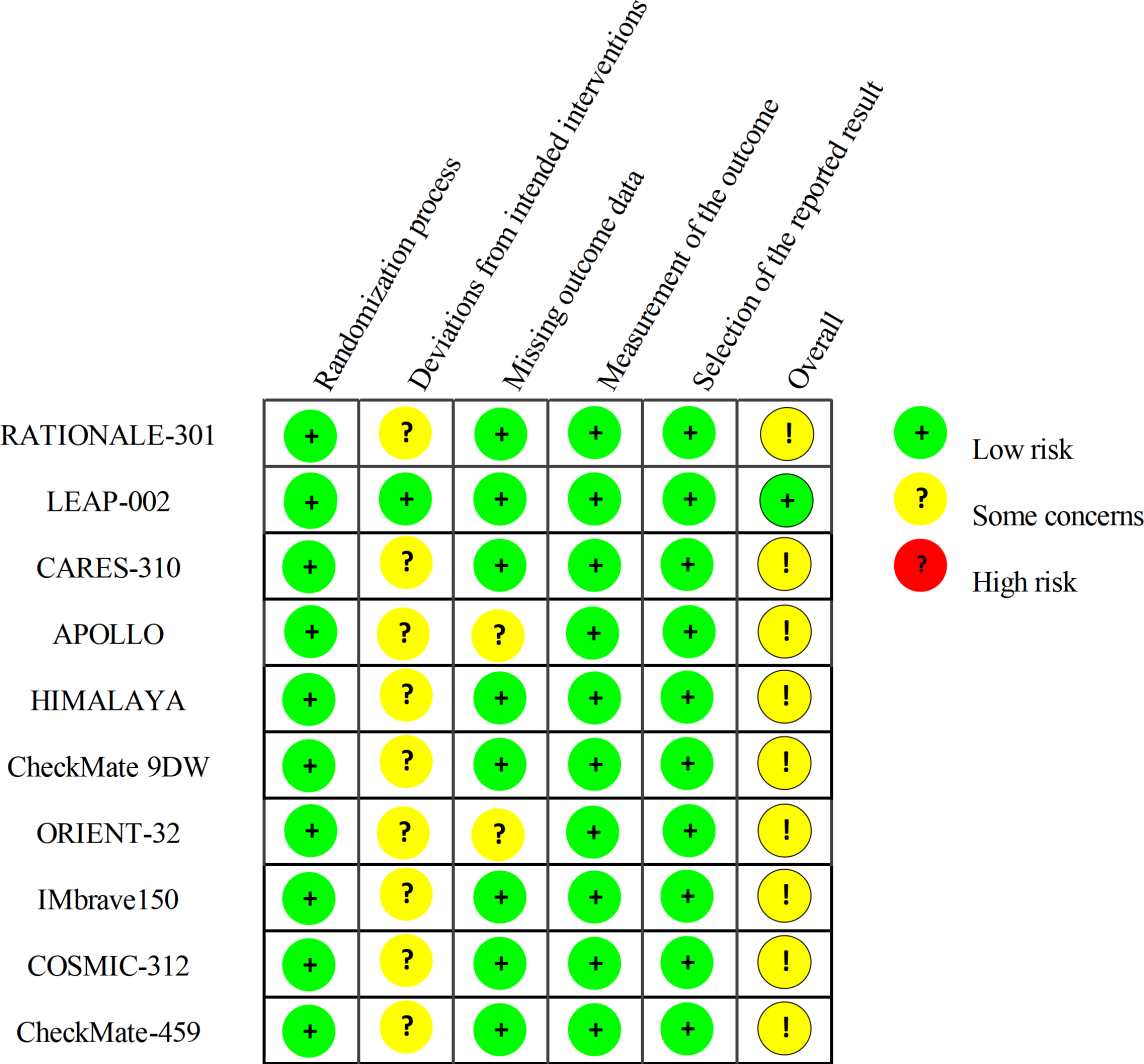
Risk-of-bias assessment using the Cochrane RoB 2 tool.

Risk of bias was assessed with the Cochrane RoB 2 tool. Across the ten randomized trials, risk was generally low for the randomization process (Domain 1), measurement of outcomes (Domain 4), and selection of the reported result (Domain 5). For missing outcome data (Domain 3), APOLLO and ORIENT-32 were judged as having some concerns, primarily owing to interim analyses, differential follow-up, or insufficiently detailed reporting of attrition. Given the predominance of open-label designs (9/10 trials), deviations from intended interventions (Domain 2) were commonly rated as some concerns, and most trials received an overall RoB judgment of some concerns. LEAP-002—the only double-blind study—was rated overall as low risk. **Figure 2** summarizes the RoB 2 assessments.

Importantly, although open-label designs can affect Domain 2, the principal endpoint in most trials was the objective outcome of overall survival, and radiographic endpoints were typically evaluated by blinded independent review committees. These features mitigate the likelihood that lack of blinding introduced material bias into the conclusions.

### 3.3 Pairwise meta-analysis

#### 3.3.1 Overall OS, PFS, ORR and grade ≥3 TRAEs

**Figure 3:**
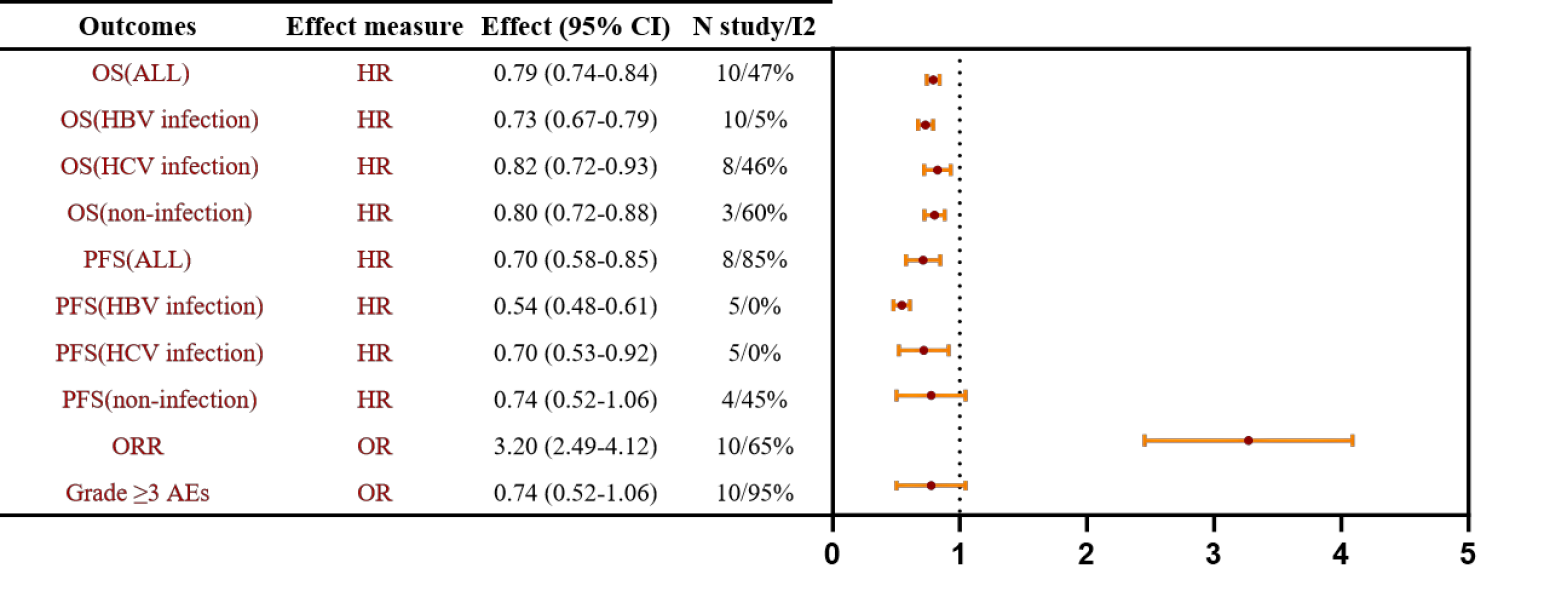
Forest plot of pooled effect sizes for first-line ICI-based therapy versus TKIs in advanced HCC, overall and stratified by HBV/HCV infection status.

All ten trials reported OS. Between-study heterogeneity was acceptable (*I²* = 47%, *P*>0.10); therefore, a fixed-effect model was used. The pooled estimate showed that immunotherapy significantly prolonged OS versus TKI monotherapy (HR = 0.79, 95% CI 0.74-0.84). In etiology-stratified analyses, the greatest benefit was observed in HBV-positive patients (HR = 0.73, 95% CI 0.67-0.79), with a significant advantage also in HCV-positive patients (HR = 0.82, 95% CI 0.72-0.93). A significant improvement was likewise seen in the NBNC subgroup (HR = 0.80, 95% CI 0.72-0.88). The corresponding forest plot is presented in **Figure 3**; **Supplementary Figure S1**.

Eight trials reported PFS. Given substantial between-study heterogeneity (*I²* = 85%, *P* < 0.10), a random-effects model was used. The pooled estimate showed that immunotherapy significantly prolonged PFS versus TKI monotherapy (HR = 0.70, 95% CI 0.58-0.85). In etiology-stratified analyses, HBV-positive patients derived the greatest benefit (HR = 0.54, 95% CI 0.48-0.61), with a significant advantage also observed in HCV-positive patients (HR = 0.70, 95% CI 0.53-0.92). In contrast, the PFS improvement in the NBNC subgroup did not reach statistical significance (HR = 0.74, 95% CI 0.52-1.06). The corresponding forest plot is presented in **Figure 3**; **Supplementary Figure S2**.

Ten trials reported ORR. Given moderate-to-high between-study heterogeneity (*I²* = 65%, *P* < 0.10), a random-effects model was applied. The pooled estimate showed that immunotherapy significantly increased ORR relative to TKI monotherapy (OR = 3.20, 95% CI 2.49-4.12). The corresponding forest plot is presented in **Figure 3**; **Supplementary Figure S3**.

Ten trials reported grade ≥3 TRAEs. Owing to substantial between-study heterogeneity (*I²* = 95%, *P* < 0.10), a random-effects model was used. The pooled estimate indicated no statistically significant increase in the risk of grade ≥3 TRAEs with immunotherapy versus TKI monotherapy (OR = 1.16, 95% CI 0.75-1.80). The corresponding forest plot is shown in **Figure 3**; **Supplementary Figure S4**.

#### 3.3.2 Definition of Asia-enriched versus non-Asia-enriched trials

Using a prespecified threshold of ≥60% Asian enrolment to define Asia-enriched trials, three RCTs were classified as Asia-enriched and seven as non-Asia-enriched.

Among patients with HBV-related HCC, subgroup analyses by geographic composition showed that first-line ICI-based regimens improved OS compared with TKIs irrespective of whether trials were Asia-enriched (**Supplementary Figure S5**). In Asia-enriched trials, ICI-based therapy was associated with a significant reduction in the risk of death (HR 0.77, 95% CI 0.67-0.88; I²=49%). A comparable benefit was observed in non-Asia-enriched trials (HR 0.71, 95% CI 0.63-0.79; I²=0%). The test for subgroup differences was not significant (P = 0.38), indicating no evidence that the magnitude of OS benefit with ICI-based regimens in HBV-positive patients differed between Asia-enriched and non-Asia-enriched trials.

In patients with HCV-related HCC, first-line ICI-based regimens also tended to improve OS relative to TKIs in both Asia-enriched and non-Asia-enriched trials (**Supplementary Figure S6**). In Asia-enriched trials, ICI-based therapy significantly prolonged OS (HR 0.59, 95% CI 0.37-0.93; I²=0%). In non-Asia-enriched trials, the pooled HR was 0.82 (95% CI 0.67-1.00; I²=51%), suggesting a borderline significant survival advantage. The interaction test again showed no significant subgroup difference (P = 0.19), implying that the OS benefit of ICI-based regimens over TKIs in HCV-related HCC was broadly comparable between Asia-enriched and non-Asia-enriched studies.

When PFS was evaluated in HBV-related HCC, first-line ICI-based regimens conferred consistent and statistically significant benefits over TKIs across geographic strata (**Supplementary Figure S7**). In Asia-enriched trials (APOLLO and CARES-310), the pooled HR for PFS was 0.55 (95% CI 0.46-0.65; I²=0%), indicating a marked delay in disease progression. In non-Asia-enriched trials, the pooled HR was 0.54 (95% CI 0.46-0.63; I²=0%), closely mirroring the benefit seen in Asia-enriched studies. The test for subgroup differences was non-significant (P = 0.91), suggesting that, in HBV-positive patients, the PFS advantage of ICI-based regimens over TKIs did not depend on whether a trial predominantly enrolled Asian or non-Asian participants.

For HCV-related HCC, only CARES-310 contributed PFS data in the Asia-enriched stratum (HR = 0.46, 95% CI 0.21-1.03), indicating a numerically favourable but statistically non-significant trend towards prolonged PFS with ICI-based treatment (**Supplementary Figure S8**). In non-Asia-enriched trials (COSMIC-312 and IMbrave150), ICI-based regimens were associated with a borderline significant PFS benefit over TKIs (HR = 0.74, 95% CI 0.55-1.00; I²=0%). The overall test for subgroup differences was not significant (P = 0.28), indicating no clear evidence that the PFS effect of ICI-based therapy versus TKIs in HCV-related HCC differed between Asia-enriched and non-Asia-enriched trials.

### 3.4 Network meta-analyses

#### 3.4.1 Comparisons of OS,PFS, ORR and grade ≥3 TRAEs

**Figure 4:**
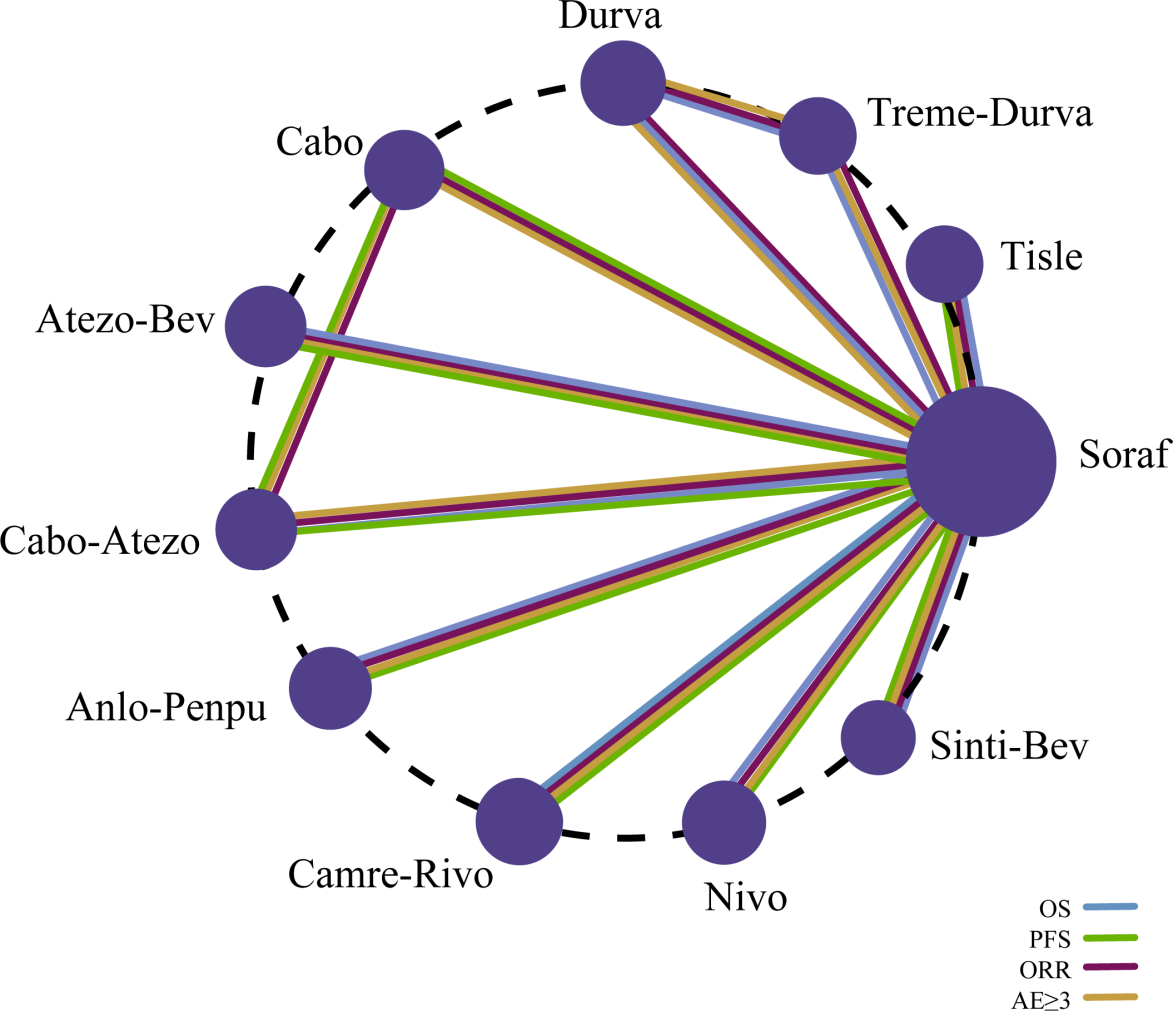
Evidence network comparing ICI-based regimens in advanced HCC across efficacy (OS, PFS, ORR) and safety (grade ≥3 TRAEs) endpoints.

Primary endpoints were OS and PFS; secondary endpoints were ORR and grade ≥3 adverse events. In the NMA, 10 regimens contributed evidence for OS and 9 for PFS, whereas 11 regimens informed the analyses of ORR and grade ≥3 adverse events. The network geometry is presented in **Figure 4**.

**Figure 5:**
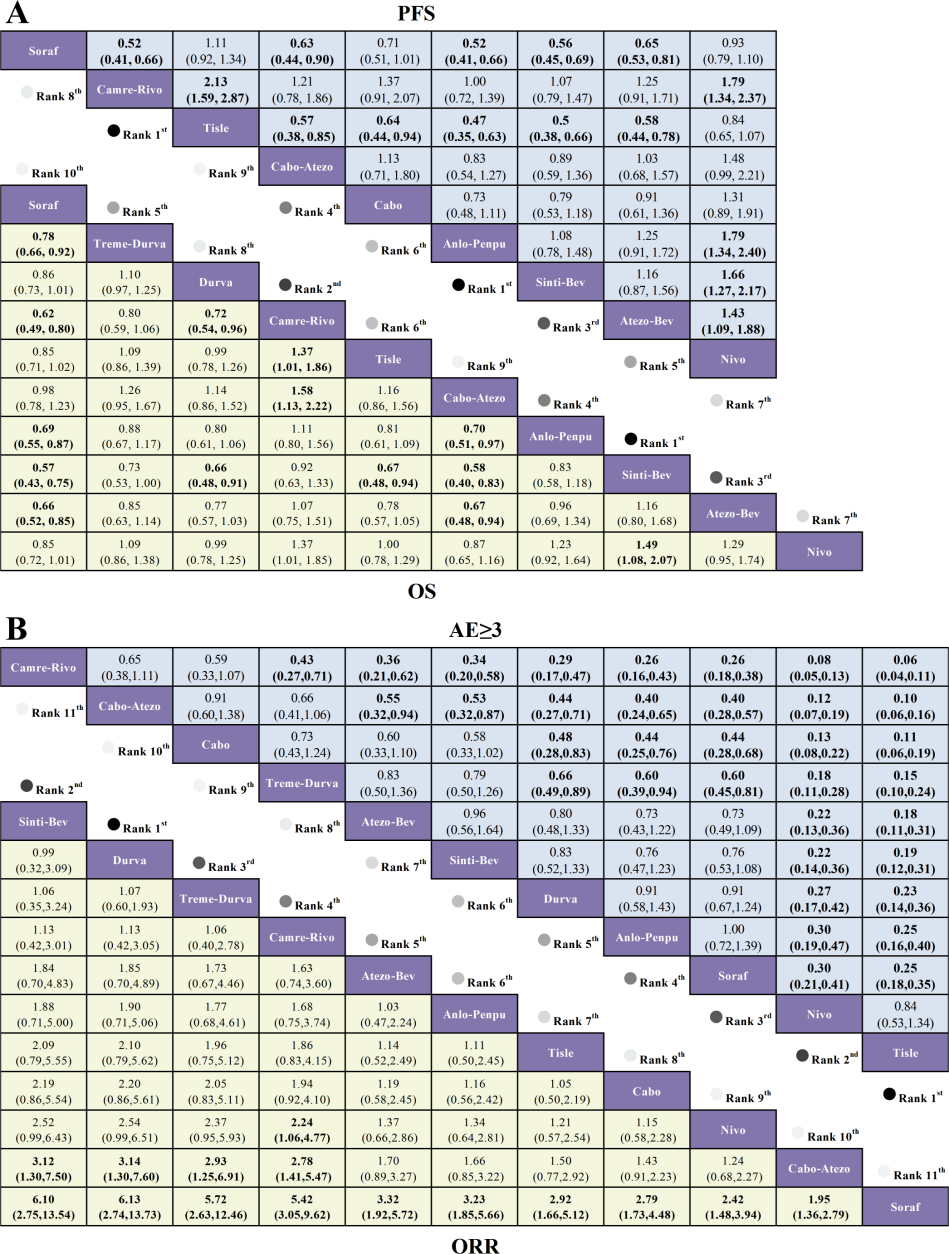
League tables from a Bayesian network meta-analysis comparing first-line immunotherapy regimens in advanced hepatocellular carcinoma. **(A)** Lower triangle (yellow): OS—HRs with 95% CIs; Upper triangle (blue): PFS—HRs with 95% CIs. For both OS and PFS, HR < 1.00 favors the row regimen (greater survival benefit). **(B)** Lower triangle (yellow): ORR—ORs with 95% CIs (OR > 1.00 favors the row regimen); Upper triangle (blue): grade ≥3 TRAEs—ORs with 95% CIs (OR < 1.00 indicates fewer severe AEs and thus better safety for the row regimen).

In terms of OS, relative to sorafenib, sintilimab plus a bevacizumab biosimilar (Sinti-Bev) conferred the greatest benefit (HR = 0.57, 95% CI 0.43-0.75), followed by camrelizumab plus rivoceranib (Camre-Rivo; HR = 0.62, 95% CI 0.49-0.80) and atezolizumab plus bevacizumab (HR = 0.66, 95% CI 0.52-0.85), whereas cabozantinib plus atezolizumab did not differ significantly (HR = 0.98, 95% CI 0.78-1.23). For PFS, anlotinib plus penpulimab (Anlo-Penpu) and Camre-Rivo achieved similarly pronounced benefits (both HR = 0.52, 95% CI 0.41-0.66), with Sinti-Bev next (HR = 0.56, 95% CI 0.45-0.69). For ORR, all immunotherapy regimens outperformed sorafenib; the largest gains were seen with Sinti-Bev (OR = 6.10, 95% CI 2.75-13.54), durvalumab monotherapy (OR = 6.13, 95% CI 2.74-13.73), and tremelimumab plus durvalumab (Treme-Durva; OR = 5.72, 95% CI 2.63-12.46). For grade ≥3 adverse events, only tislelizumab (OR = 0.25, 95% CI 0.18-0.35) and nivolumab (OR = 0.30, 95% CI 0.21-0.41) were associated with significantly lower risks versus sorafenib. Network estimates for OS/PFS and ORR/grade ≥3 TRAEs are presented in **Figures 5A, B**, respectively.

#### 3.4.2 Subgroup analyses according to HBV and HCV infection status

In the subgroup network meta-analyses, a total of nine treatment regimens were included in the OS comparisons and six regimens in the PFS comparisons (**Supplementary Figure S9**). The availability of subgroup-specific data varied across trials and outcomes (**Supplementary Table S3**). Trials contributed to each subgroup-specific network only for those outcomes that were explicitly reported; missing subgroup outcome data were not imputed.

**Figure 6:**
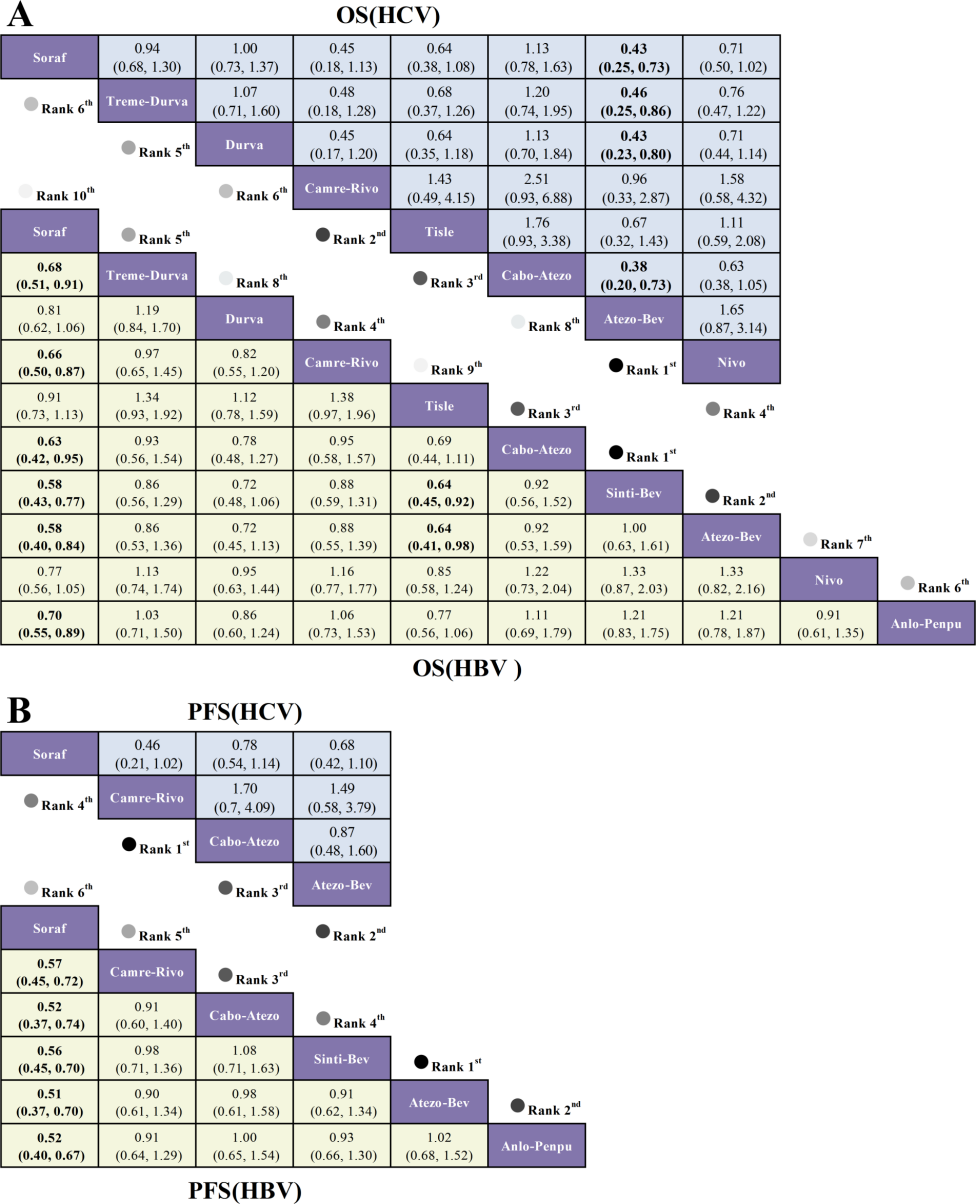
League tables from a Bayesian network meta-analysis comparing first-line immunotherapy regimens in HBV- and HCV-positive advanced hepatocellular carcinoma. **(A)** OS: lower triangle (yellow)=HBV, upper triangle (blue)=HCV; effects shown as HRs with 95% CIs; HR < 1.00 favors the row regimen. **(B)** PFS: lower triangle (yellow)=HBV, upper triangle (blue)=HCV; HRs with 95% CIs; HR < 1.00 favors the row regimen.

For OS, among patients with HBV-positive advanced HCC, sintilimab plus a bevacizumab biosimilar (Sinti-Bev; HR = 0.58, 95% CI 0.43-0.77), atezolizumab plus bevacizumab (Atezo-Bev; HR = 0.58, 95% CI 0.40-0.84), and cabozantinib plus atezolizumab (Cabo-Atezo; HR = 0.63, 95% CI 0.42-0.95) conferred the largest survival advantages relative to sorafenib. In HCV-positive disease, only Atezo-Bev significantly prolonged OS versus sorafenib (HR = 0.43, 95% CI 0.25-0.73); point estimates favored camrelizumab plus rivoceranib (Camre-Rivo; HR = 0.45, 95% CI 0.18-1.13) and tislelizumab (Tisle; HR = 0.64, 95% CI 0.38-1.08), but these differences were not statistically significant (**Figure 6A**).

For PFS, in HBV-positive patients all ICI-based combinations significantly extended PFS versus sorafenib, with the greatest effects for Atezo-Bev (HR = 0.51, 95% CI 0.37-0.70), anlotinib plus penpulimab (Anlo-Penpu; HR = 0.52, 95% CI 0.40-0.67), and Cabo-Atezo (HR = 0.52, 95% CI 0.37-0.74). By contrast, in HCV-positive patients no combination achieved a statistically significant PFS advantage; Camre-Rivo showed a numerically favorable hazard ratio versus sorafenib (HR = 0.46, 95% CI 0.21-1.02) without reaching significance (**Figure 6B**).

**Figure 7:**
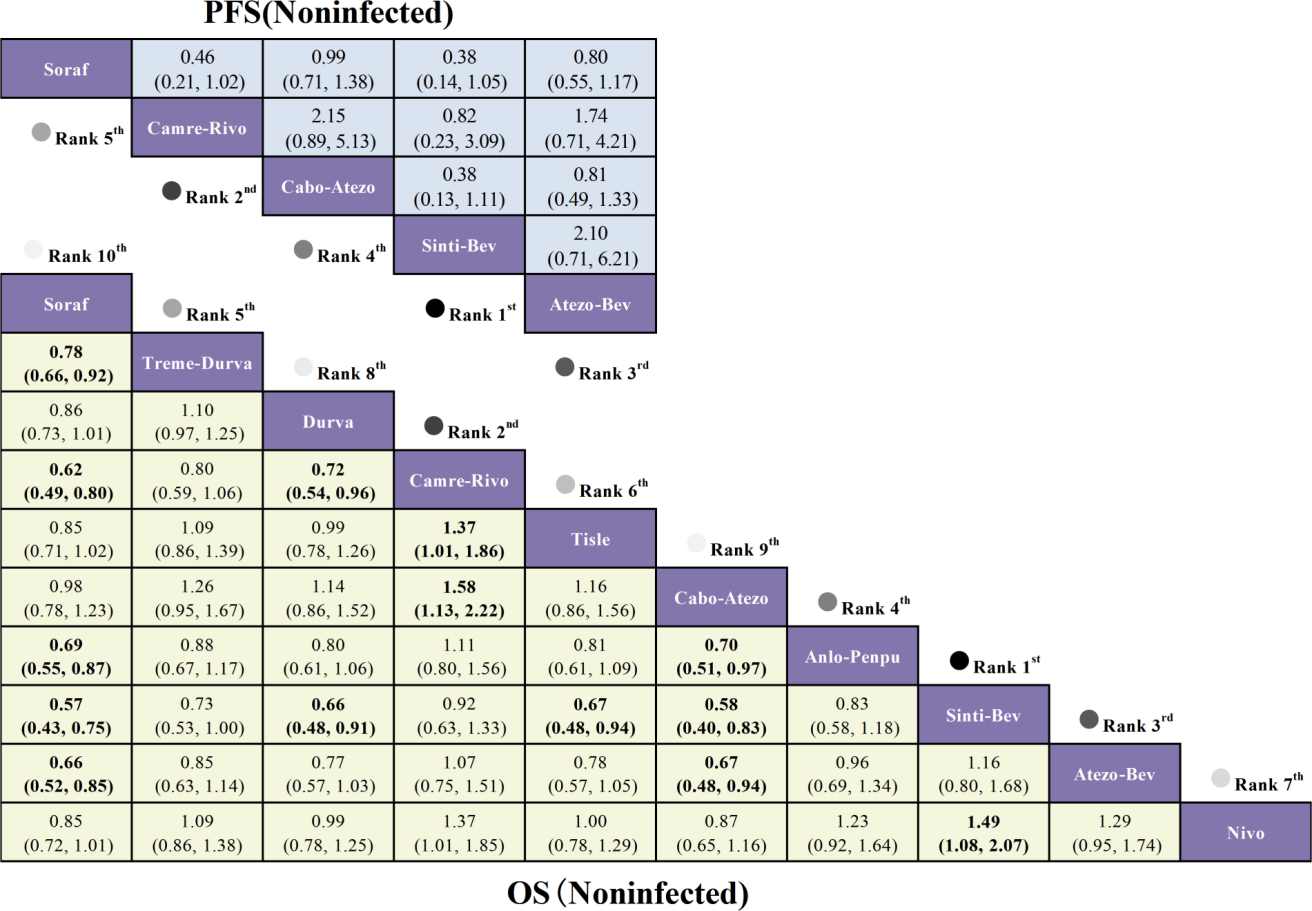
League table of comparative efficacy for immunotherapy regimens in NBNC advanced hepatocellular carcinoma.

In NBNC advanced HCC, OS benefits over sorafenib were most evident with Sinti-Bev (HR = 0.57, 95% CI 0.43-0.75), Camre-Rivo (HR = 0.62, 95% CI 0.49-0.80), and Atezo-Bev (HR = 0.66, 95% CI 0.52-0.85). However, no ICI-based combination produced a statistically significant PFS improvement in this subgroup; Sinti-Bev (HR = 0.38, 95% CI 0.14-1.05) and Camre-Rivo (HR = 0.46, 95% CI 0.21-1.02) showed favorable but non-significant trends (**Figure 7**).

### 3.5 rank

**Figure 8:**
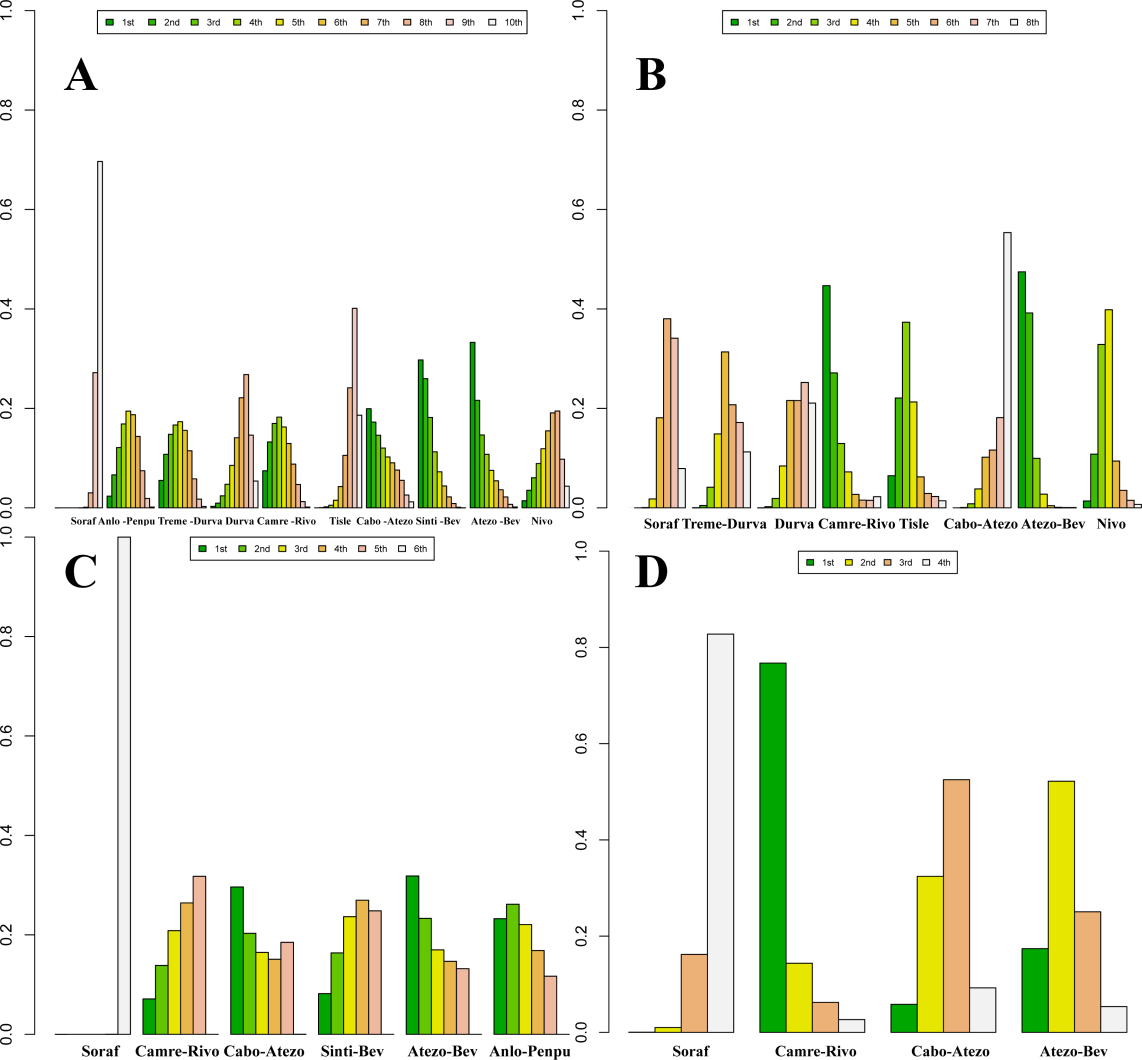
Bayesian rank-probability profiles for the efficacy of first-line immunotherapy regimens in HBV- and HCV-positive advanced hepatocellular carcinoma. **(A)** HBV—OS; **(B)** HBV—PFS; **(C)** HCV—OS; **(D)** HCV—PFS.

**Figure 9:**
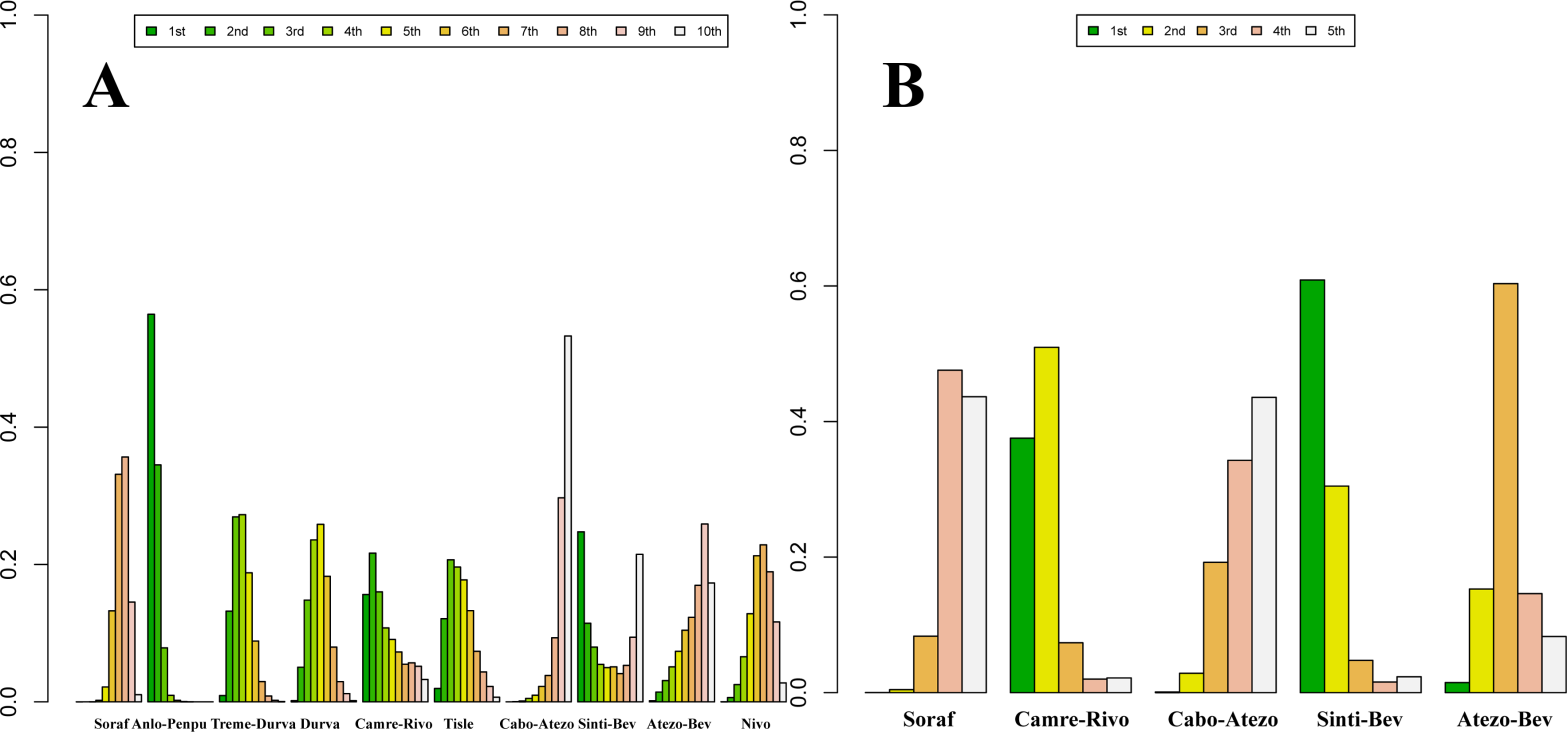
Bayesian rank-probability profiles for the efficacy of first-line immunotherapy regimens in NBNC advanced hepatocellular carcinoma: **(A)** OS; **(B)** PFS.

**Figure 10:**
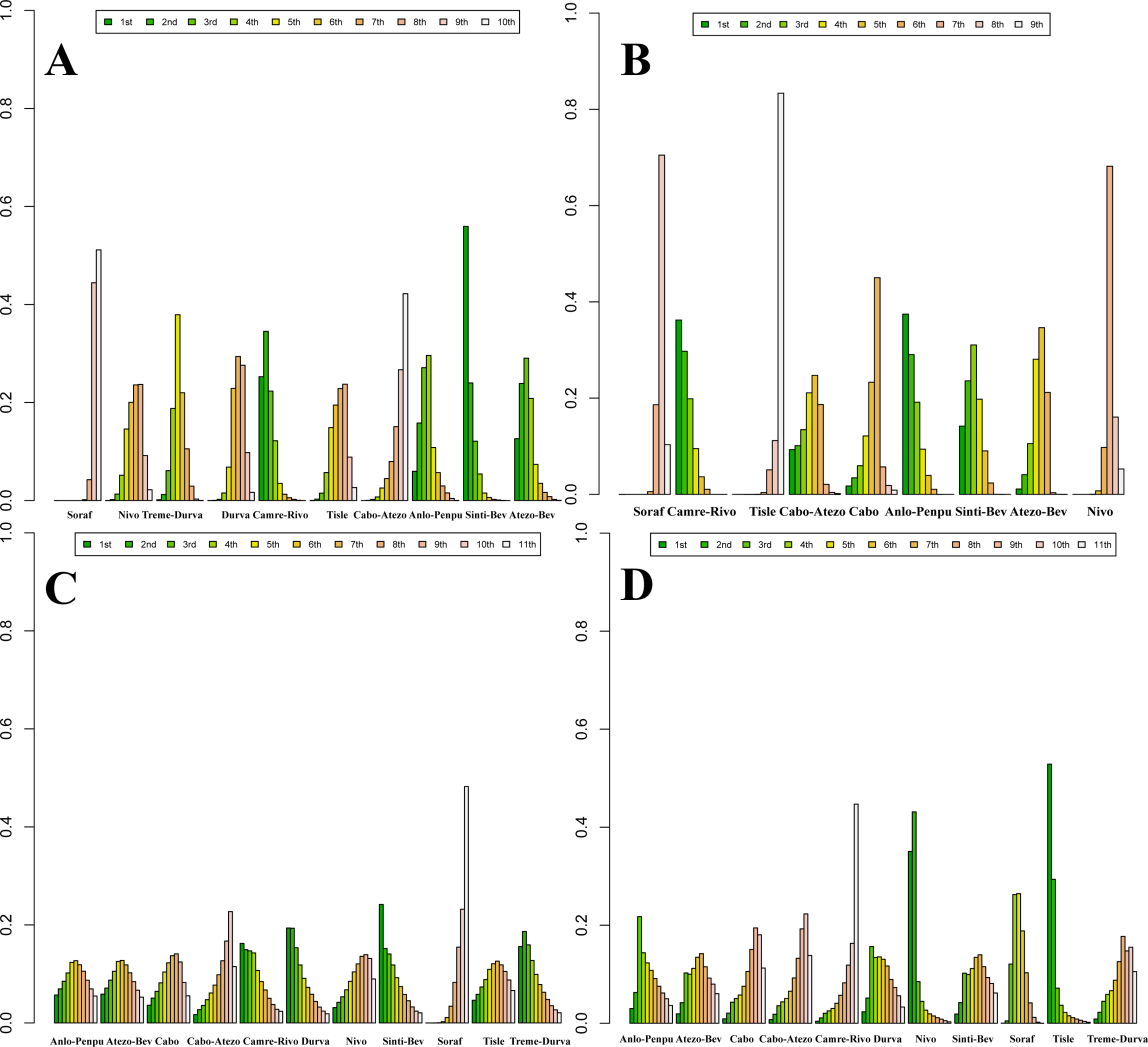
Bayesian rank-probability profiles for the comparative efficacy and safety of first-line immunotherapy in advanced hepatocellular carcinoma: **(A)** OS; **(B)** PFS; **(C)** ORR; **(D)** grade ≥3 adverse events.

Posterior ranking probabilities from the Bayesian NMA are presented as rankograms (**Figures 8**–**10**; **Supplementary Figure S10**-**19**) and indicate the following. In the overall advanced HCC population, Sinti-Bev had the highest probability of ranking first for OS (ranked safest: 56.0%); Anlo-Penpu ranked first for PFS (37.5%); Sinti-Bev ranked first for ORR (24.2%); and tislelizumab (Tisle) had the highest probability of the lowest risk of grade ≥3 TRAEs (53.3%).

In etiology-stratified analyses, Atezo-Bev ranked first for both OS (33.7%) and PFS (32.7%) in HBV-positive disease. In HCV-positive disease, Atezo-Bev ranked first for OS (47.9%), whereas Camre-Rivo ranked first for PFS (75.9%). In HBV/HCV-negative disease, Anlo-Penpu ranked first for OS (56.3%) and Sinti-Bev ranked first for PFS (61.1%).

### 3.6 Convergence, consistency, sensitivity, and publication bias

Model convergence was evaluated using diagnostic plots and the Gelman-Rubin statistic, indicating stable and reproducible MCMC sampling across chains (**Supplementary Figures 30**-**49**). To test the network consistency assumption, we compared DIC values between the consistency and inconsistency models. As shown in **Supplementary Table S4**, DIC differences for all endpoints were <5, suggesting no meaningful inconsistency and acceptable exchangeability between direct and indirect evidence.We also compared DIC values between the primary fixed-effect model and the random-effects sensitivity model. The DIC values were similar across outcomes, with differences <5, indicating no meaningful improvement in model fit with the random-effects specification(**Supplementary Table S5**).

Because the primary NMA used a fixed-effect model, additional random-effects sensitivity analyses were conducted to evaluate the robustness of the findings under a more conservative modeling assumption. The random-effects model yielded effect estimates that were broadly consistent with those of the primary fixed-effect model, with similar directions of effect, statistical significance, and treatment rankings across the main outcomes. These findings indicate that the principal conclusions were robust to alternative modeling assumptions (**Supplementary Figures S20**-**S25**).

For conventional pairwise meta-analyses, leave-one-out influence analyses showed that omitting any single study did not materially alter pooled effects, supporting the robustness of the findings.

Small-study and publication bias were explored with funnel plots for OS, PFS, ORR, and grade ≥3 adverse events; plots appeared approximately symmetrical without conspicuous outliers, suggesting a low likelihood of small-study effects or publication bias (**Supplementary Figures 26**-**29**).

## 4 Discussion

Building upon prior systematic reviews and network meta-analyses of first-line systemic therapies in advanced HCC, including the recent work by Li et al., the present study provides an updated Bayesian NMA that focuses specifically on ICI-based first-line regimens and offers a comprehensive etiologic stratification across HBV-, HCV-, and non-viral subgroups. By incorporating the most recent phase III trials (such as APOLLO and CheckMate 9DW) and jointly evaluating OS, PFS, ORR, and grade ≥3 TRAEs in both overall and etiology-defined populations, our analysis refines the existing evidence base and generates regimen-level hierarchies that are directly relevant to contemporary clinical decision-making.

From a comprehensive appraisal of the evidence, three principal findings emerge.

- In the overall advanced HCC population–as well as in HBV-positive and HCV-positive subgroups–immunotherapy regimens achieved significant gains in OS and PFS versus TKI monotherapy; the overall cohort also showed higher ORR without a significant excess of grade ≥3 TRAEs. Only the NBNC subgroup failed to demonstrate a statistically significant PFS advantage over TKIs.
- In the unstratified population, Sinti-Bev and Camre-Rivo consistently ranked among the top performers for OS, PFS, and ORR, whereas tislelizumab and nivolumab were more likely to yield lower rates of grade ≥3 TRAEs.
- In etiology-specific analyses, Atezo-Bev conferred significant OS and PFS benefits in HBV-positive disease and a significant OS benefit in HCV-positive disease; however, no regimen achieved a statistically significant PFS advantage in the HCV-positive subgroup. In NBNC disease, Sinti-Bev showed the most favorable numerical profile for OS and PFS, although PFS differences were not statistically significant.

The overall superiority of ICI-based combinations over TKI monotherapy is consistent with the recognized biological synergy between anti-VEGF therapy and ICIs. Within the transient “vascular normalization” window, VEGF blockade can improve perfusion and permeability, reduce interstitial pressure, upregulate adhesion molecules, and facilitate effector T-cell trafficking. Concurrently, it may mitigate VEGF-driven dendritic-cell suppression and the enrichment of regulatory T cells (Tregs) and myeloid-derived suppressor cells (MDSCs), thereby creating a microenvironment more permissive to PD-1/PD-L1 blockade—mechanistic underpinnings that plausibly explain the pronounced efficacy observed with Sinti-Bev and Camre-Rivo (23, 24). The finding that tislelizumab and nivolumab monotherapy were associated with lower rates of grade ≥3 treatment-related AEs than sorafenib is also biologically plausible: patients with advanced HCC frequently have cirrhosis, portal hypertension, coagulopathy, and renal impairment. TKIs that inhibit VEGF signaling can precipitate or exacerbate hypertension, proteinuria, and bleeding, are hepatically metabolized with a substantial propensity for drug-drug interactions, and may elevate transaminases or induce cholestasis; in contrast, ICIs do not directly injure endothelium or the angiogenic axis (25, 26).

Subgroup analyses indicate that HBV-positive patients derive additional benefit (OS HR = 0.73 vs 0.79 overall; PFS HR = 0.54 vs 0.70 overall), whereas comparable incremental gains were not observed in HCV-positive or NBNC disease. This pattern aligns with prior evidence: chronic HBV antigen exposure promotes T-cell exhaustion programs, increases Tregs/MDSCs, and upregulates PD-L1 expression (27). Superimposed on anti-VEGF-mediated vascular normalization and improved immune trafficking, ICIs may therefore yield more synergistic antitumor activity in HBV-related HCC (28). By contrast, NBNC tumors more often exhibit an immune-excluded phenotype—classically associated with WNT/β-catenin (CTNNB1) activation—together with metabolic liver-disease-related microenvironments, attenuating survival gains from immunotherapy (29). Our subgroup analyses suggest that the survival benefit of first-line ICI-based regimens in HBV- and HCV-related HCC was consistent across Asia-enriched and non-Asia-enriched trials. While some studies reported greater benefits in Asia-enriched trials, statistical tests for subgroup differences were not significant for OS (P = 0.38) or PFS (P = 0.91), indicating no meaningful effect modification by geographic region. This suggests that the observed benefits of ICI-based therapy are not primarily driven by regional differences in HBV/HCV prevalence, although we acknowledge the potential for residual confounding due to trial-level differences in patient demographics and disease characteristics.

These results are concordant with those of Liu et al., who reported that PD-1/PD-L1 inhibitors improve OS, PFS, and ORR versus TKIs, though that analysis relied predominantly on retrospective studies and is thus more vulnerable to selection bias, residual confounding, and publication bias (30). They also align with the meta-analysis by Huang et al., which demonstrated significant OS and PFS benefits for immunotherapy in HBV/HCV-positive patients (31). Extending this literature, the present work incorporates ten RCTs (five additional trials beyond Huang et al.) and applies an NMA framework to compare first-line regimens head-to-head, yielding a more granular, regimen-level hierarchy and practical guidance on optimal first-line choices across etiologies.

This study has several limitations. First, several included trials were open-label, which may introduce observer and reporting bias, particularly for subjective endpoints (e.g., adverse drug reactions and treatment discontinuation), potentially leading to measurement heterogeneity. However, most trials used objective outcomes, such as OS, as the primary endpoint and commonly employed blinded independent radiologic review, which mitigates the impact on key outcomes. Second, enrollment was imbalanced by region and etiology, with approximately 60% of participants being Asian, reflecting global HCC epidemiology but leaving populations of Black/African ancestry underrepresented (<5%), which limits generalizability to these groups. Additionally, the imbalance in trial composition by geographic region and viral etiology, especially the higher proportion of Asian participants in certain trials, may have introduced residual confounding, which cannot be fully excluded. While our analyses suggest that the OS and PFS benefits of ICI-based regimens are broadly consistent across Asia-enriched and non-Asia-enriched trials, unmeasured demographic or clinical factors may still influence the observed treatment effects. Finally, some regimens, such as nivolumab and anlotinib plus penpulimab, are currently supported by only a single RCT. Although these studies were rigorously designed with sufficient sample sizes (≥300 participants per trial), additional confirmatory evidence would further strengthen confidence in these rankings.

In summary, first-line ICI-based therapy provides meaningful survival gains over TKI monotherapy in advanced HCC, with particularly robust benefits in virally mediated disease. This NMA delineates regimen-specific advantages by highlighting Sinti-Bev and Camre-Rivo in the overall population and Atezo-Bev as the most consistent regimen in virally mediated subgroups, particularly in HBV-positive disease, while also underscoring favorable safety signals for PD-1-based monotherapy. These findings offer an evidence-based framework to tailor first-line choices by etiology and clinical priorities.

## Data Availability

The original contributions presented in the study are included in the article/**Supplementary Material**. Further inquiries can be directed to the corresponding author.
