# Supplementary material for: First-line immunotherapy for advanced hepatocellular carcinoma: a network meta-analysis of randomized trials with overall and HBV/HCV-stratified efficacy and safety
Source: Front Immunol. 2026 May 14;17:1693251. doi: 10.3389/fimmu.2026.1693251 (PMC13215822; doi:10.3389/fimmu.2026.1693251)
Supplement: Supplementary file 1 [file Table1.docx]

First-Line Immunotherapy for Advanced Hepatocellular Carcinoma: A Network Meta-analysis of Randomized Trials with Overall and HBV/HCV-Stratified Efficacy and Safety

| **Table of Contents** | | |
| --- | --- | --- |
| Title | Content | page |
| Table S1 | PRISMA NMA Checklist of Items to Include When Reporting a Systematic Review Involving a Network Meta-analysis | 4-7 |
| Table S2 | Literature Search Strategy | 7 |
| Table S3 | Availability of etiology-specific subgroup outcomes across included trials | 8 |
| Table S4 | Consistency and Inconsistency Model Fit Comparison and Heterogeneity Assessment Across Endpoints in the Bayesian Network Meta-Analysis | 8 |
| Table S5 | Fixed-Effect and Random-Effects Model Fit Comparison and Heterogeneity Assessment Across Endpoints in the Bayesian Network Meta-Analysis | 9 |
| Figure S1 | Meta-analysis forest plot for overall survival comparing immunotherapy with tyrosine kinase inhibitors in advanced HCC, overall and by viral etiology (HBV, HCV, NBNC) | 9 |
| Figure S2 | Meta-analysis forest plot for progression-free survival comparing immunotherapy with tyrosine kinase inhibitors in advanced HCC, overall and by viral etiology (HBV, HCV, NBNC). | 10 |
| Figure S3 | Forest plot for ORR comparing immunotherapy with single-agent TKIs in advanced hepatocellular carcinoma. | 11 |
| Figure S4 | Forest plot for grade ≥3 AEs comparing immunotherapy with single-agent TKIs in advanced hepatocellular carcinoma | 12 |
| Figure S5 | Forest plot of OS for HBV-related HCC: first-line ICI-based regimens versus TKIs in Asia-enriched and non–Asia-enriched trials | 12 |
| Figure S6 | Forest plot of OS for HCV-related HCC: first-line ICI-based regimens versus TKIs in Asia-enriched and non–Asia-enriched trials | 13 |
| Figure S7 | Forest plot of PFS for HBV-related HCC: first-line ICI-based regimens versus TKIs in Asia-enriched and non–Asia-enriched trials | 13 |
| Figure S8 | Forest plot of PFS for HCV-related HCC: first-line ICI-based regimens versus TKIs in Asia-enriched | 14 |
| Figure S9 | Evidence networks for the comparative efficacy of ICI-based regimens in advanced HCC across HBV, HCV, and NBNC subgroups: (A) OS; (B) PFS. | 14 |
| Figure S10 | Rank Probability Heatmap of Treatment Regimens for OS in Advanced HCC. | 15 |
| Figure S11 | Rank Probability Heatmap of Treatment Regimens for PFS in Advanced HCC. | 15 |
| Figure S12 | Rank Probability Heatmap of Treatment Regimens for ORR in Advanced HCC. | 15 |
| Figure S13 | Rank Probability Heatmap of Treatment Regimens for AE≥3 in Advanced HCC. | 16 |
| Figure S14 | Rank Probability Heatmap of Treatment Regimens for OS in HBV-positive Advanced HCC. | 16 |
| Figure S15 | Rank Probability Heatmap of Treatment Regimens for OS in HCV-positive Advanced HCC. | 17 |
| Figure S16 | Rank Probability Heatmap of Treatment Regimens for OS in NBNCAdvanced HCC. | 17 |
| Figure S17 | Rank Probability Heatmap of Treatment Regimens for PFS in HBV-positive Advanced HCC. | 18 |
| Figure S18 | Rank Probability Heatmap of Treatment Regimens for PFS in HCV-positive Advanced HCC. | 18 |
| Figure S19 | Rank Probability Heatmap of Treatment Regimens for PFS in NBNC Advanced HCC. | 19 |
| Figure S20 | League tables from a Bayesian random-effects network meta-analysis comparing first-line immunotherapy regimens in advanced hepatocellular carcinoma.  (A) Lower triangle (yellow): OS—HRs with 95% CIs; upper triangle (blue): PFS—HRs with 95% CIs. For both OS and PFS, HR < 1.00 favors the row regimen, indicating greater survival benefit.  (B) Lower triangle (yellow): ORR—ORs with 95% CIs, where OR > 1.00 favors the row regimen; upper triangle (blue): grade ≥3 AEs—ORs with 95% CIs, where OR < 1.00 indicates fewer severe adverse events and thus better safety for the row regimen. | 20 |
| Figure S21 | League tables from a Bayesian random-effects network meta-analysis comparing first-line immunotherapy regimens in HBV- and HCV-positive advanced hepatocellular carcinoma.  (A) OS: lower triangle (yellow) = HBV, upper triangle (blue) = HCV; effects are presented as HRs with 95% CIs; HR < 1.00 favors the row regimen.  (B) PFS: lower triangle (yellow) = HBV, upper triangle (blue) = HCV; effects are presented as HRs with 95% CIs; HR < 1.00 favors the row regimen. | 21 |
| Figure S22 | League table of comparative efficacy from a random-effects network meta-analysis of immunotherapy regimens in NBNC advanced hepatocellular carcinoma | 22 |
| Figure S23 | Bayesian random-effects rank-probability profiles for the comparative efficacy and safety of first-line immunotherapy in advanced hepatocellular carcinoma: (A) OS; (B) PFS; (C) ORR; (D) grade ≥3 adverse events | 23 |
| Figure S24 | Rank-probability profiles from a Bayesian random-effects network meta-analysis for the efficacy of first-line immunotherapy regimens in HBV- and HCV-positive advanced hepatocellular carcinoma: (A) HBV—OS; (B) HBV—PFS; (C) HCV—OS; (D) HCV—PFS | 24 |
| Figure S25 | Rank-probability profiles from a Bayesian random-effects network meta-analysis for the efficacy of first-line immunotherapy regimens in NBNC advanced hepatocellular carcinoma: (A) OS; (B) PFS. | 24 |
| Figure S26 | Funnel plot for OS in advanced hepatocellular carcinoma: immunotherapy vs tyrosine kinase inhibitors. | 25 |
| Figure S27 | Funnel plot for PFS in advanced hepatocellular carcinoma: immunotherapy vs tyrosine kinase inhibitors. | 25 |
| Figure S28 | Funnel plot for ORR in advanced hepatocellular carcinoma: immunotherapy vs tyrosine kinase inhibitors. | 26 |
| Figure S29 | Funnel plot for AEs≥3 in advanced hepatocellular carcinoma: immunotherapy vs tyrosine kinase inhibitors. | 26 |
| Figure S30 | MCMC trace and posterior density plots for OS in advanced hepatocellular carcinoma: immunotherapy vs tyrosine kinase inhibitors. | 27 |
| Figure S31 | MCMC trace and posterior density plots for PFS in advanced hepatocellular carcinoma: immunotherapy vs tyrosine kinase inhibitors. | 28 |
| Figure S32 | MCMC trace and posterior density plots for ORR in advanced hepatocellular carcinoma: immunotherapy vs tyrosine kinase inhibitors. | 29 |
| Figure S33 | MCMC trace and posterior density plots for AEs≥3 in advanced hepatocellular carcinoma: immunotherapy vs tyrosine kinase inhibitors. | 30 |
| Figure S34 | MCMC trace and posterior density plots for OS in HBV-positive advanced hepatocellular carcinoma: immunotherapy vs tyrosine kinase inhibitors. | 31 |
| Figure S35 | MCMC trace and posterior density plots for OS in HCV-positive advanced hepatocellular carcinoma: immunotherapy vs tyrosine kinase inhibitors. | 32 |
| Figure S36 | MCMC trace and posterior density plots for OS in NBNC advanced hepatocellular carcinoma: immunotherapy vs tyrosine kinase inhibitors. | 32 |
| Figure S37 | MCMC trace and posterior density plots for PFS in HBV-positive advanced hepatocellular carcinoma: immunotherapy vs tyrosine kinase inhibitors. | 33 |
| Figure S38 | MCMC trace and posterior density plots for PFS in HCV-positive advanced hepatocellular carcinoma: immunotherapy vs tyrosine kinase inhibitors. | 34 |
| Figure S39 | MCMC trace and posterior density plots for PFS in NBNC advanced hepatocellular carcinoma: immunotherapy vs tyrosine kinase inhibitors. | 34 |
| Figure S40 | Convergence diagnostics for OS in advanced hepatocellular carcinoma: immunotherapy vs tyrosine kinase inhibitors. | 35 |
| Figure S41 | Convergence diagnostics for PFS in advanced hepatocellular carcinoma: immunotherapy vs tyrosine kinase inhibitors. | 35 |
| Figure S42 | Convergence diagnostics for ORR in advanced hepatocellular carcinoma: immunotherapy vs tyrosine kinase inhibitors. | 36 |
| Figure S43 | Convergence diagnostics for AEs≥3 in advanced hepatocellular carcinoma: immunotherapy vs tyrosine kinase inhibitors. | 37 |
| Figure S44 | Convergence diagnostics for OS in HBV-positive advanced hepatocellular carcinoma: immunotherapy vs tyrosine kinase inhibitors. | 37 |
| Figure S45 | Convergence diagnostics for OS in HCV-positive advanced hepatocellular carcinoma: immunotherapy vs tyrosine kinase inhibitors. | 38 |
| Figure S46 | Convergence diagnostics for OS in NBNC advanced hepatocellular carcinoma: immunotherapy vs tyrosine kinase inhibitors. | 38 |
| Figure S47 | Convergence diagnostics for PFS in HBV-positive advanced hepatocellular carcinoma: immunotherapy vs tyrosine kinase inhibitors. | 39 |
| Figure S48 | Convergence diagnostics for PFS in HCV-positive advanced hepatocellular carcinoma: immunotherapy vs tyrosine kinase inhibitors. | 39 |
| Figure S49 | Convergence diagnostics for PFS in NBNC advanced hepatocellular carcinoma: immunotherapy vs tyrosine kinase inhibitors. | 40 |

Table S1 PRISMA NMA Checklist of Items to Include When Reporting a Systematic Review Involving a Network Meta-analysis

| **Section/Topic** | **Item #** | **Checklist Item** | **Reported on Page #** |
| --- | --- | --- | --- |
| **TITLE** |  |  |  |
| Title | 1 | Identify the report as a systematic review *incorporating*  *anetwork meta-analysis (or related form of meta-analysis).* | **1** |
|  |  |  |  |
| **ABSTRACT** |  |  |  |
| Structured summary | 2 | Provide a structured summary including, as applicable:  **Background:** main objectives  **Methods:** data sources; study eligibility criteria, participants, and interventions; study appraisal; and *synthesis methods, such as network meta-analysis.*  **Results:** number of studies and participants identified; summary estimates with corresponding confidence/credible intervals; *treatment rankings may also be discussed. Authors may choose to summarize pairwise comparisons against a chosen treatment included in their analyses for brevity.*  **Discussion/Conclusions:** limitations; conclusions and implications of findings.  **Other:** systematic review registration number with registry name. | 1-2 |
|  |  |  |  |
| **INTRODUCTION** |  |  |  |
| Rationale | 3 | Describe the rationale for the review in the context of what is already known*, including mention of why a network meta-analysis has been conducted.* | **2** |
| Objectives | 4 | Provide an explicit statement of questions being addressed, with reference to participants, interventions, comparisons, outcomes, and study design (PICOS). | 3 |
|  |  |  |  |
| **METHODS** |  |  |  |
| Protocol and registration | 5 | Indicate whether a review protocol exists and if and where it can be accessed (e.g., Web address); and, if available, provide registration information, including registration number. | 3 |
| Eligibility criteria | 6 | Specify study characteristics (e.g., PICOS, length of follow-up) and report characteristics (e.g., years considered, language, publication status) used as criteria for eligibility, giving rationale. *Clearly describe eligible treatments included in the treatment network, and note whether any have been clustered or merged into the same node (with justification).* | 3 |
| Information sources | 7 | Describe all information sources (e.g., databases with dates of coverage, contact with study authors to identify additional studies) in the search and date last searched. | 3 |
| Search | 8 | Present full electronic search strategy for at least one database, including any limits used, such that it could be repeated. | 3，Supplementary  TableS2 |
| Study selection | 9 | State the process for selecting studies (i.e., screening, eligibility, included in systematic review, and, if applicable, included in the meta-analysis). | **3** |
| Data collection process | 10 | Describe method of data extraction from reports (e.g., piloted forms, independently, in duplicate) and any processes for obtaining and confirming data from investigators. | 4 |
| Data items | 11 | List and define all variables for which data were sought (e.g., PICOS, funding sources) and any assumptions and simplifications made. | 4 |
| **Geometry of the network** | **S1** | Describe methods used to explore the geometry of the treatment network under study and potential biases related to it. This should include how the evidence base has been graphically summarized for presentation, and what characteristics were compiled and used to describe the evidence base to readers. | **4** |
| Risk of bias within individual studies | 12 | Describe methods used for assessing risk of bias of individual studies (including specification of whether this was done at the study or outcome level), and how this information is to be used in any data synthesis. | 4 |
| Summary measures | 13 | State the principal summary measures (e.g., risk ratio, difference in means). *Also describe the use of additional summary measures assessed, such as treatment rankings and surface under the cumulative ranking curve (SUCRA) values, as well as modified approaches used to present summary findings from meta-analyses.* | 4 |
| Planned methods of analysis | 14 | Describe the methods of handling data and combining results of studies for each network meta-analysis. This should include, but not be limited to:   - *Handling of multi-arm trials;* - *Selection of variance structure;* - *Selection of prior distributions in Bayesian analyses; and* - *Assessment of model fit.* | 4-5 |
| **Assessment of Inconsistency** | **S2** | Describe the statistical methods used to evaluate the agreement of direct and indirect evidence in the treatment network(s) studied. Describe efforts taken to address its presence when found. | 4 |
| Risk of bias across studies | 15 | Specify any assessment of risk of bias that may affect the cumulative evidence (e.g., publication bias, selective reporting within studies). | 4 |
| Additional analyses | 16 | Describe methods of additional analyses if done, indicating which were pre-specified. This may include, but not be limited to, the following:   - Sensitivity or subgroup analyses; - Meta-regression analyses; - *Alternative formulations of the treatment network; and* - *Use of alternative prior distributions for Bayesian analyses (if applicable).* | 4-5 |
| **RESULTS†** |  |  |  |
| Study selection | 17 | Give numbers of studies screened, assessed for eligibility, and included in the review, with reasons for exclusions at each stage, ideally with a flow diagram. | 5-6 |
| **Presentation of network structure** | **S3** | Provide a network graph of the included studies to enable visualization of the geometry of the treatment network. | 14,16 |
| **Summary of network geometry** | **S4** | Provide a brief overview of characteristics of the treatment network. This may include commentary on the abundance of trials and randomized patients for the different interventions and pairwise comparisons in the network, gaps of evidence in the treatment network, and potential biases reflected by the network structure. | 13,16 |
| Study characteristics | 18 | For each study, present characteristics for which data were extracted (e.g., study size, PICOS, follow-up period) and provide the citations. | 7-9,Table 1 |
| Risk of bias within studies | 19 | Present data on risk of bias of each study and, if available, any outcome level assessment. | 10,Figure 2 |
| Results of individual studies | 20 | For all outcomes considered (benefits or harms), present, for each study: 1) simple summary data for each intervention group, and 2) effect estimates and confidence intervals. *Modified approaches may be needed to deal with information from larger networks.* | 11-18 |
| Synthesis of results | 21 | Present results of each meta-analysis done, including confidence/credible intervals. *In larger networks, authors may focus on comparisons versus a particular comparator (e.g. placebo or standard care), with full findings presented in an appendix. League tables and forest plots may be considered to summarize pairwise comparisons.* If additional summary measures were explored (such as treatment rankings), these should also be presented. | 11-19 |
| **Exploration for inconsistency** | **S5** | Describe results from investigations of inconsistency. This may include such information as measures of model fit to compare consistency and inconsistency models, *P* values from statistical tests, or summary of inconsistency estimates from different parts of the treatment network. | 21 |
| Risk of bias across studies | 22 | Present results of any assessment of risk of bias across studies for the evidence base being studied. | 21 |
| Results of additional analyses | 23 | Give results of additional analyses, if done (e.g., sensitivity or subgroup analyses, meta-regression analyses, alternative network geometries studied, alternative choice of prior distributions for Bayesian analyses, and so forth). | 21 |
|  |  |  |  |
| **DISCUSSION** |  |  |  |
| Summary of evidence | 24 | Summarize the main findings, including the strength of evidence for each main outcome; consider their relevance to key groups (e.g., healthcare providers, users, and policy-makers). | 21,22 |
| Limitations | 25 | Discuss limitations at study and outcome level (e.g., risk of bias), and at review level (e.g., incomplete retrieval of identified research, reporting bias). *Comment on the validity of the assumptions, such as transitivity and consistency. Comment on any concerns regarding network geometry (e.g., avoidance of certain comparisons).* | 22 |
| Conclusions | 26 | Provide a general interpretation of the results in the context of other evidence, and implications for future research. | 1 |
|  |  |  |  |
| **FUNDING** |  |  |  |
| Funding | 27 | Describe sources of funding for the systematic review and other support (e.g., supply of data); role of funders for the systematic review. This should also include information regarding whether funding has been received from manufacturers of treatments in the network and/or whether some of the authors are content experts with professional conflicts of interest that could affect use of treatments in the network. | NA |

PICOS = population, intervention, comparators, outcomes, study design.

* Text in italics indicate S wording specific to reporting of network meta-analyses that has been added to guidance from the PRISMA statement.

† Authors may wish to plan for use of appendices to present all relevant information in full detail for items in this section.

| **Table S2.Literature Search Strategy** | |
| --- | --- |
| **Pubmed** | (("Carcinoma, Hepatocellular"[Mesh] OR hepatocellular carcinoma[tiab] OR HCC[tiab] OR (liver[tiab] AND cancer[tiab]))  AND  ("Randomized Controlled Trial"[Publication Type] OR randomized[tiab] OR randomised[tiab] OR trial[tiab])  AND  ("Immune Checkpoint Inhibitors"[Mesh] OR immune checkpoint inhibitor*[tiab]  OR PD1[tiab] OR PD-1[tiab] OR PDL1[tiab] OR PD-L1[tiab] OR CTLA4[tiab] OR CTLA-4[tiab]  OR tremelimumab[tiab] OR durvalumab[tiab] OR camrelizumab[tiab] OR tislelizumab[tiab]  OR pembrolizumab[tiab] OR ipilimumab[tiab] OR nivolumab[tiab] OR sintilimab[tiab]  OR atezolizumab[tiab] OR penpulimab[tiab])) |
| **Web of Science** | TS=(("hepatocellular carcinoma" OR HCC OR (liver AND cancer))  AND  (randomized OR randomised OR "randomized controlled trial" OR trial)  AND  ("immune checkpoint inhibitor*" OR PD1 OR "PD-1" OR PDL1 OR "PD-L1" OR CTLA4 OR "CTLA-4"  OR tremelimumab OR durvalumab OR camrelizumab OR tislelizumab  OR pembrolizumab OR ipilimumab OR nivolumab OR sintilimab  OR atezolizumab OR penpulimab)) |
| **Cochrane** | ([mh "Carcinoma, Hepatocellular"] OR hepatocellular carcinoma:ti,ab,kw OR HCC:ti,ab,kw OR (liver:ti,ab,kw AND cancer:ti,ab,kw))  AND  ([mh "Randomized Controlled Trials as Topic"] OR randomized:ti,ab,kw OR randomised:ti,ab,kw OR trial:ti,ab,kw)  AND  ([mh "Immune Checkpoint Inhibitors"] OR "immune checkpoint inhibitor*":ti,ab,kw  OR PD1:ti,ab,kw OR PD-1:ti,ab,kw OR PDL1:ti,ab,kw OR PD-L1:ti,ab,kw OR CTLA4:ti,ab,kw OR CTLA-4:ti,ab,kw  OR tremelimumab:ti,ab,kw OR durvalumab:ti,ab,kw OR camrelizumab:ti,ab,kw OR tislelizumab:ti,ab,kw  OR pembrolizumab:ti,ab,kw OR ipilimumab:ti,ab,kw OR nivolumab:ti,ab,kw OR sintilimab:ti,ab,kw  OR atezolizumab:ti,ab,kw OR penpulimab:ti,ab,kw) |
| **Embase** | ('hepatocellular carcinoma'/exp OR 'hepatocellular carcinoma':ti,ab OR HCC:ti,ab OR (liver:ti,ab AND cancer:ti,ab))  AND  ('randomized controlled trial'/exp OR randomized:ti,ab OR randomised:ti,ab OR trial:ti,ab)  AND  ('immune checkpoint inhibitor'/exp OR 'immune checkpoint inhibitor*':ti,ab  OR PD1:ti,ab OR 'PD-1':ti,ab OR PDL1:ti,ab OR 'PD-L1':ti,ab OR CTLA4:ti,ab OR 'CTLA-4':ti,ab  OR tremelimumab:ti,ab OR durvalumab:ti,ab OR camrelizumab:ti,ab OR tislelizumab:ti,ab  OR pembrolizumab:ti,ab OR ipilimumab:ti,ab OR nivolumab:ti,ab OR sintilimab:ti,ab  OR atezolizumab:ti,ab OR penpulimab:ti,ab) |

**Table S3. Availability of etiology-specific subgroup outcomes across included trials**

| **RCT** | **HBV OS** | **HCV OS** | **NBNC OS** | **HBV PFS** | **HCV PFS** | **NBNC PFS** |
| --- | --- | --- | --- | --- | --- | --- |
| HIMALAYA | ✓ | ✓ | ✓ |  |  |  |
| CARES-310 | ✓ | ✓ | ✓ | ✓ | ✓ | ✓ |
| RATIONALE-301 | ✓ | ✓ | ✓ |  |  |  |
| COSMIC-312 | ✓ | ✓ | ✓ | ✓ | ✓ | ✓ |
| APOLLO | ✓ |  | ✓ | ✓ |  |  |
| ORIENT-32 | ✓ |  | ✓ | ✓ |  | ✓ |
| IMbrave150 | ✓ | ✓ | ✓ | ✓ | ✓ | ✓ |
| CheckMate 459 | ✓ | ✓ | ✓ |  |  |  |

**Table S4. Consistency and Inconsistency Model Fit Comparison and Heterogeneity Assessment Across Endpoints in the Bayesian Network Meta-Analysis**

| **Endpoints** | **Model Type** | **Dbar** | **pD** | **DIC** | **I**2 |
| --- | --- | --- | --- | --- | --- |
| OS | Consistency | 9.00 | 9.00 | 18.00 | 11% |
|  | Inconsistency | 8.98 | 8.98 | 17.96 | 11% |
| PFS | Consistency | 7.99 | 7.99 | 15.98 | 12% |
|  | Inconsistency | 7.98 | 7.98 | 15.96 | 12% |
| ORR | Consistency | 18.13 | 18.13 | 36.26 | 6% |
|  | Inconsistency | 18.09 | 17.99 | 36.08 | 6% |
| AE≥3 | Consistency | 17.97 | 17.93 | 35.90 | 5% |
|  | Inconsistency | 17.98 | 17.94 | 35.92 | 5% |
| HBV OS | Consistency | 9.02 | 9.02 | 18.04 | 11% |
|  | Inconsistency | 8.99 | 8.99 | 17.98 | 11% |
| HCV OS | Consistency | 7.00 | 7.00 | 14.00 | 14% |
|  | Inconsistency | 7.01 | 7.01 | 14.02 | 14% |
| NBNC OS | Consistency | 9.00 | 9.00 | 18.00 | 11% |
|  | Inconsistency | 9.01 | 9.01 | 18.02 | 11% |
| HBV PFS | Consistency | 5.02 | 5.02 | 10.04 | 20% |
|  | Inconsistency | 5.00 | 5.00 | 10.00 | 20% |
| HCV PFS | Consistency | 2.97 | 2.97 | 5.94 | 33% |
|  | Inconsistency | 2.95 | 2.95 | 5.90 | 33% |
| NBNC PFS | Consistency | 4.00 | 4.00 | 8.00 | 25% |
|  | Inconsistency | 3.99 | 3.99 | 7.98 | 25% |

**Table S5. Fixed-Effect and Random-Effects Model Fit Comparison and Heterogeneity Assessment Across Endpoints in the Bayesian Network Meta-Analysis**

| **Endpoints** | **Model Type** | **Dbar** | **pD** | **DIC** | **I^2^** |
| --- | --- | --- | --- | --- | --- |
| OS | Random | 8.97 | 8.99 | 17.96 | 11% |
|  | Fixed | 8.98 | 8.98 | 17.96 | 11% |
| PFS | Random | 8.01 | 8.03 | 16.04 | 12% |
|  | Fixed | 7.98 | 7.98 | 15.96 | 12% |
| ORR | Random | 18.11 | 18.15 | 36.26 | 6% |
|  | Fixed | 18.09 | 17.99 | 36.08 | 6% |
| AE≥3 | Random | 17.98 | 17.95 | 35.93 | 5% |
|  | Fixed | 17.98 | 17.94 | 35.92 | 5% |
| HBV OS | Random | 9.01 | 9.05 | 18.06 | 11% |
|  | Fixed | 8.99 | 8.99 | 17.98 | 11% |
| HCV OS | Random | 7.05 | 7.03 | 14.08 | 14% |
|  | Fixed | 7.01 | 7.01 | 14.02 | 14% |
| NBNC OS | Random | 9.02 | 9.05 | 18.07 | 11% |
|  | Fixed | 9.01 | 9.01 | 18.02 | 11% |
| HBV PFS | Random | 5.05 | 4.98 | 10.03 | 20% |
|  | Fixed | 5 | 5 | 10 | 20% |
| HCV PFS | Random | 2.98 | 2.99 | 5.97 | 33% |
|  | Fixed | 2.95 | 2.95 | 5.9 | 33% |
| NBNC PFS | Random | 4.02 | 4.05 | 8.07 | 25% |
|  | Fixed | 3.99 | 3.99 | 7.98 | 25% |


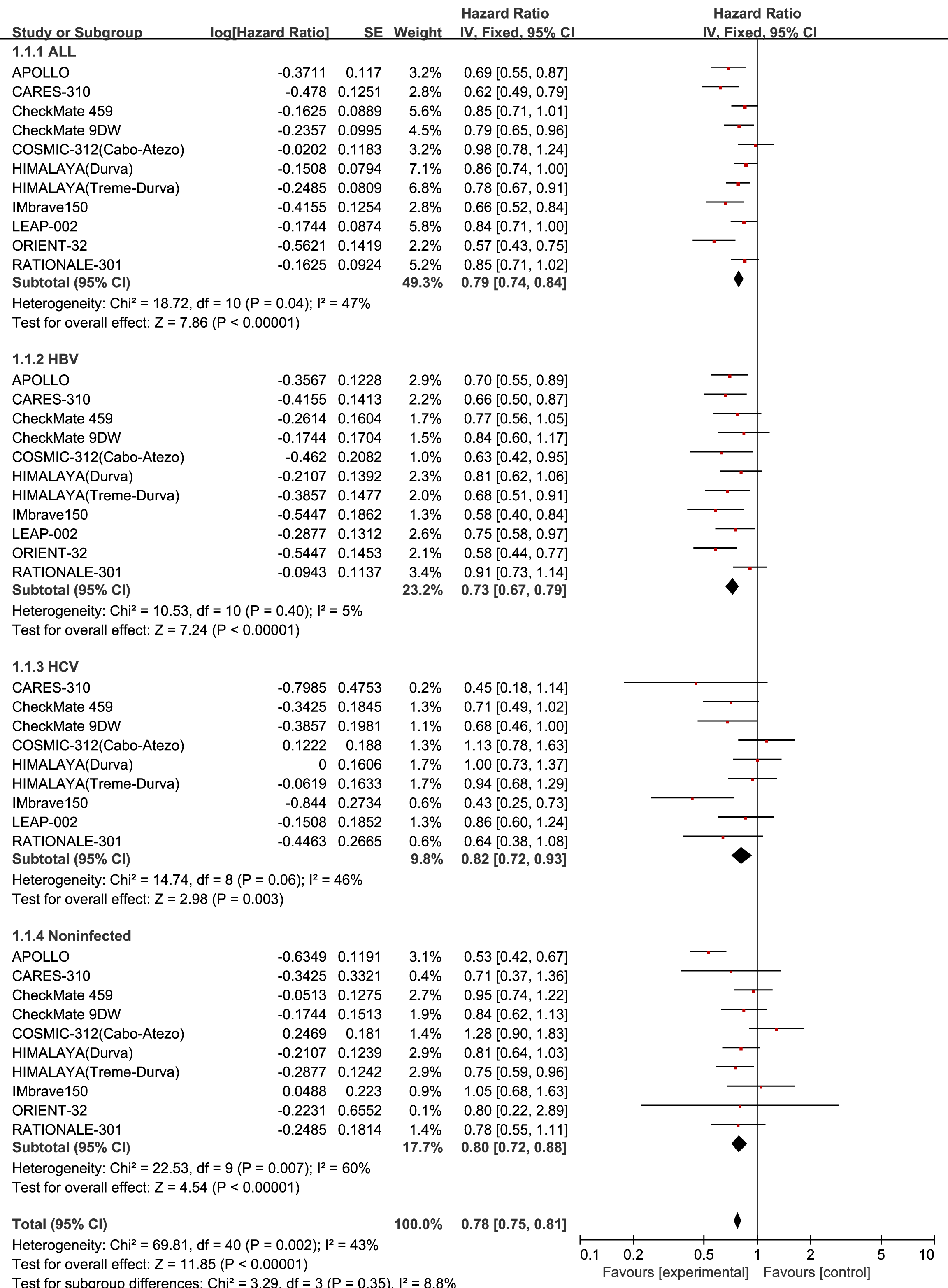


Figure S1. Meta-analysis forest plot for overall survival comparing immunotherapy with tyrosine kinase inhibitors in advanced HCC, overall and by viral etiology (HBV, HCV, NBNC)


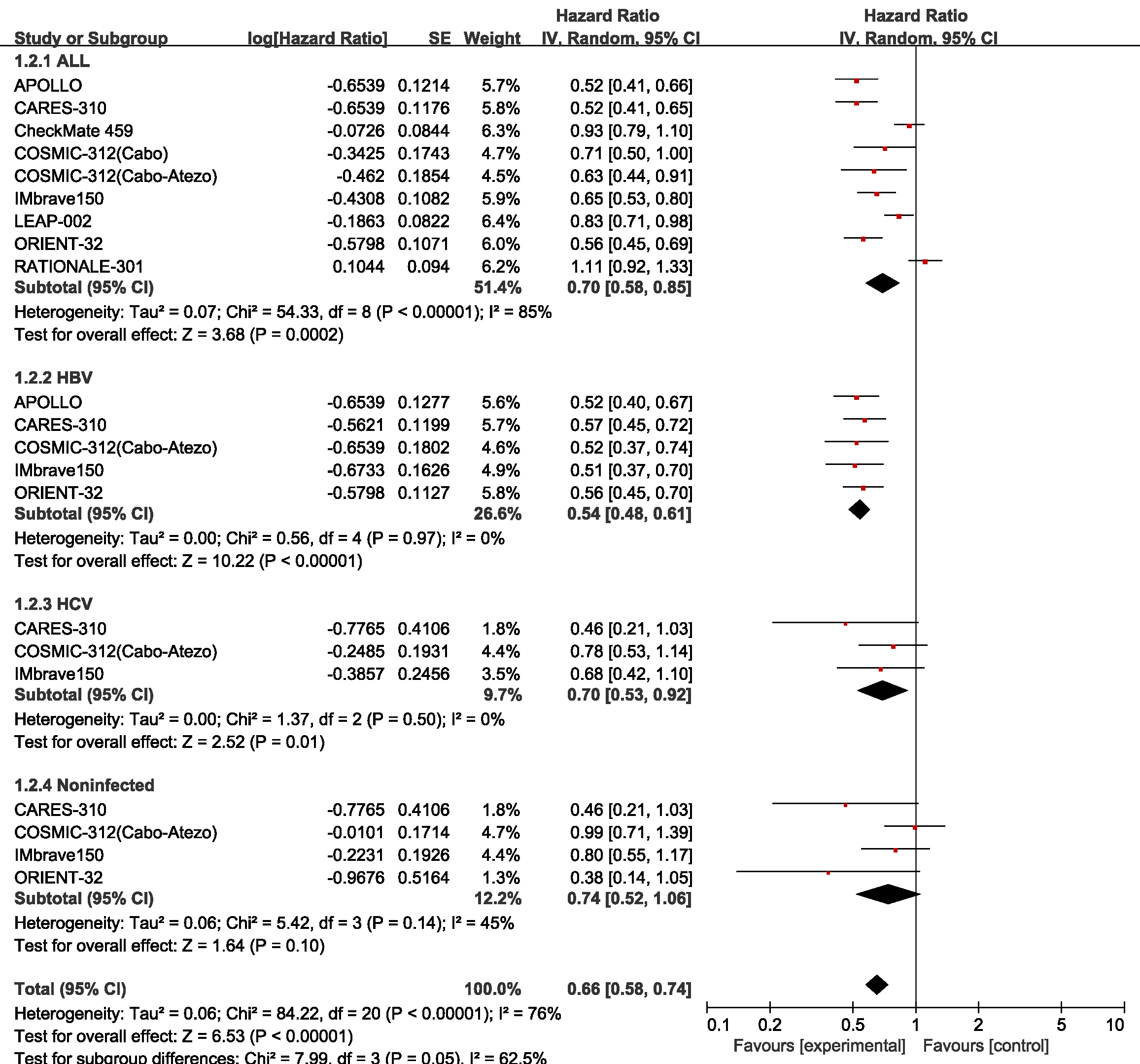


Figure S2. Meta-analysis forest plot for progression-free survival comparing immunotherapy with tyrosine kinase inhibitors in advanced HCC, overall and by viral etiology (HBV, HCV, NBNC).


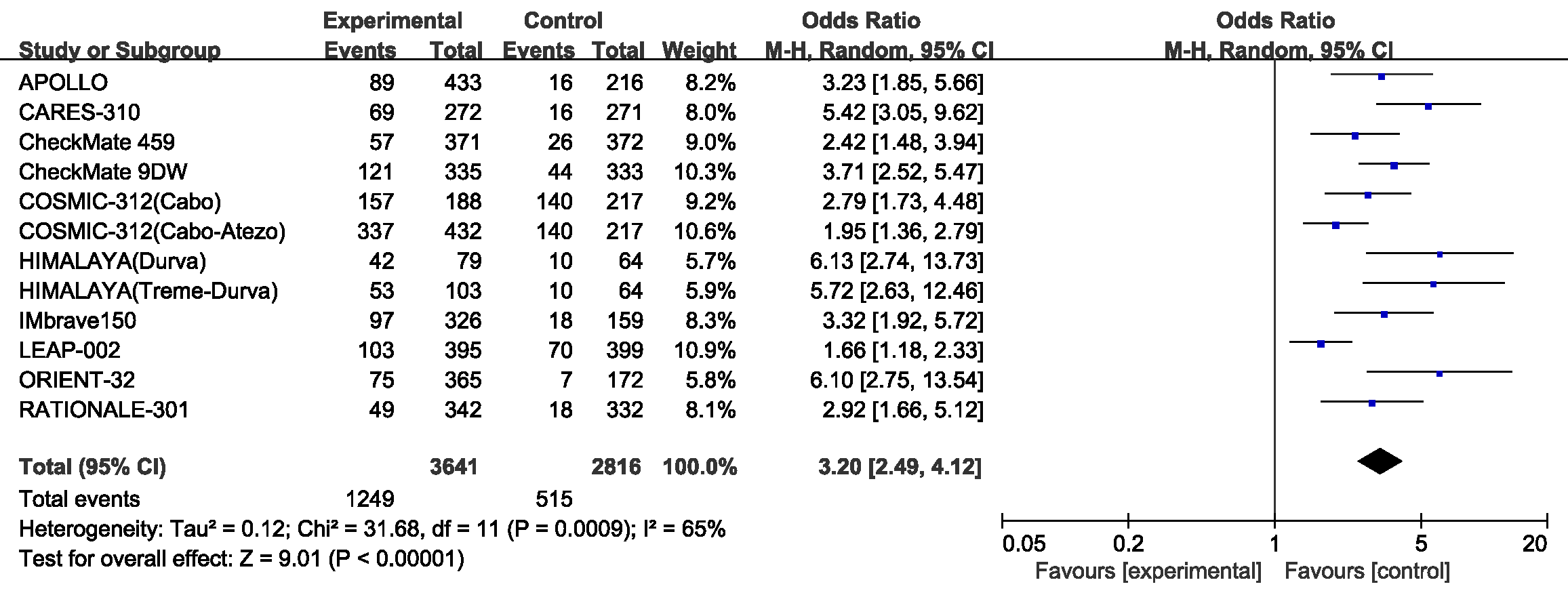


Figure S3. Forest plot for ORR comparing immunotherapy with single-agent TKIs in advanced hepatocellular carcinoma.


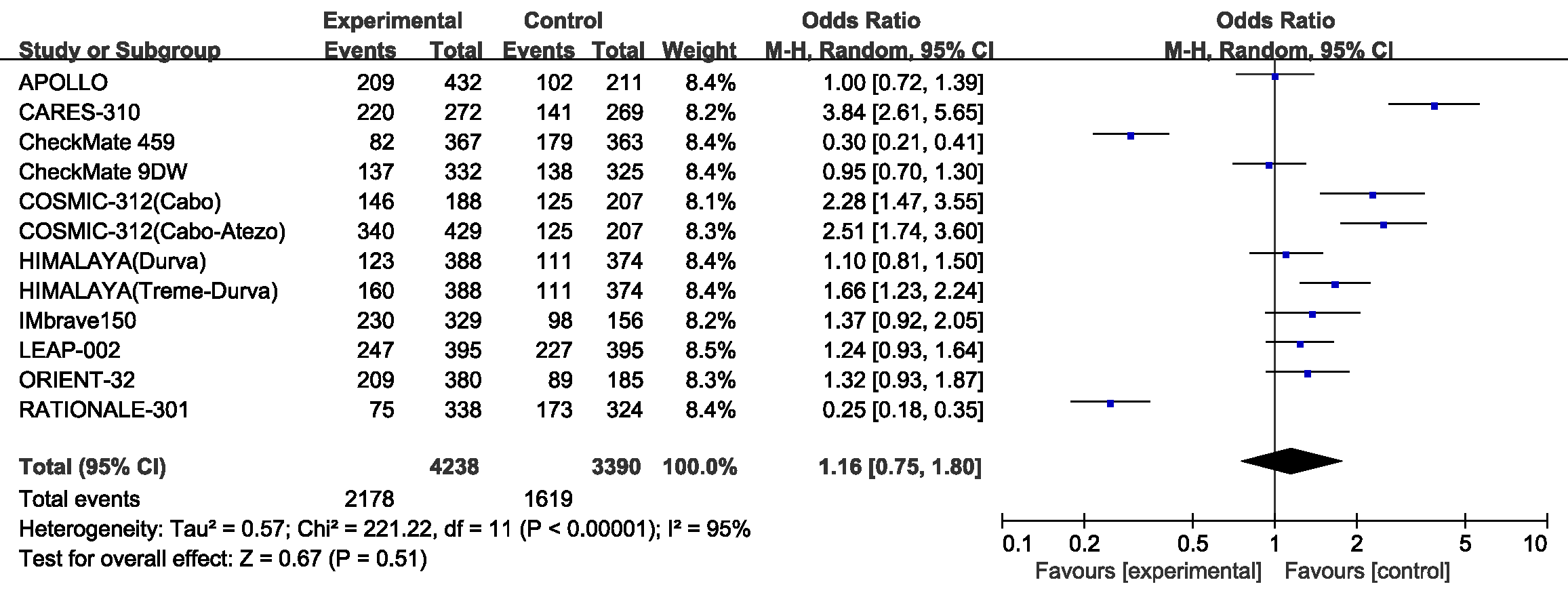


Figure S4. Forest plot for grade ≥3 AEs comparing immunotherapy with single-agent TKIs in advanced hepatocellular carcinoma


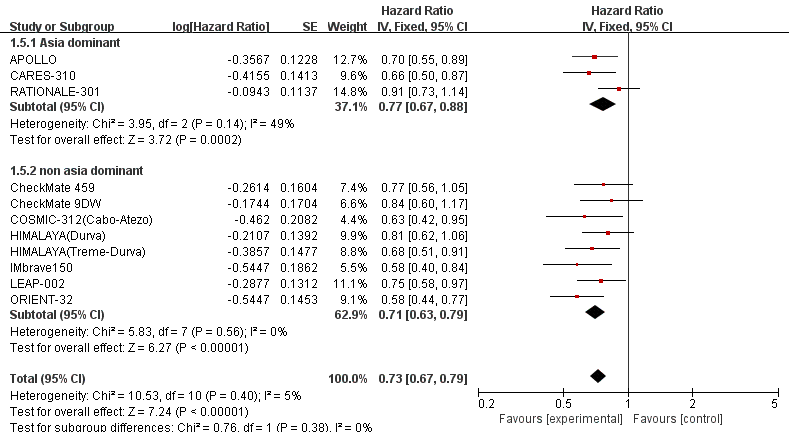


Figure S5. Forest plot of OS for HBV-related HCC: first-line ICI-based regimens versus TKIs in Asia-enriched and non–Asia-enriched trials


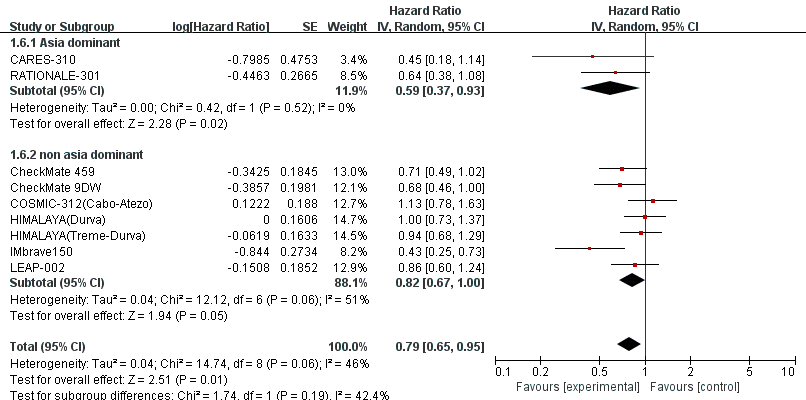


Figure S6. Forest plot of OS for HCV-related HCC: first-line ICI-based regimens versus TKIs in Asia-enriched and non–Asia-enriched trials


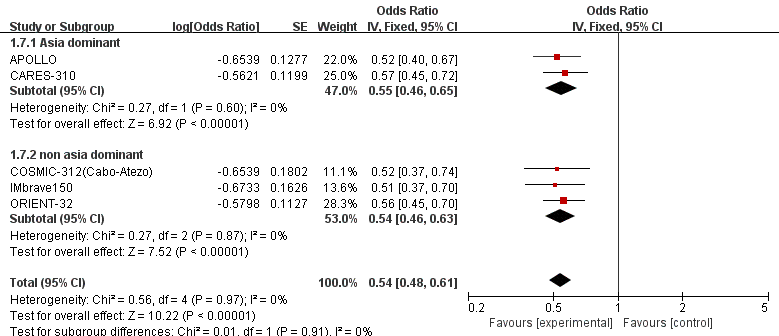


Figure S7. Forest plot of PFS for HBV-related HCC: first-line ICI-based regimens versus TKIs in Asia-enriched and non–Asia-enriched trials


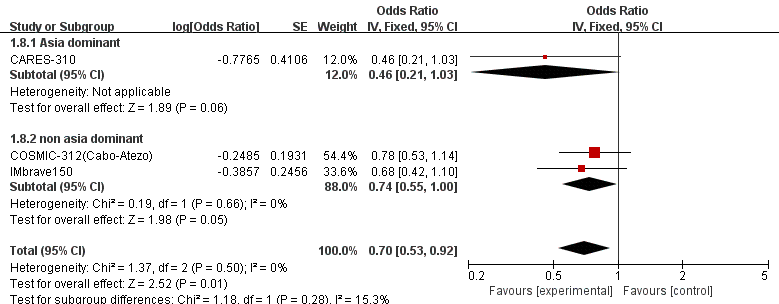


Figure S8. Forest plot of PFS for HCV-related HCC: first-line ICI-based regimens versus TKIs in Asia-enriched and non–Asia-enriched trials
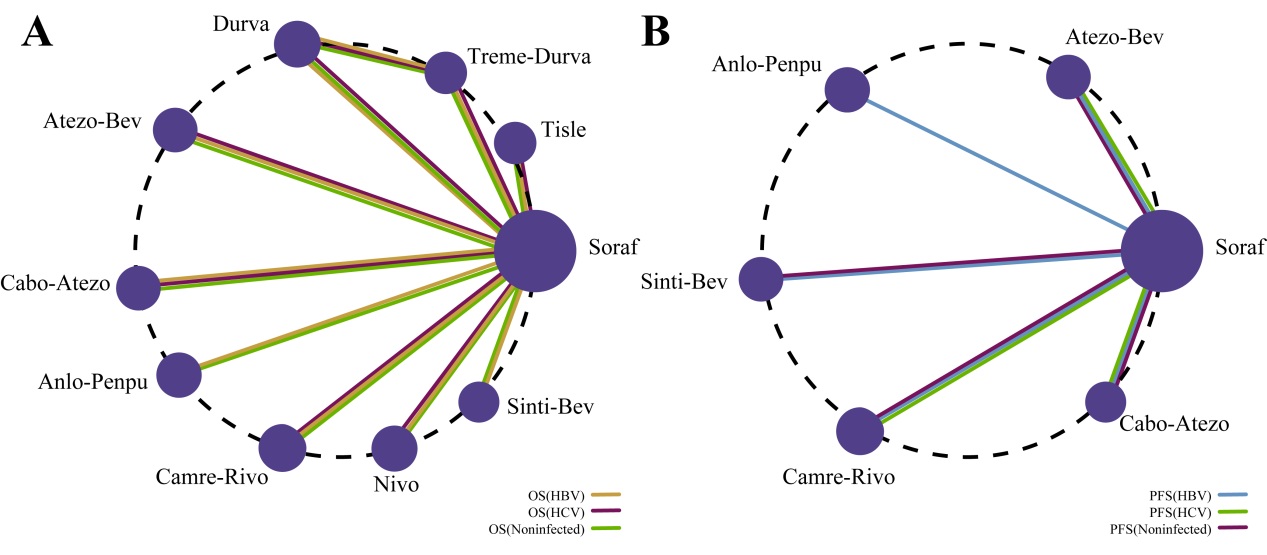


Figure S9. Evidence networks for the comparative efficacy of ICI-based regimens in advanced HCC across HBV, HCV, and NBNC subgroups: (A) OS; (B) PFS.


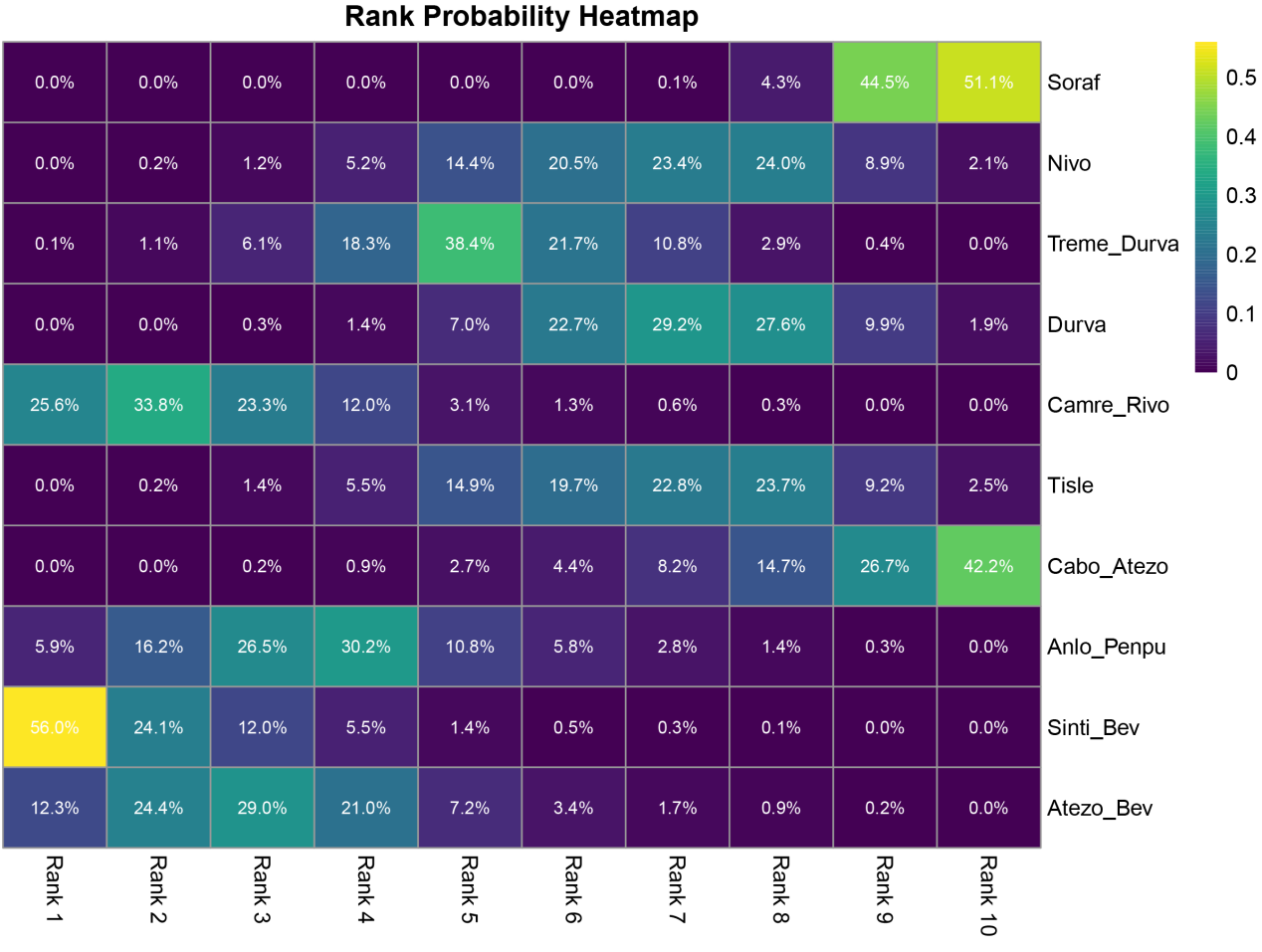


Figure S10.Rank Probability Heatmap of Treatment Regimens for OS in Advanced HCC.


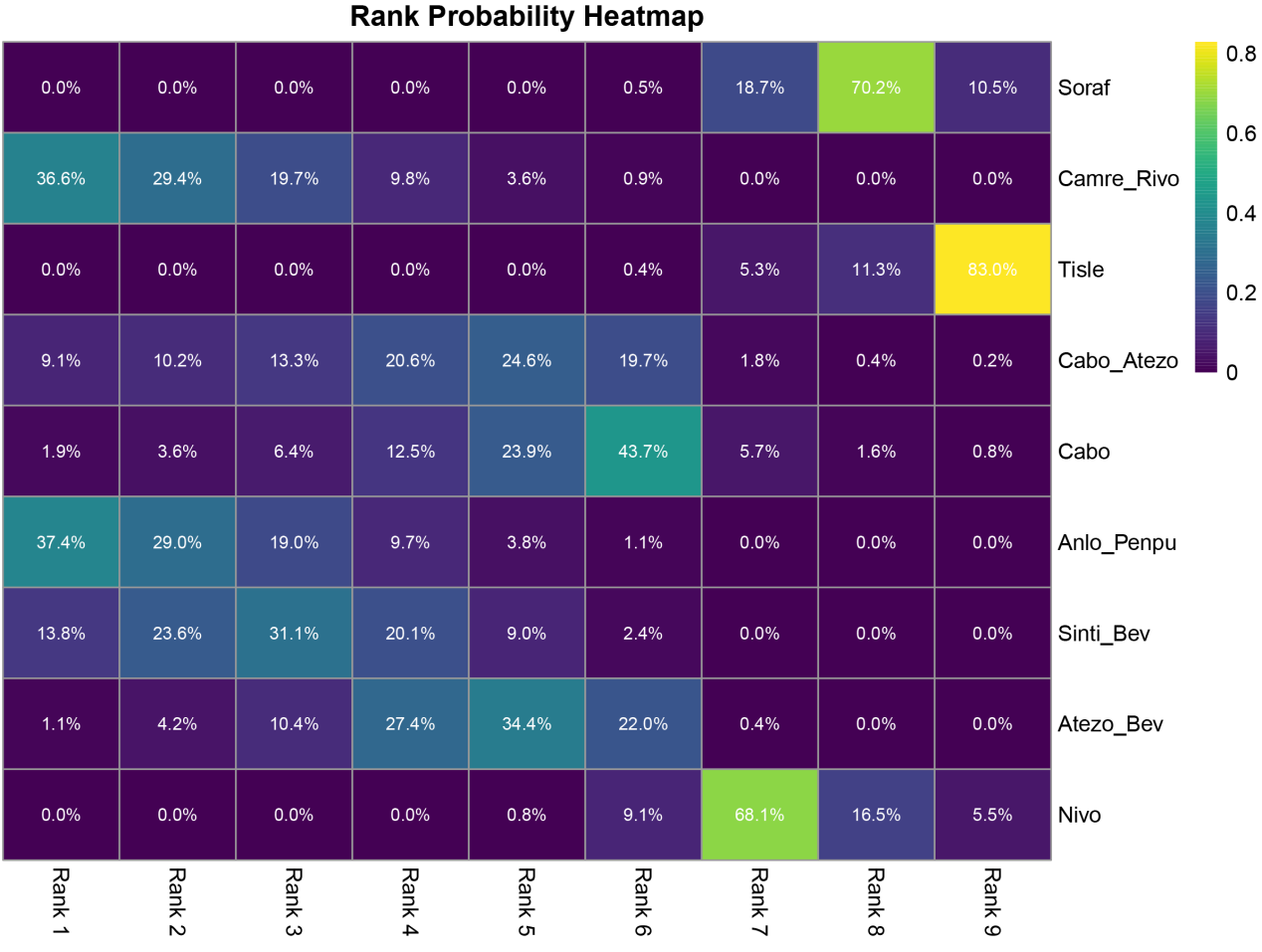


Figure S11.Rank Probability Heatmap of Treatment Regimens for PFS in Advanced HCC.


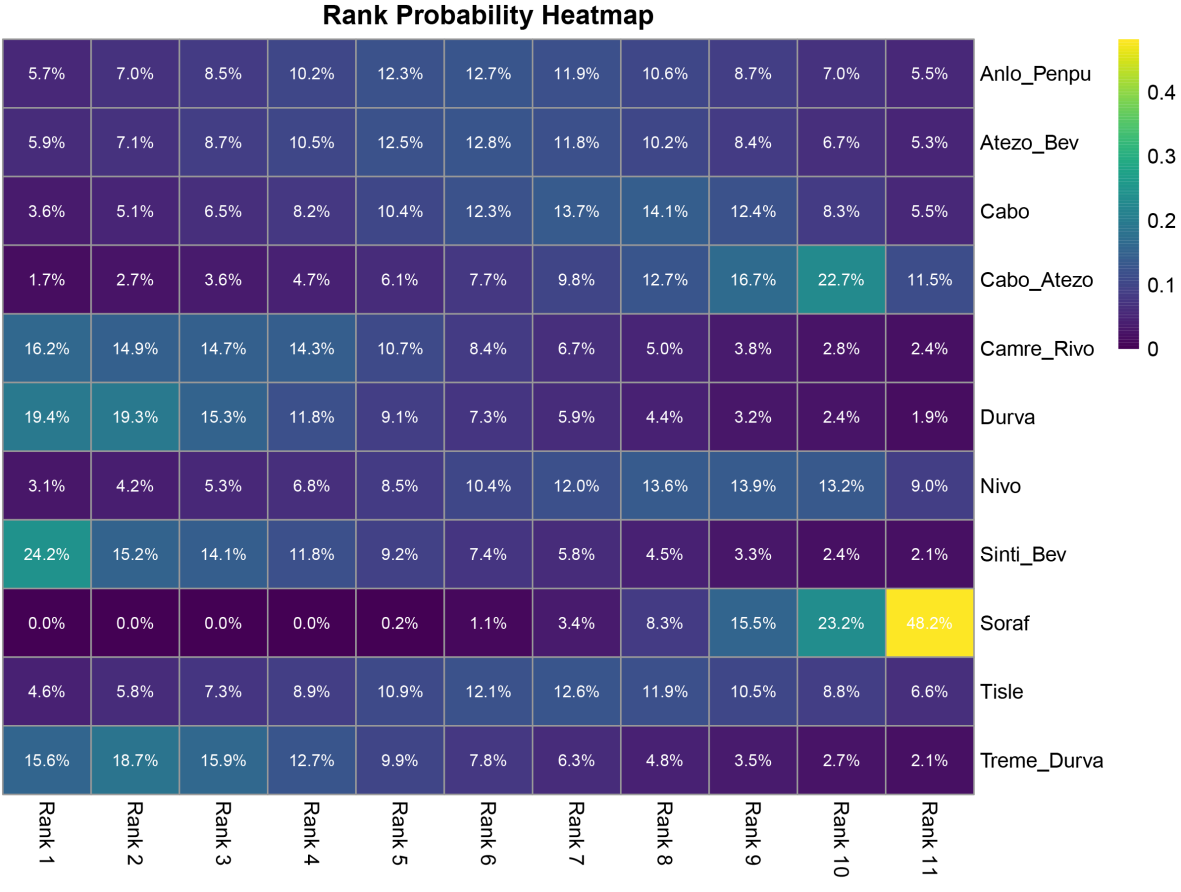


Figure S12.Rank Probability Heatmap of Treatment Regimens for ORR in Advanced HCC.


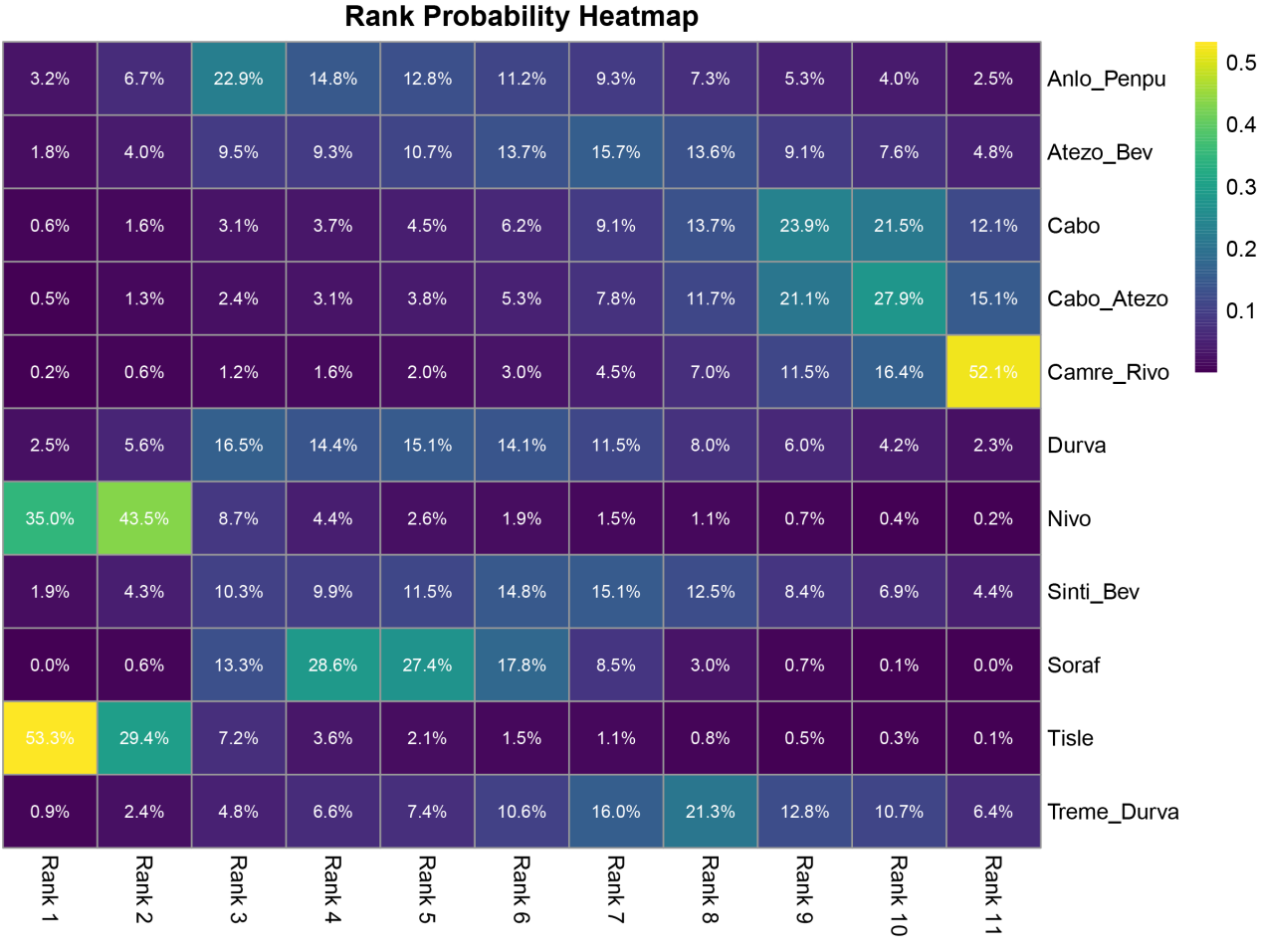


Figure S13.Rank Probability Heatmap of Treatment Regimens for AE≥3 in Advanced HCC.


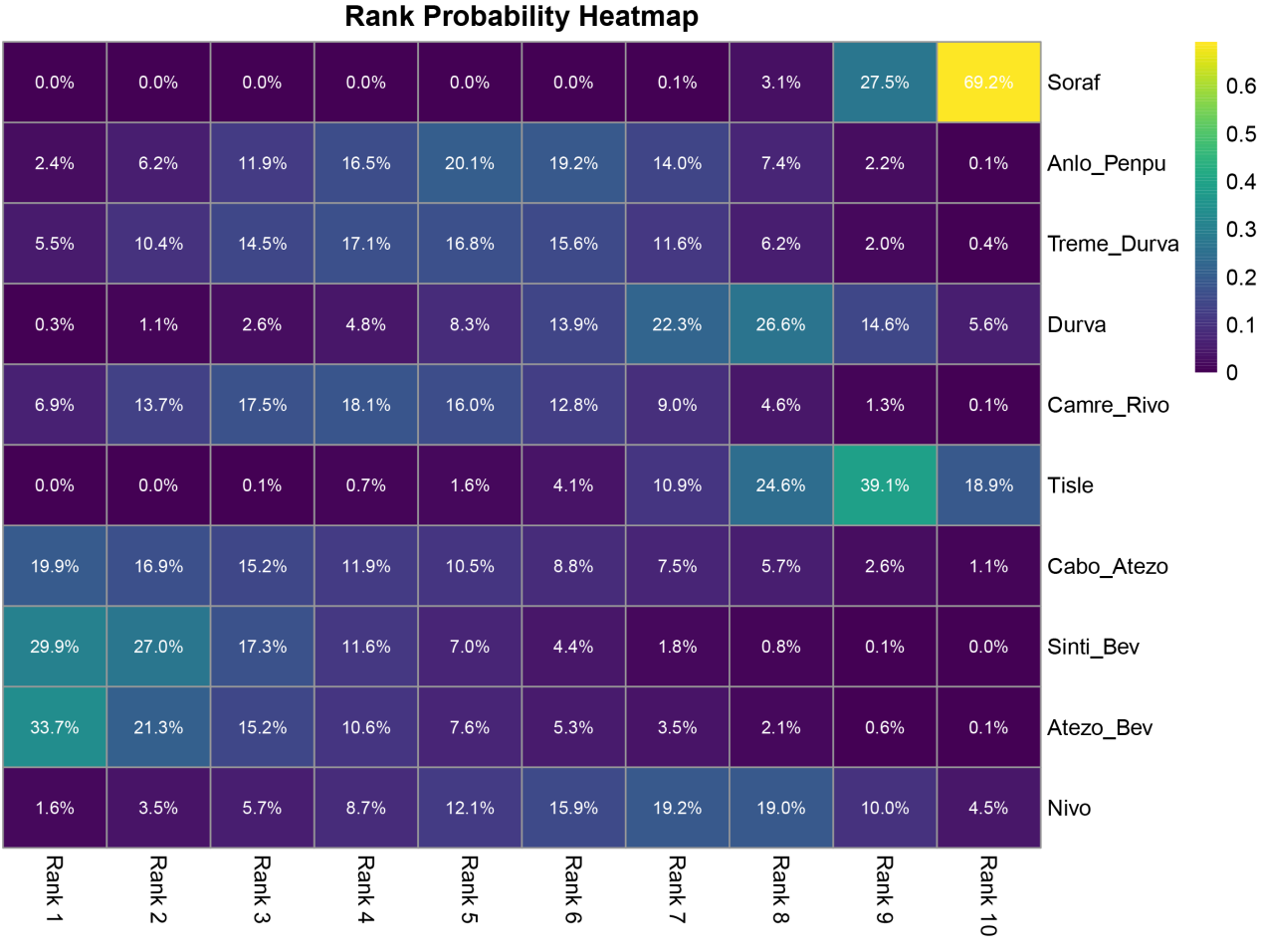


Figure S14.Rank Probability Heatmap of Treatment Regimens for OS in HBV-positive Advanced HCC.


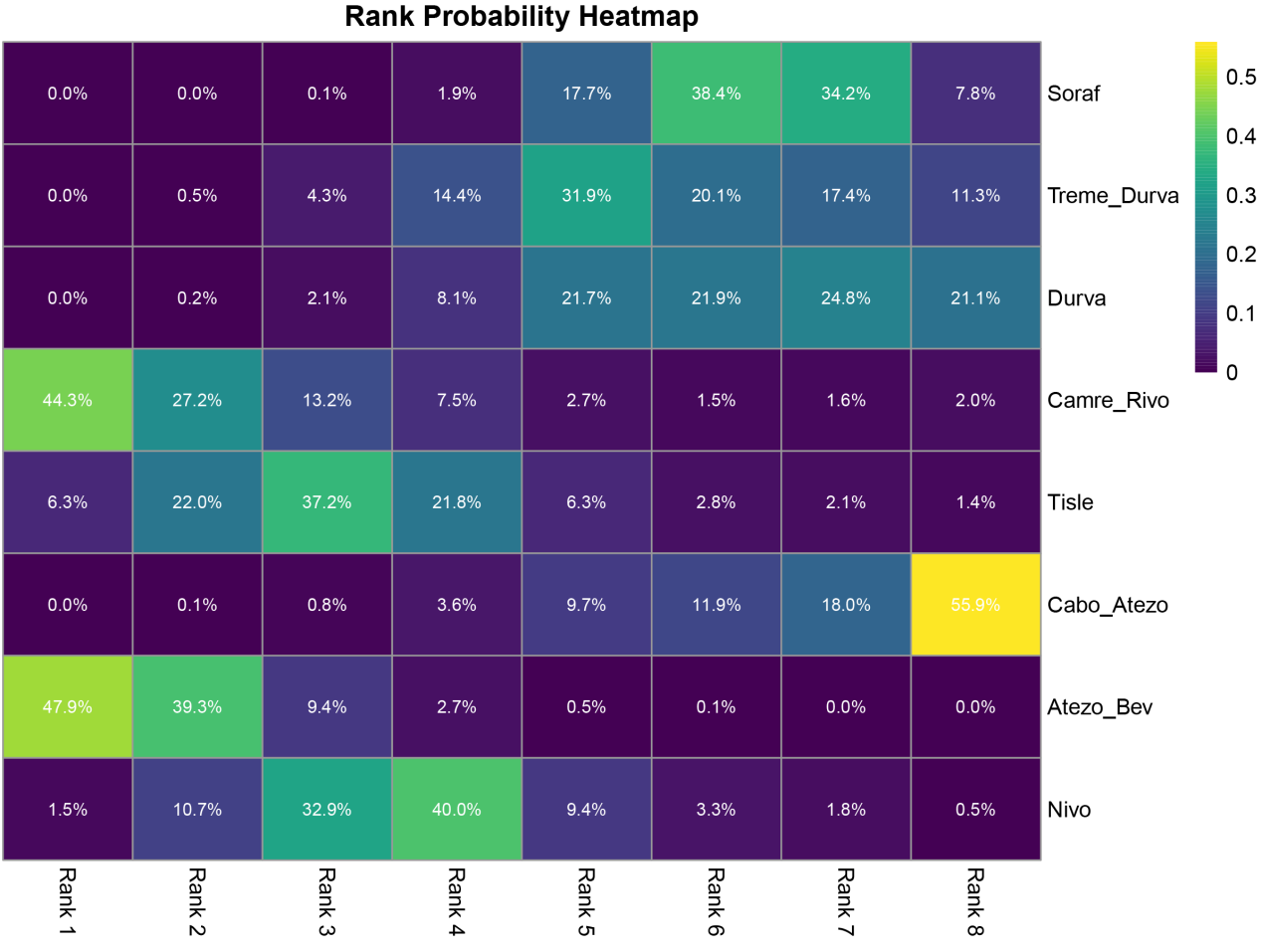


Figure S15.Rank Probability Heatmap of Treatment Regimens for OS in HCV-positive Advanced HCC.


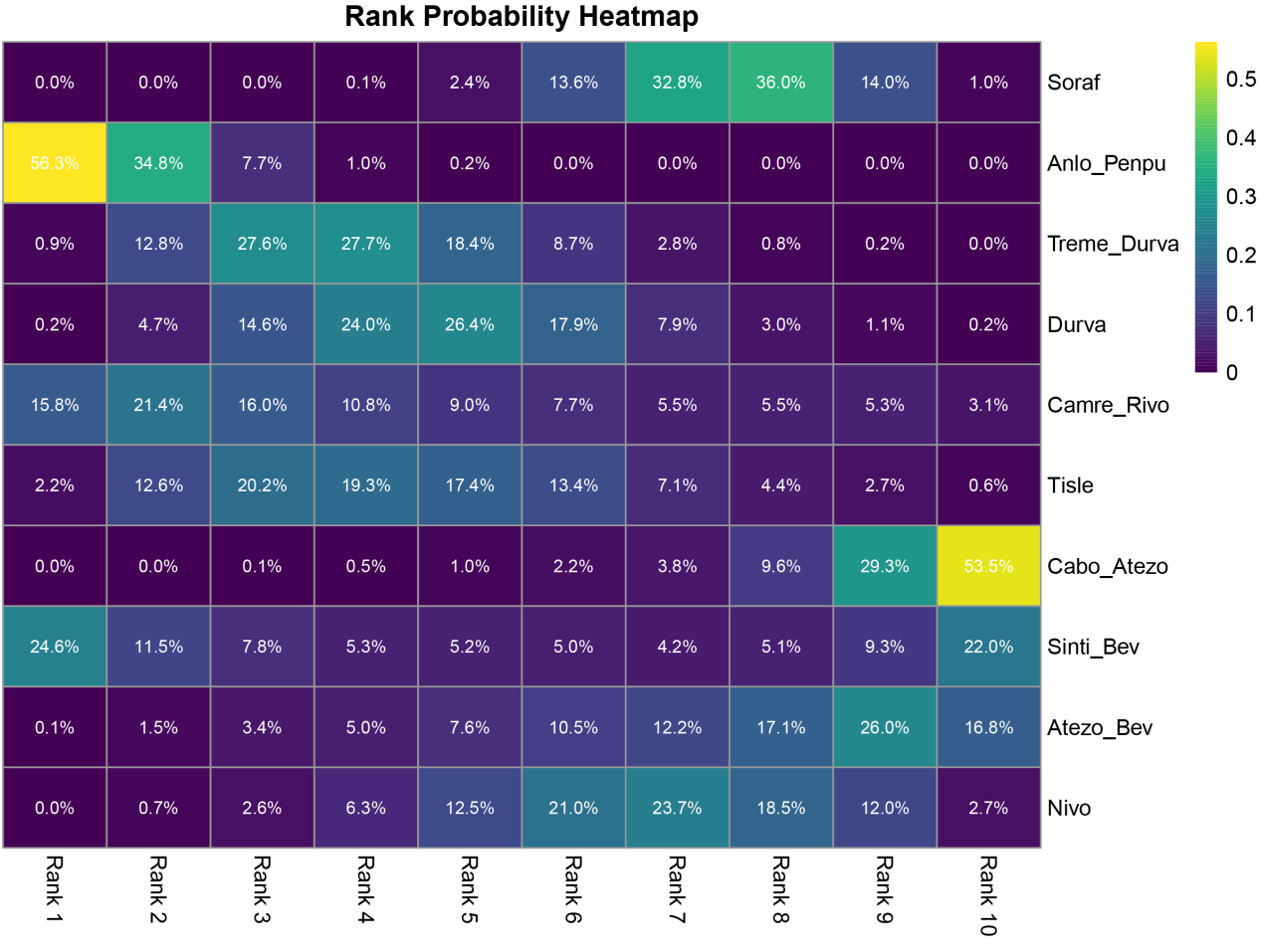


Figure S16.Rank Probability Heatmap of Treatment Regimens for OS in NBNCAdvanced HCC.


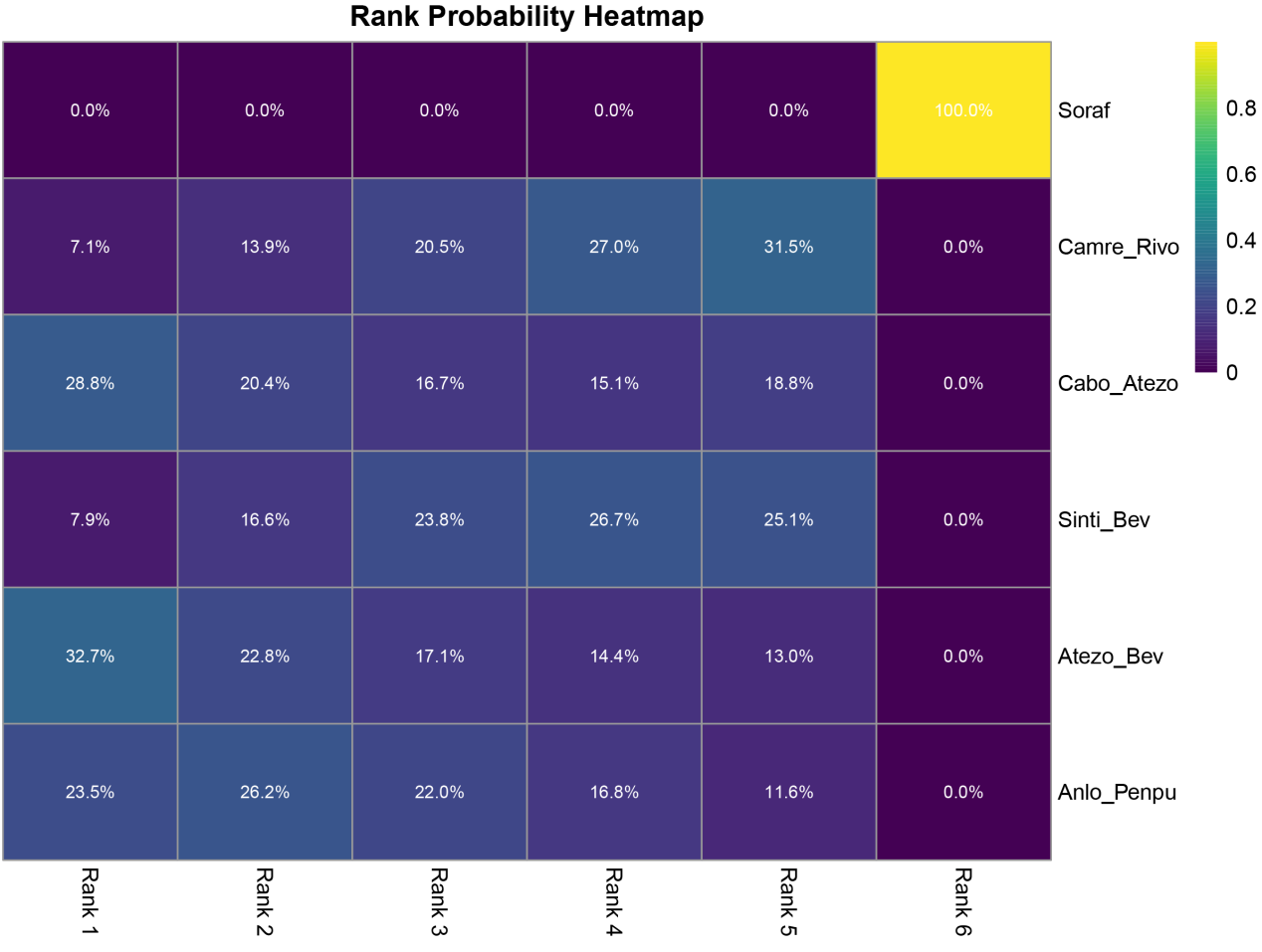


Figure S17.Rank Probability Heatmap of Treatment Regimens for PFS in HBV-positive Advanced HCC.


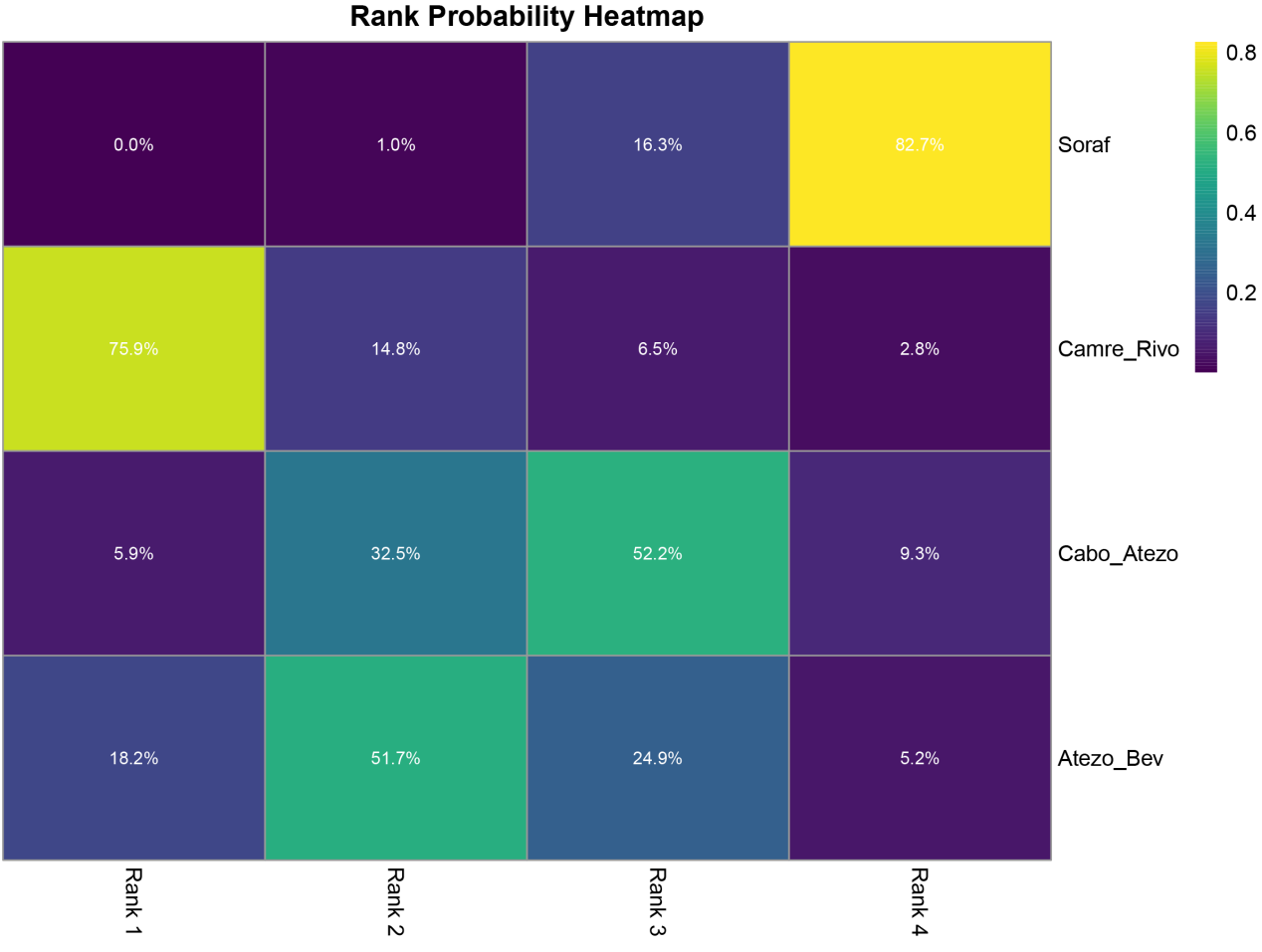


Figure S18.Rank Probability Heatmap of Treatment Regimens for PFS in HCV-positive Advanced HCC.


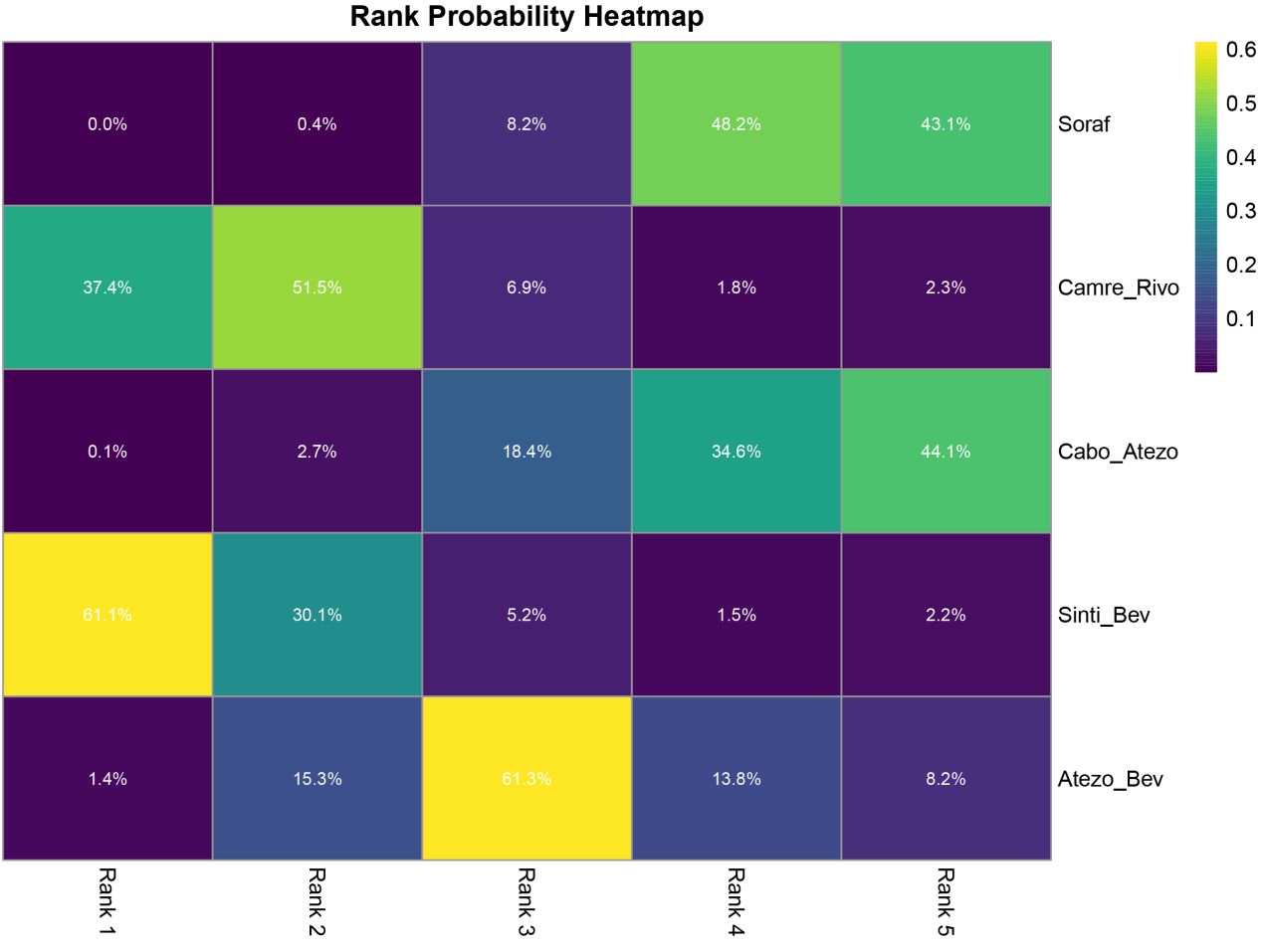


Figure S19.Rank Probability Heatmap of Treatment Regimens for PFS in NBNC Advanced HCC.

Figure S20.League tables from a Bayesian random-effects network meta-analysis comparing first-line immunotherapy regimens in advanced hepatocellular carcinoma.

(A) Lower triangle (yellow): OS—HRs with 95% CIs; upper triangle (blue): PFS—HRs with 95% CIs. For both OS and PFS, HR < 1.00 favors the row regimen, indicating greater survival benefit.

(B) Lower triangle (yellow): ORR—ORs with 95% CIs, where OR > 1.00 favors the row regimen; upper triangle (blue): grade ≥3 AEs—ORs with 95% CIs, where OR < 1.00 indicates fewer severe adverse events and thus better safety for the row regimen.


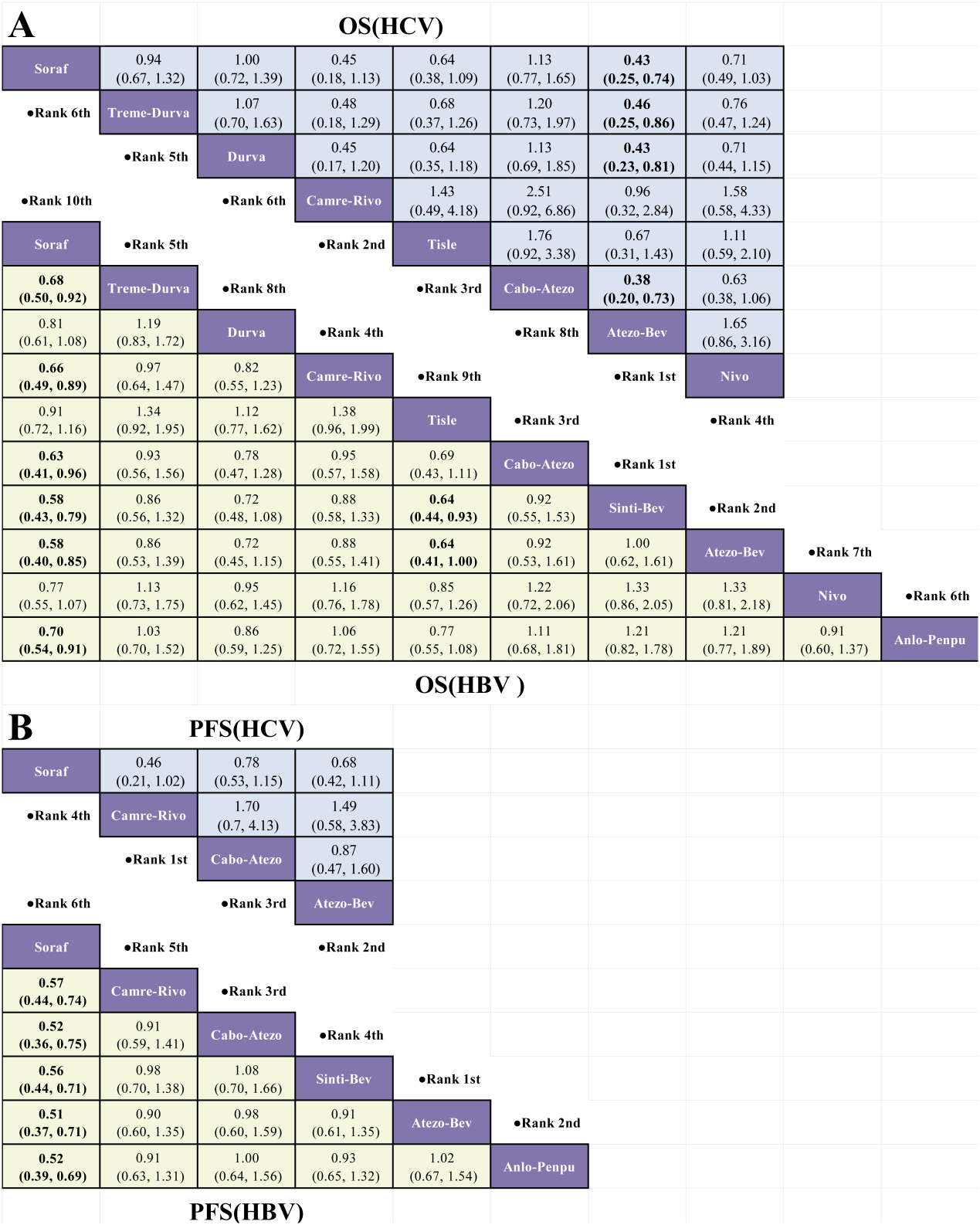


Figure S21. League tables from a Bayesian random-effects network meta-analysis comparing first-line immunotherapy regimens in HBV- and HCV-positive advanced hepatocellular carcinoma.

(A) OS: lower triangle (yellow) = HBV, upper triangle (blue) = HCV; effects are presented as HRs with 95% CIs; HR < 1.00 favors the row regimen.

(B) PFS: lower triangle (yellow) = HBV, upper triangle (blue) = HCV; effects are presented as HRs with 95% CIs; HR < 1.00 favors the row regimen.


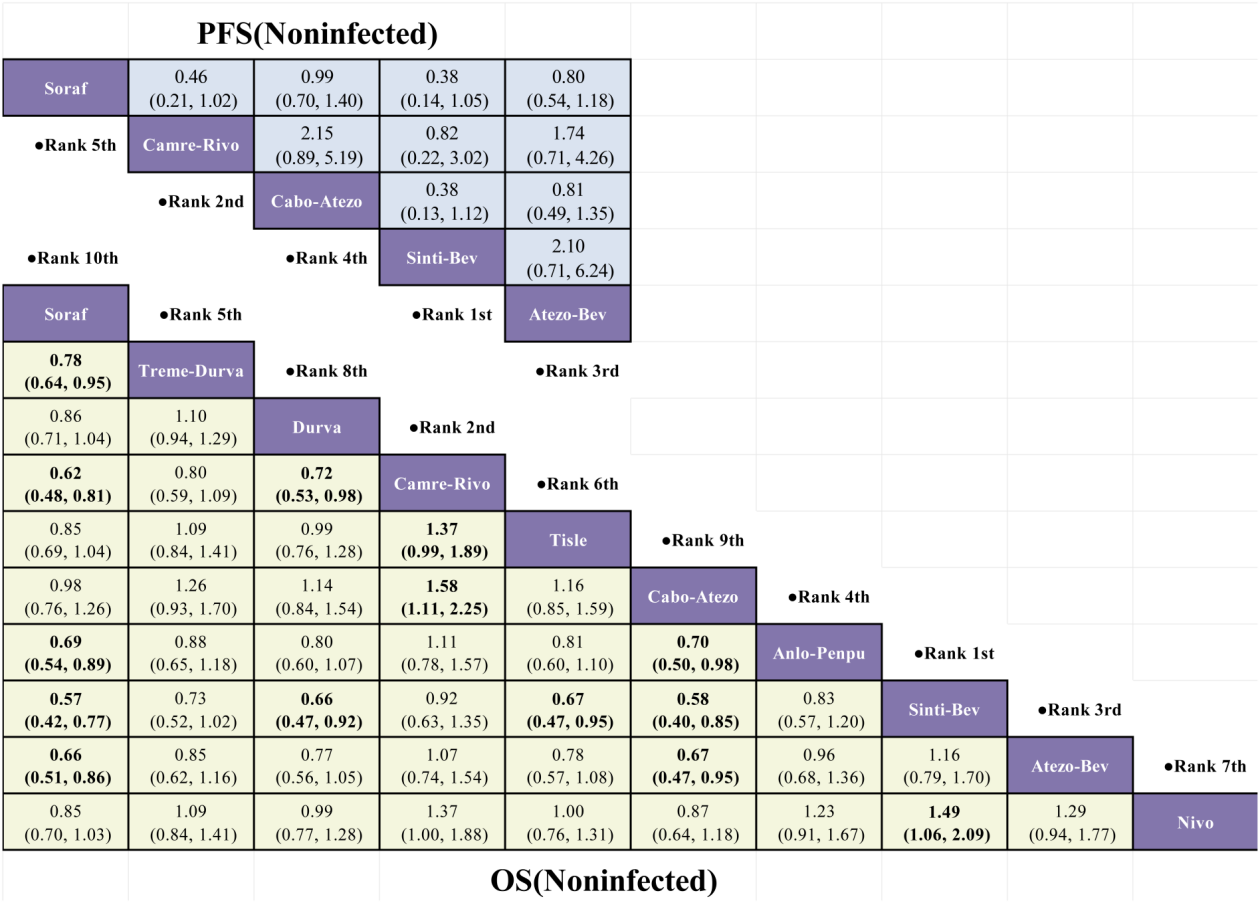


Figure S22. League table of comparative efficacy from a random-effects network meta-analysis of immunotherapy regimens in NBNC advanced hepatocellular carcinoma


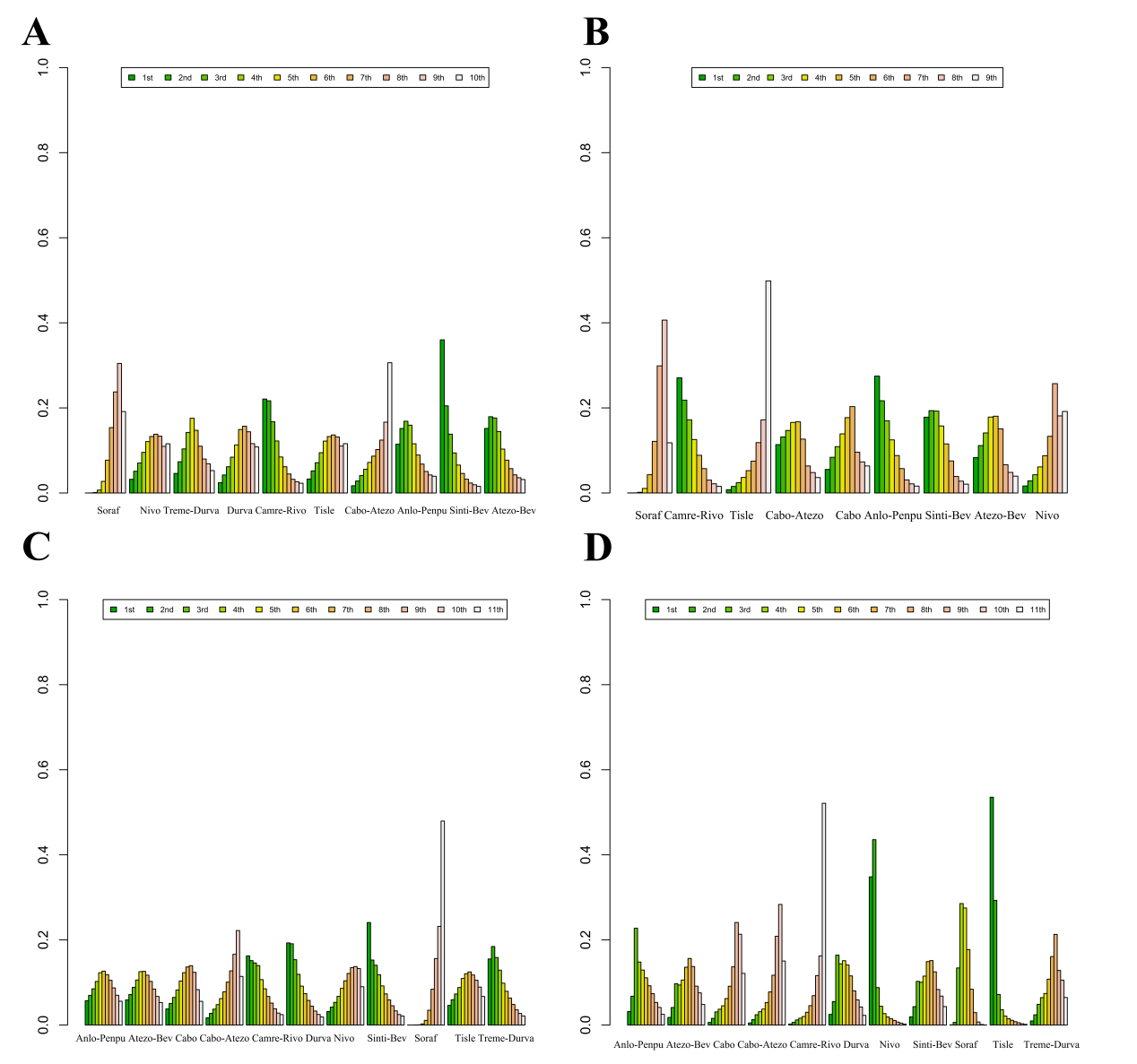


Figure S23.Bayesian random-effects rank-probability profiles for the comparative efficacy and safety of first-line immunotherapy in advanced hepatocellular carcinoma: (A) OS; (B) PFS; (C) ORR; (D) grade ≥3 adverse events.


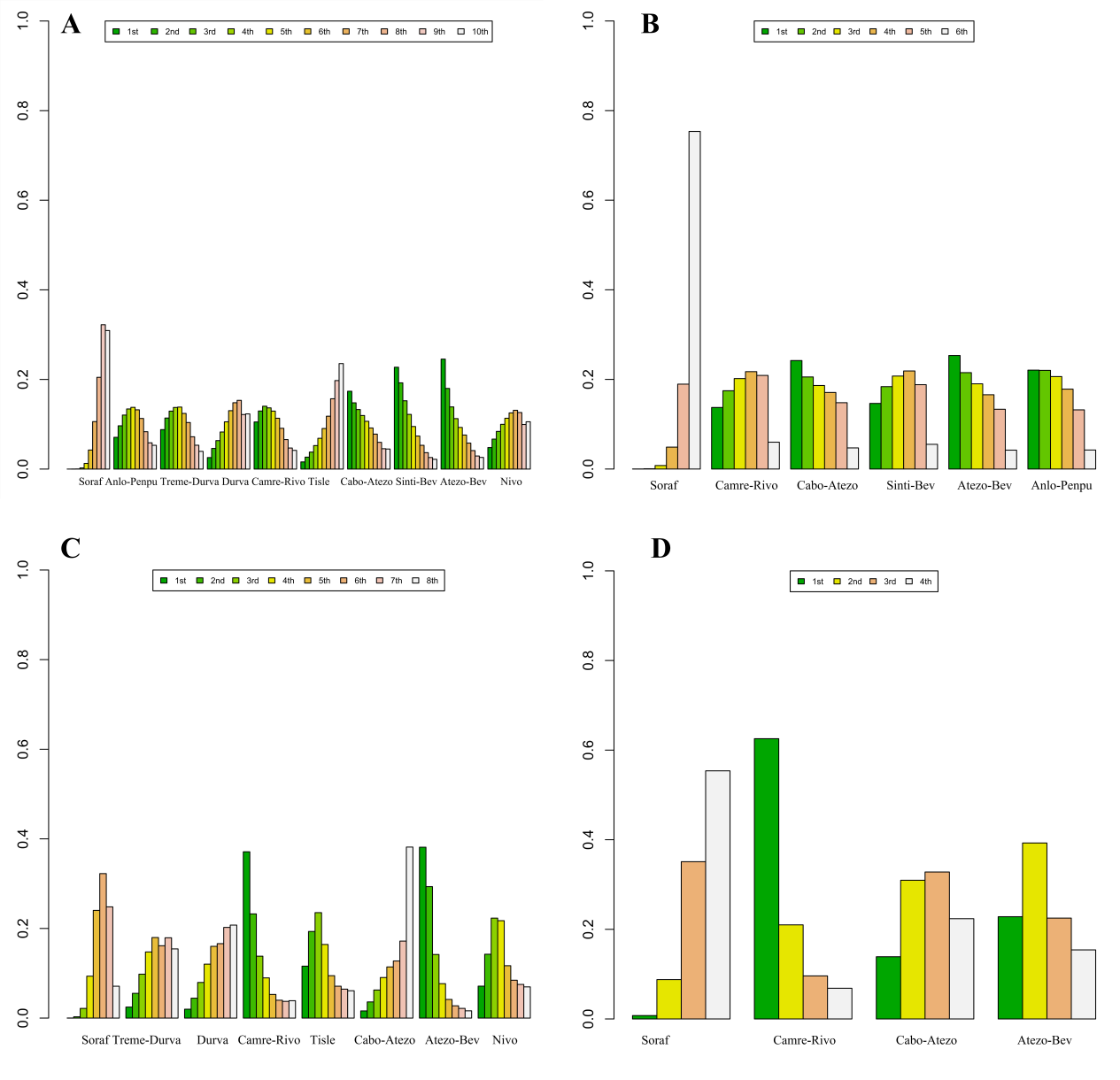


Figure S24.Rank-probability profiles from a Bayesian random-effects network meta-analysis for the efficacy of first-line immunotherapy regimens in HBV- and HCV-positive advanced hepatocellular carcinoma: (A) HBV—OS; (B) HBV—PFS; (C) HCV—OS; (D) HCV—PFS


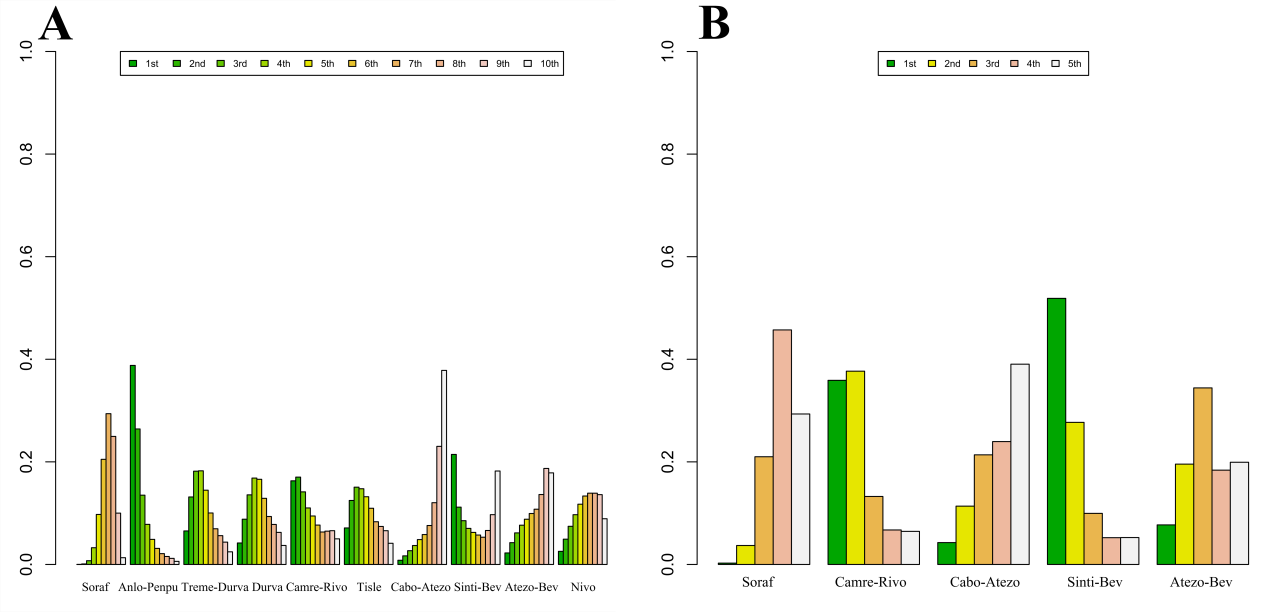


Figure S25.Rank-probability profiles from a Bayesian random-effects network meta-analysis for the efficacy of first-line immunotherapy regimens in NBNC advanced hepatocellular carcinoma: (A) OS; (B) PFS.


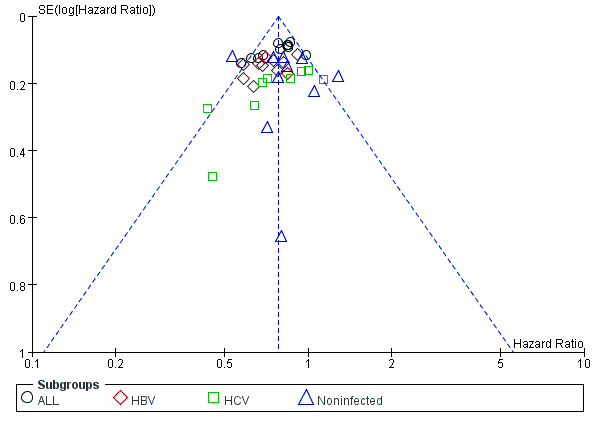


Figure S26.Funnel plot for OS in advanced hepatocellular carcinoma: immunotherapy vs tyrosine kinase inhibitors.


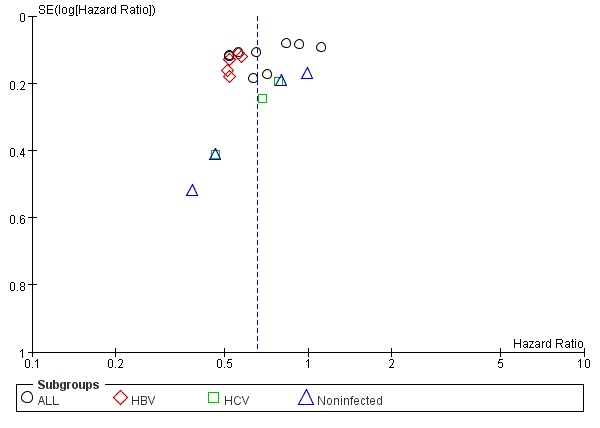


Figure S27.Funnel plot for PFS in advanced hepatocellular carcinoma: immunotherapy vs tyrosine kinase inhibitors.


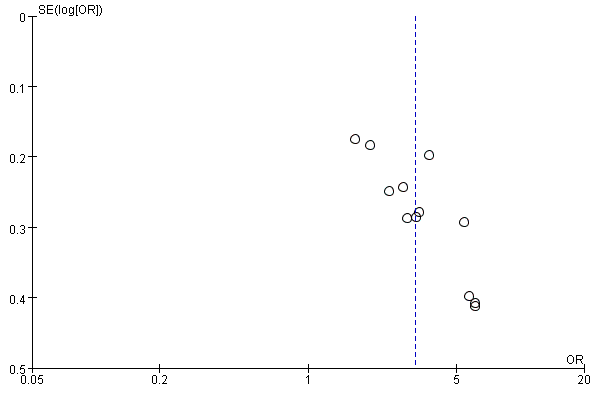


Figure S28.Funnel plot for ORR in advanced hepatocellular carcinoma: immunotherapy vs tyrosine kinase inhibitors.


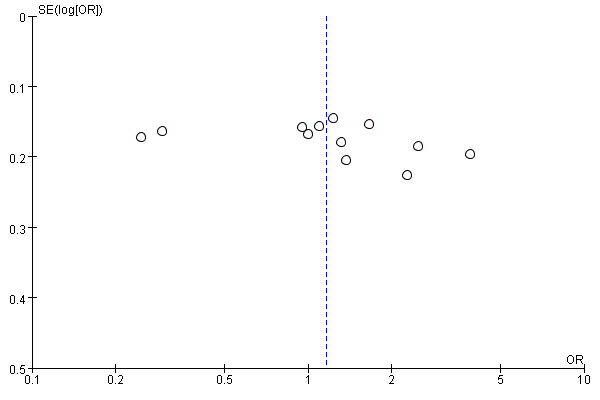


Figure S29.Funnel plot for AEs≥3 in advanced hepatocellular carcinoma: immunotherapy vs tyrosine kinase inhibitors.


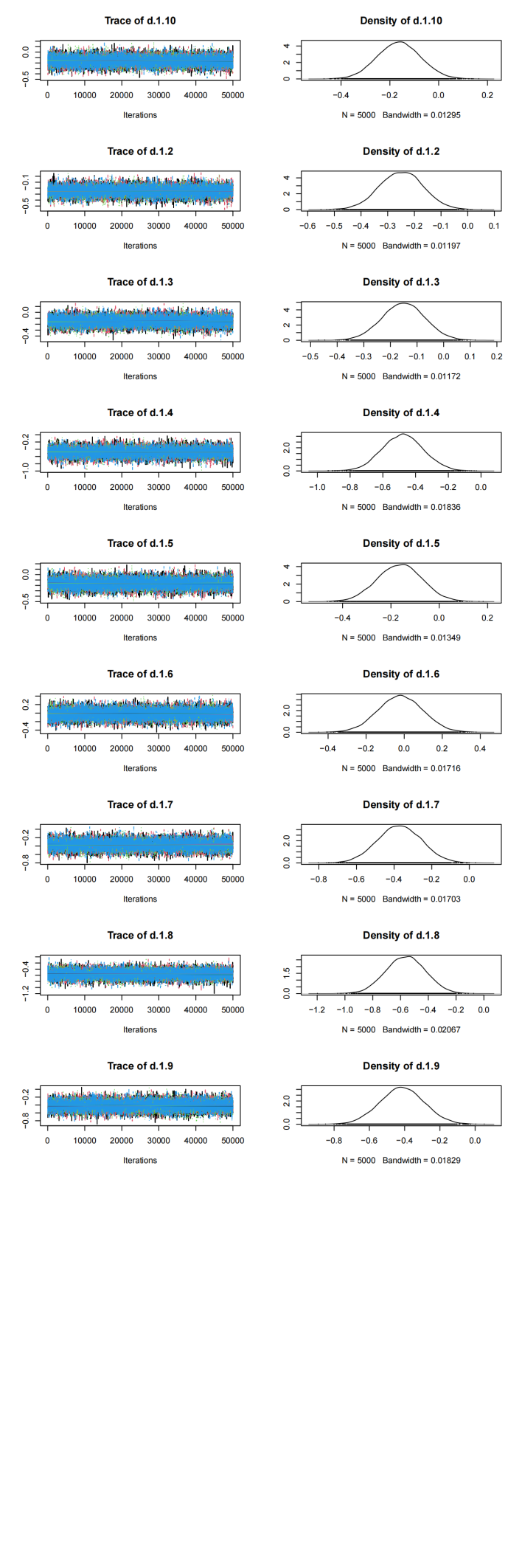


Figure S30.MCMC trace and posterior density plots for OS in advanced hepatocellular carcinoma: immunotherapy vs tyrosine kinase inhibitors.


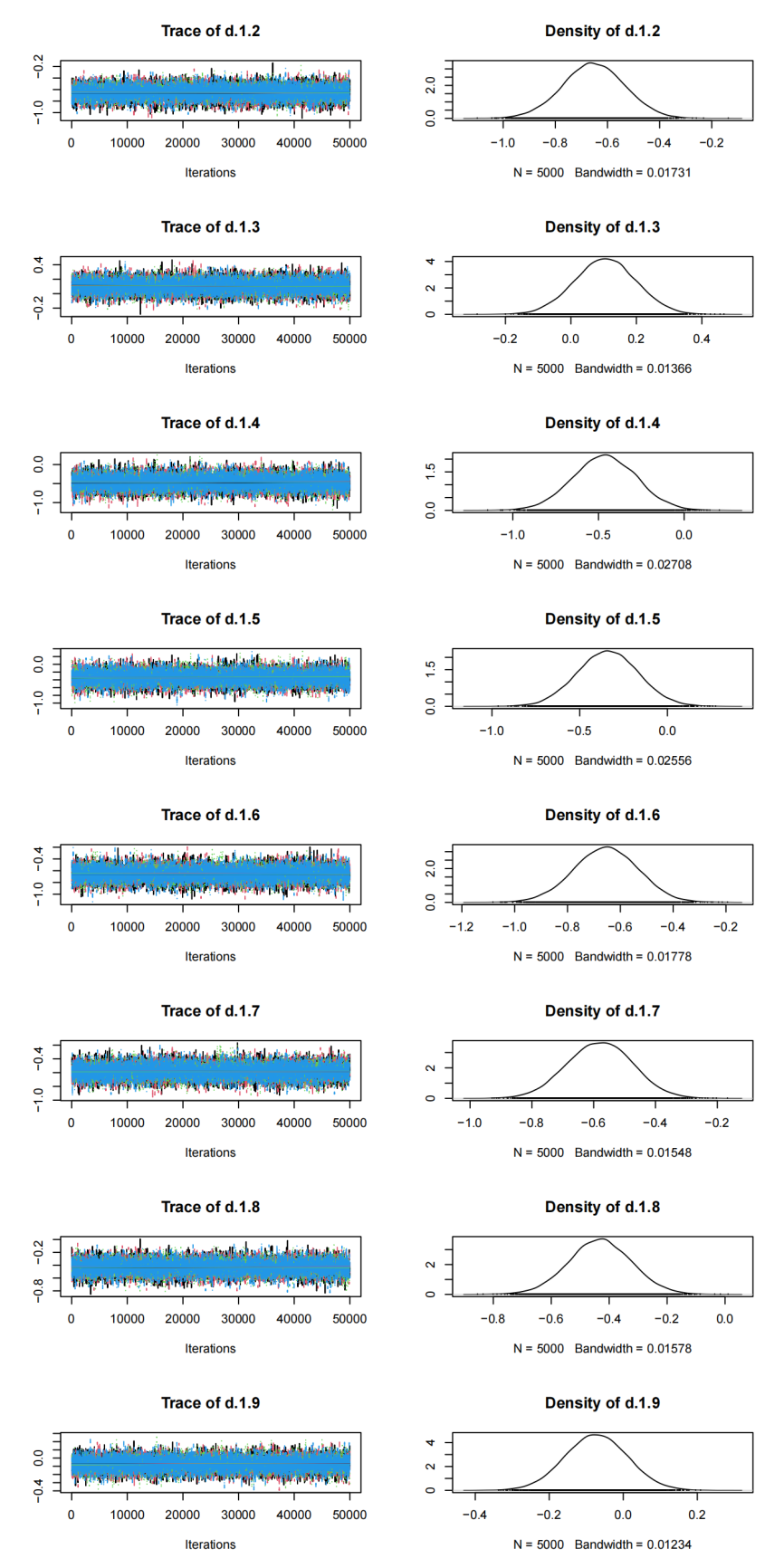


Figure S31.MCMC trace and posterior density plots for PFS in advanced hepatocellular carcinoma: immunotherapy vs tyrosine kinase inhibitors.


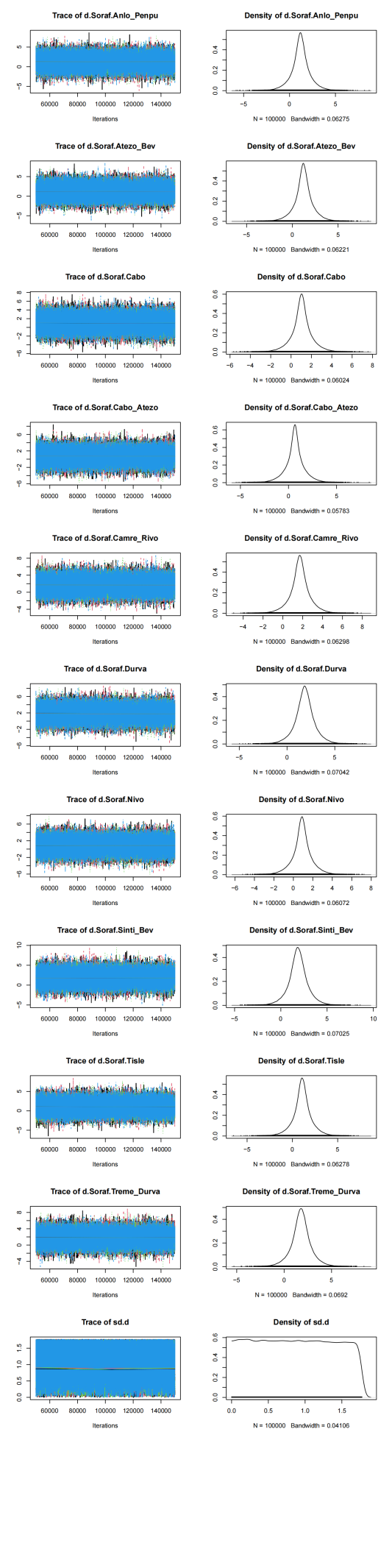


Figure S32.MCMC trace and posterior density plots for ORR in advanced hepatocellular carcinoma: immunotherapy vs tyrosine kinase inhibitors.


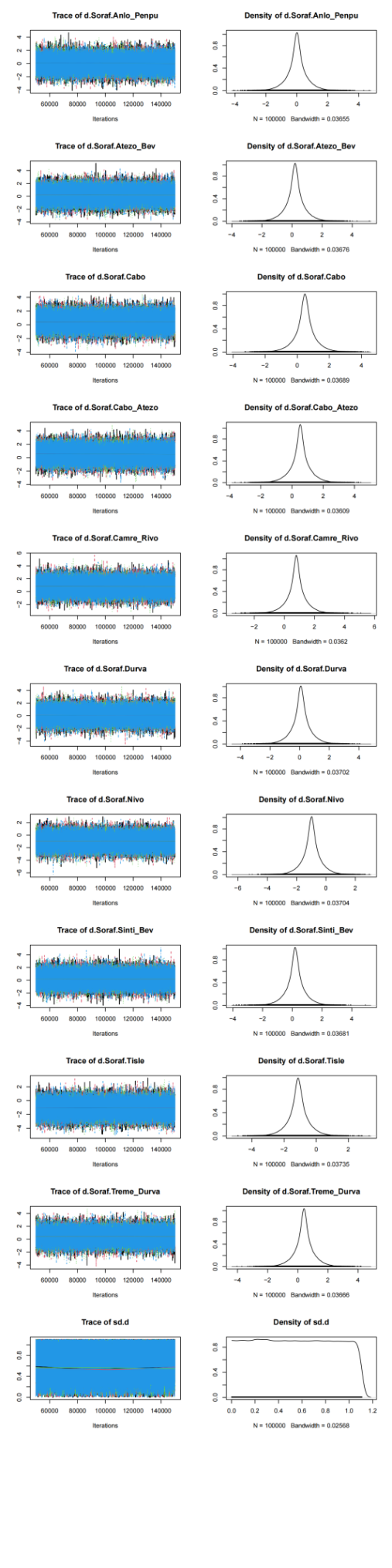


Figure S33.MCMC trace and posterior density plots for AEs≥3 in advanced hepatocellular carcinoma: immunotherapy vs tyrosine kinase inhibitors.


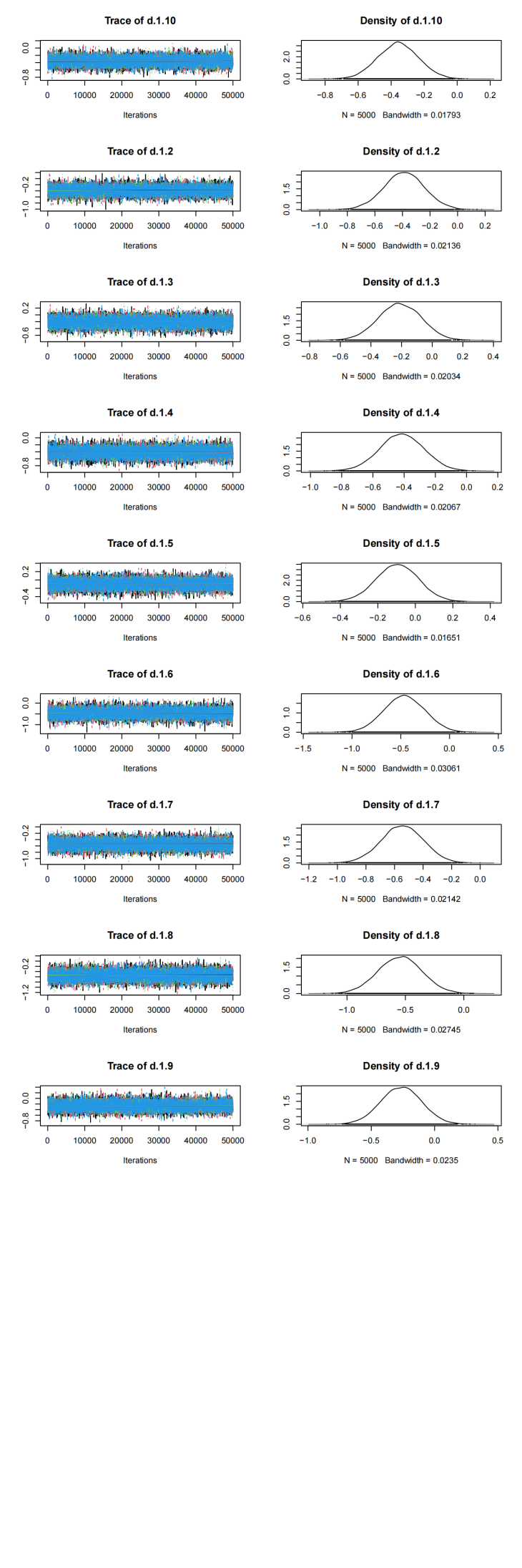


Figure S34.MCMC trace and posterior density plots for OS in HBV-positive advanced hepatocellular carcinoma: immunotherapy vs tyrosine kinase inhibitors.


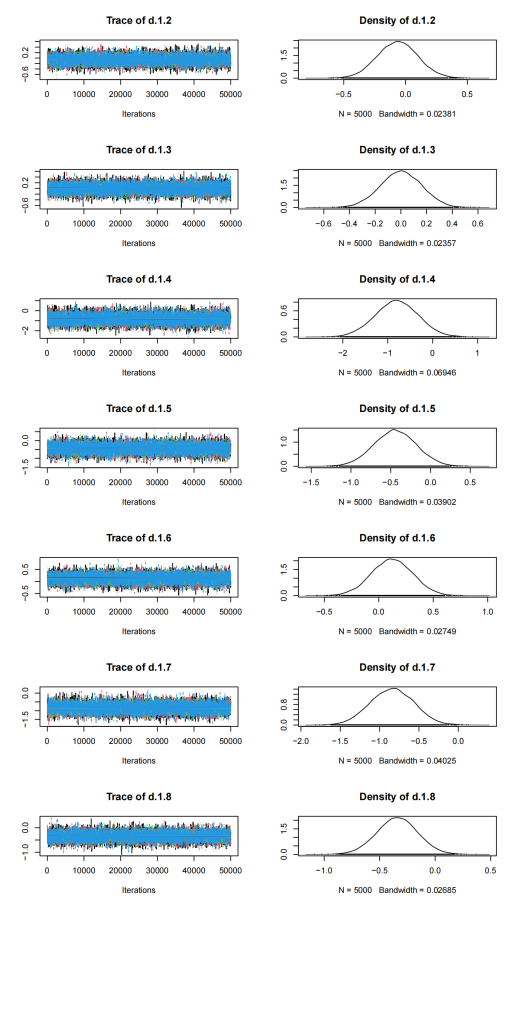


Figure S35.MCMC trace and posterior density plots for OS in HCV-positive advanced hepatocellular carcinoma: immunotherapy vs tyrosine kinase inhibitors.


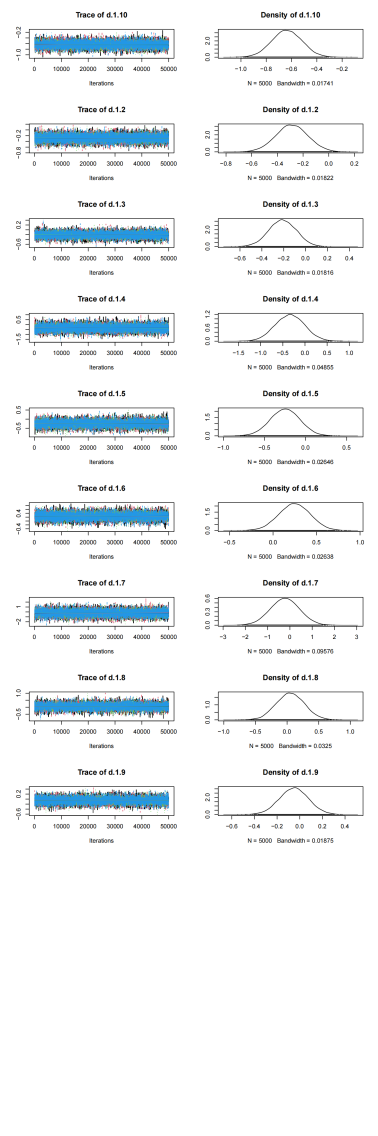


Figure S36.MCMC trace and posterior density plots for OS in NBNC advanced hepatocellular carcinoma: immunotherapy vs tyrosine kinase inhibitors.


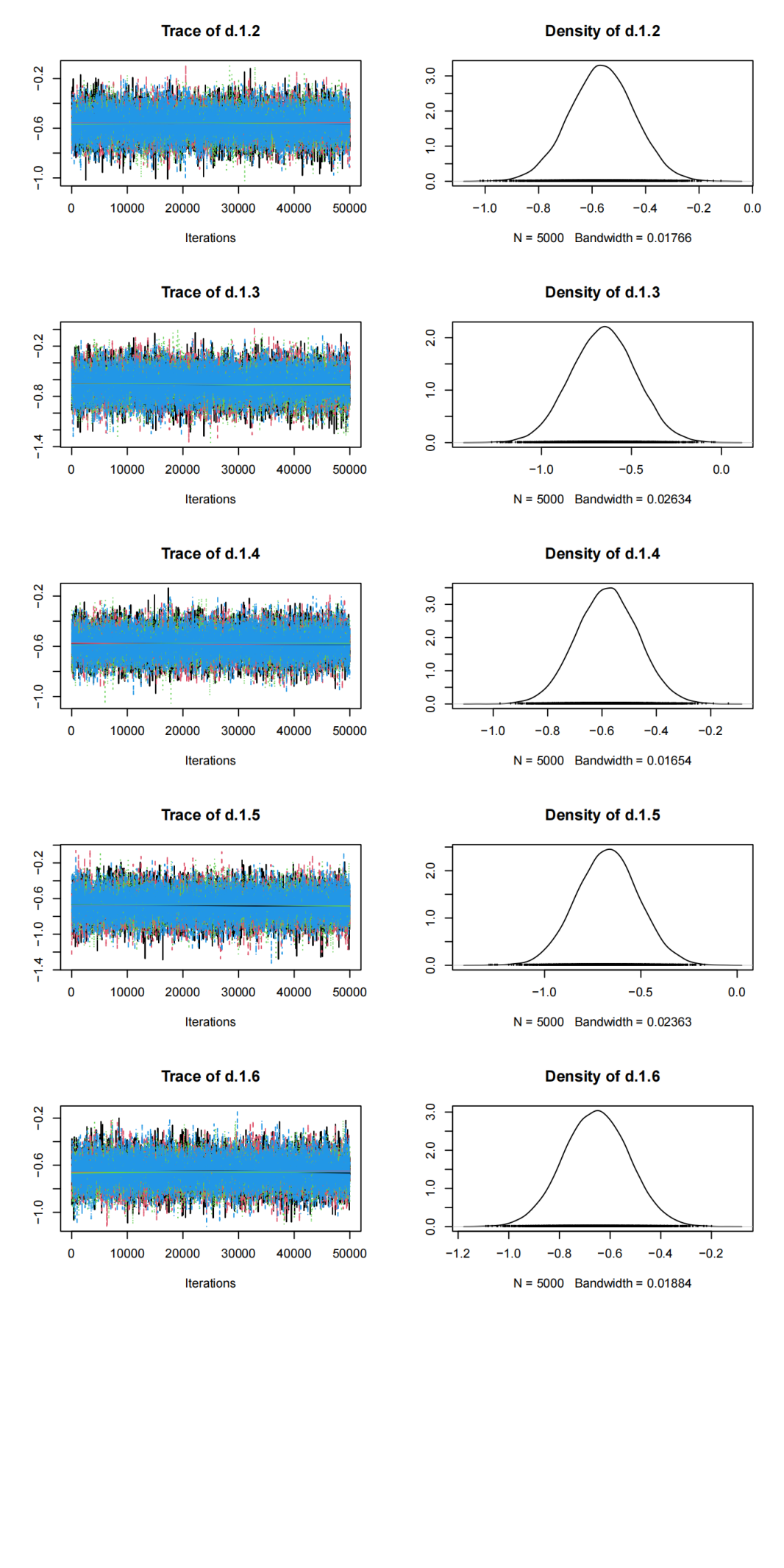


Figure S37.MCMC trace and posterior density plots for PFS in HBV-positive advanced hepatocellular carcinoma: immunotherapy vs tyrosine kinase inhibitors.


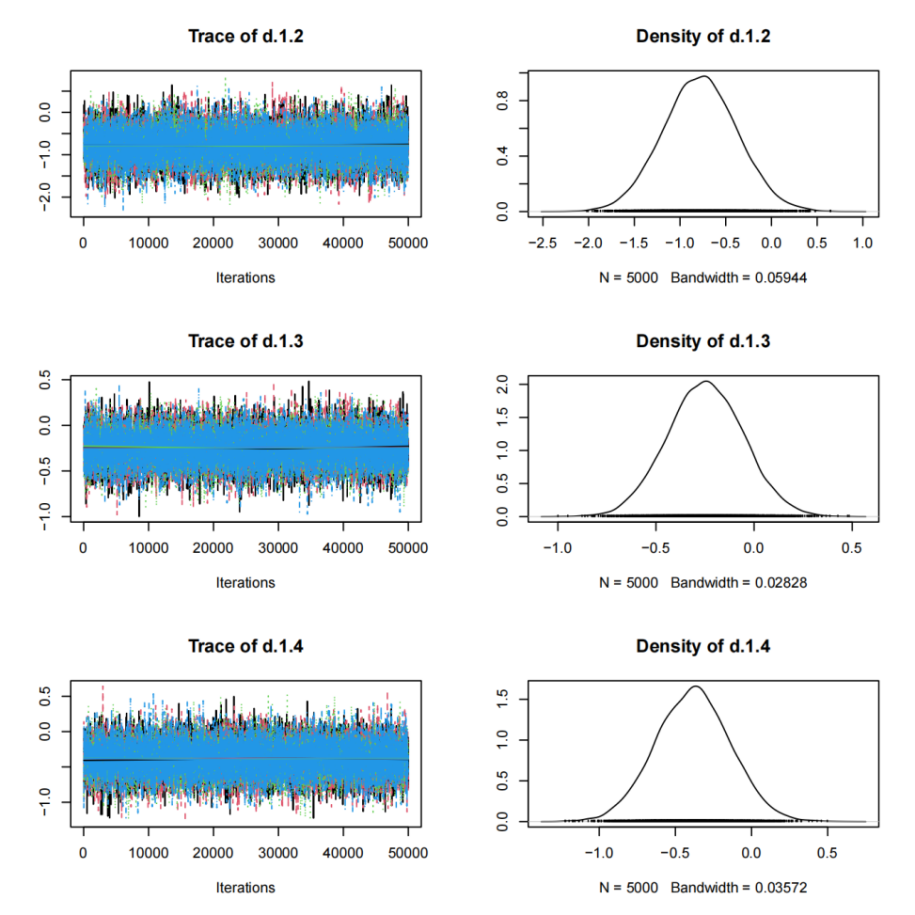


Figure S38.MCMC trace and posterior density plots for PFS in HCV-positive advanced hepatocellular carcinoma: immunotherapy vs tyrosine kinase inhibitors.


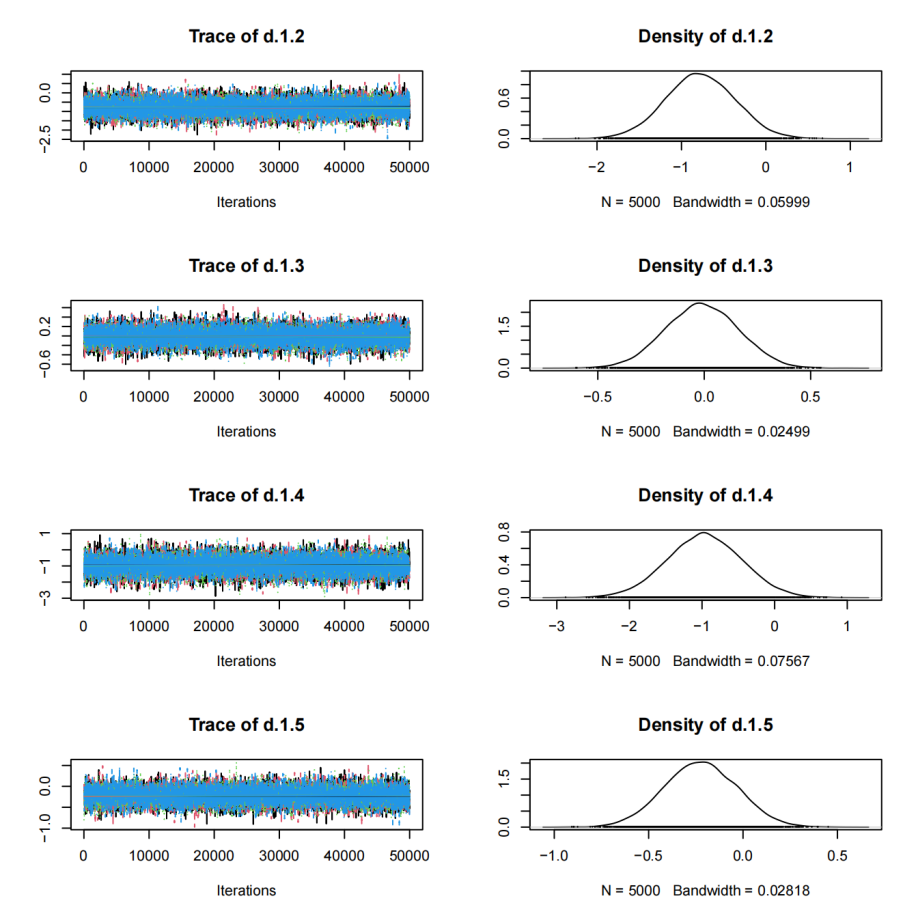


Figure S39.MCMC trace and posterior density plots for PFS in NBNC advanced hepatocellular carcinoma: immunotherapy vs tyrosine kinase inhibitors.


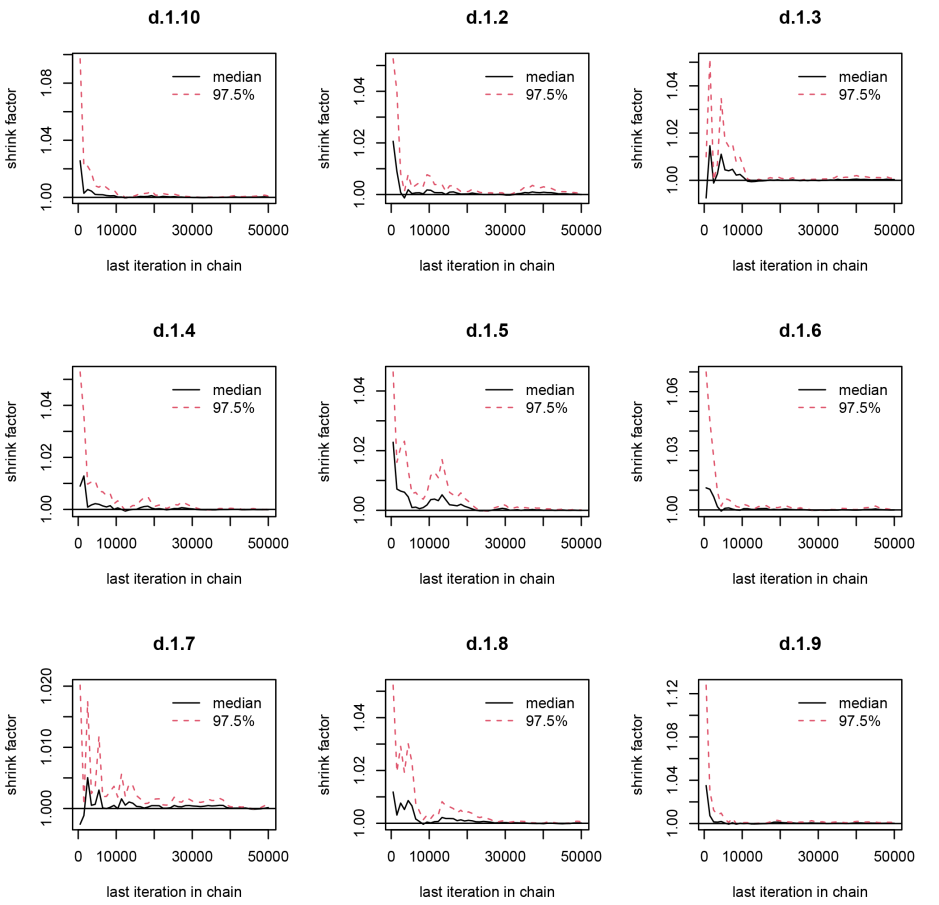


Figure S40.Convergence diagnostics for OS in advanced hepatocellular carcinoma: immunotherapy vs tyrosine kinase inhibitors.


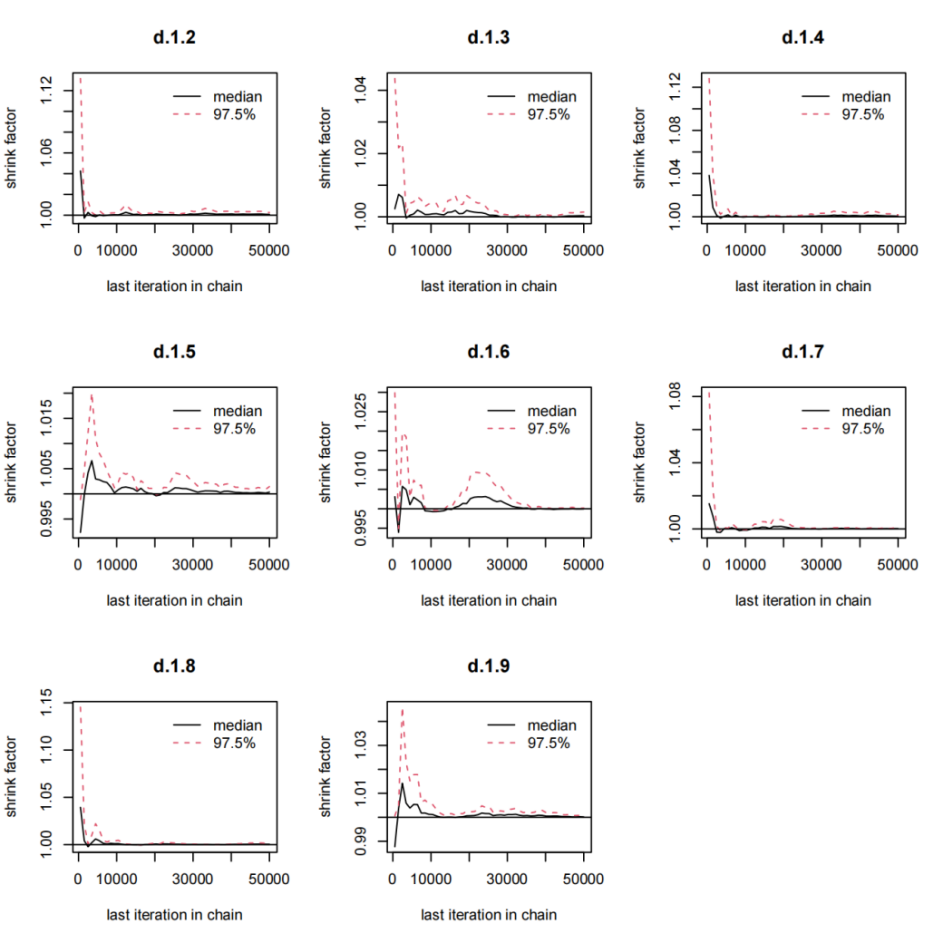


Figure S41.Convergence diagnostics for PFS in advanced hepatocellular carcinoma: immunotherapy vs tyrosine kinase inhibitors.


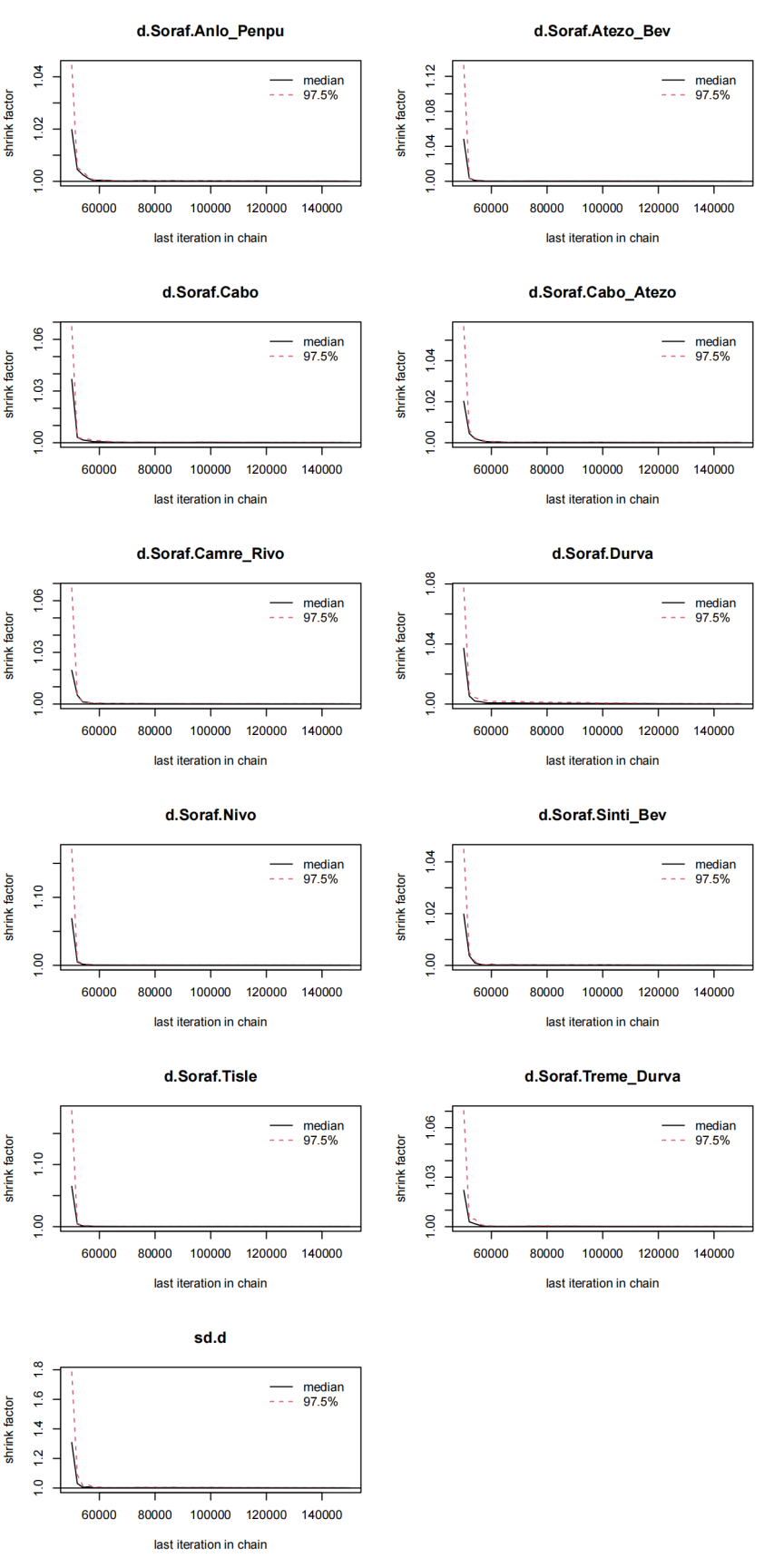


Figure S42.Convergence diagnostics for ORR in advanced hepatocellular carcinoma: immunotherapy vs tyrosine kinase inhibitors.


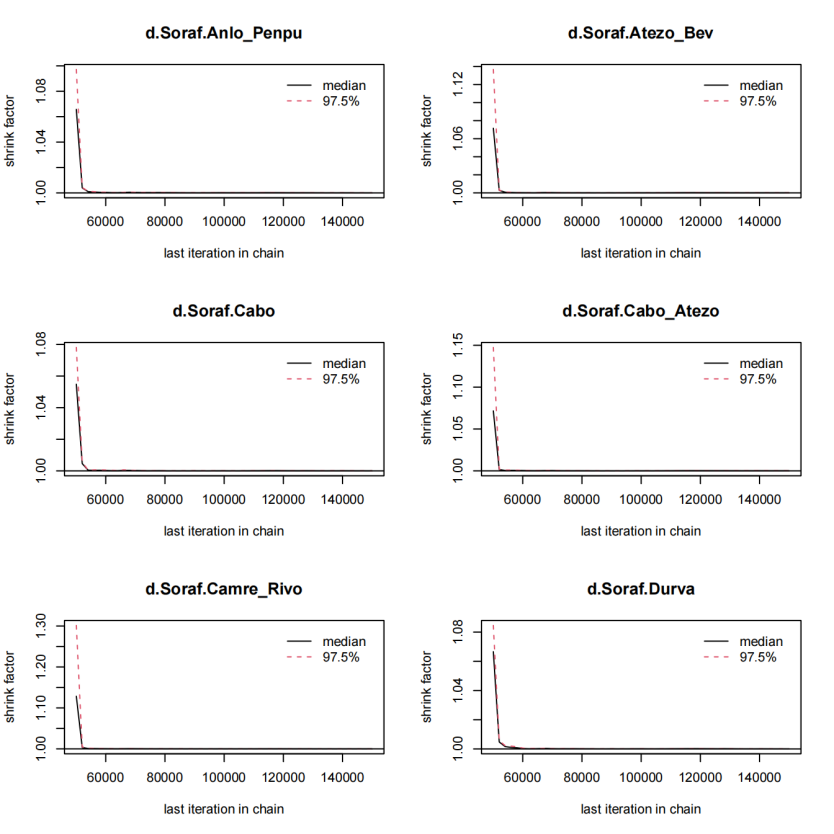


Figure S43.Convergence diagnostics for AEs≥3 in advanced hepatocellular carcinoma: immunotherapy vs tyrosine kinase inhibitors.


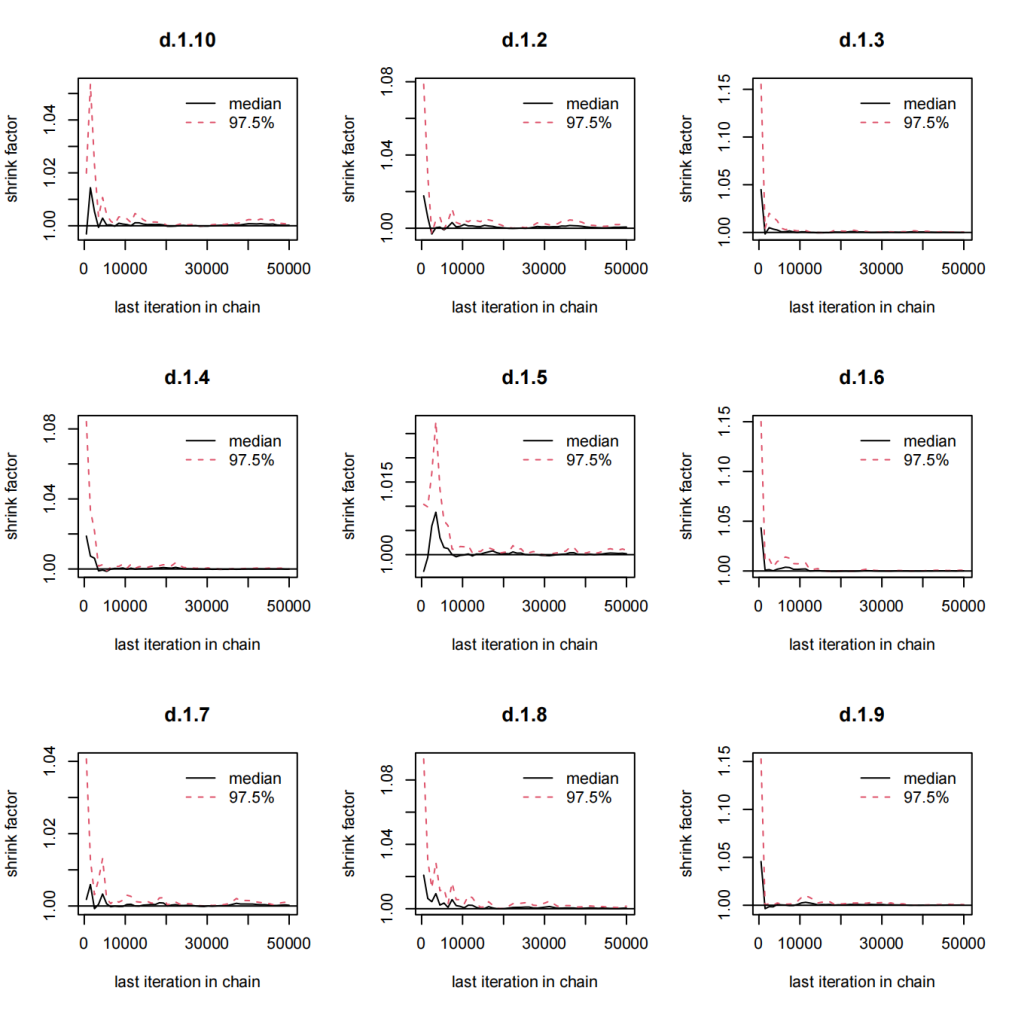


Figure S44.Convergence diagnostics for OS in HBV-positive advanced hepatocellular carcinoma: immunotherapy vs tyrosine kinase inhibitors.


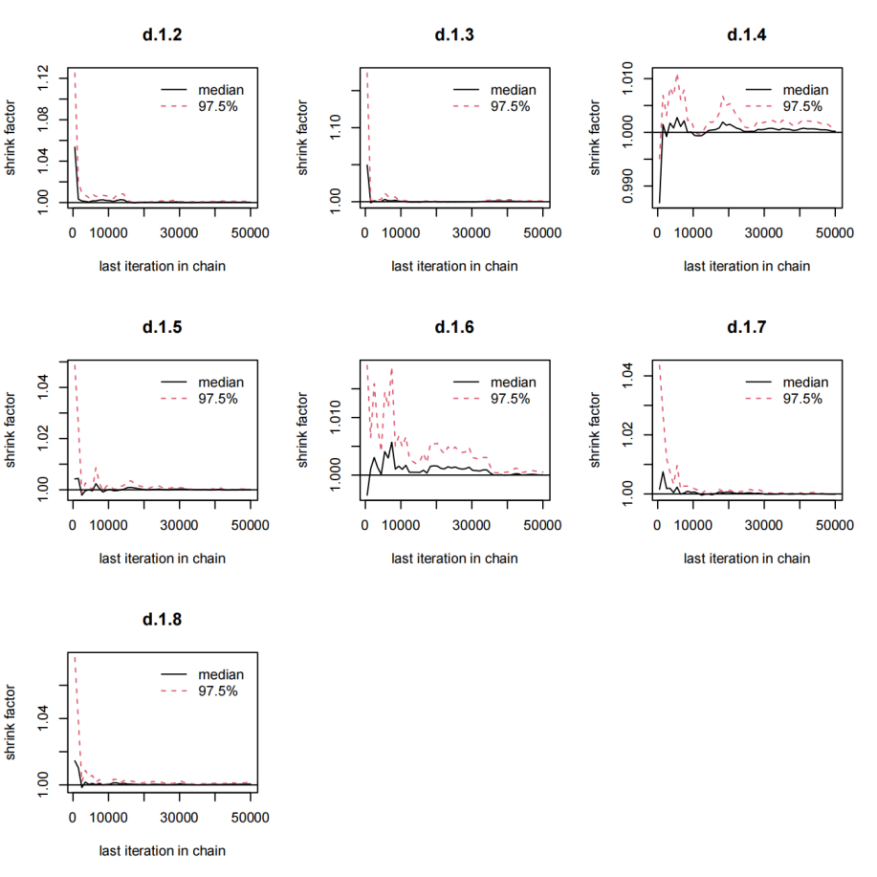


Figure S45.Convergence diagnostics for OS in HCV-positive advanced hepatocellular carcinoma: immunotherapy vs tyrosine kinase inhibitors.


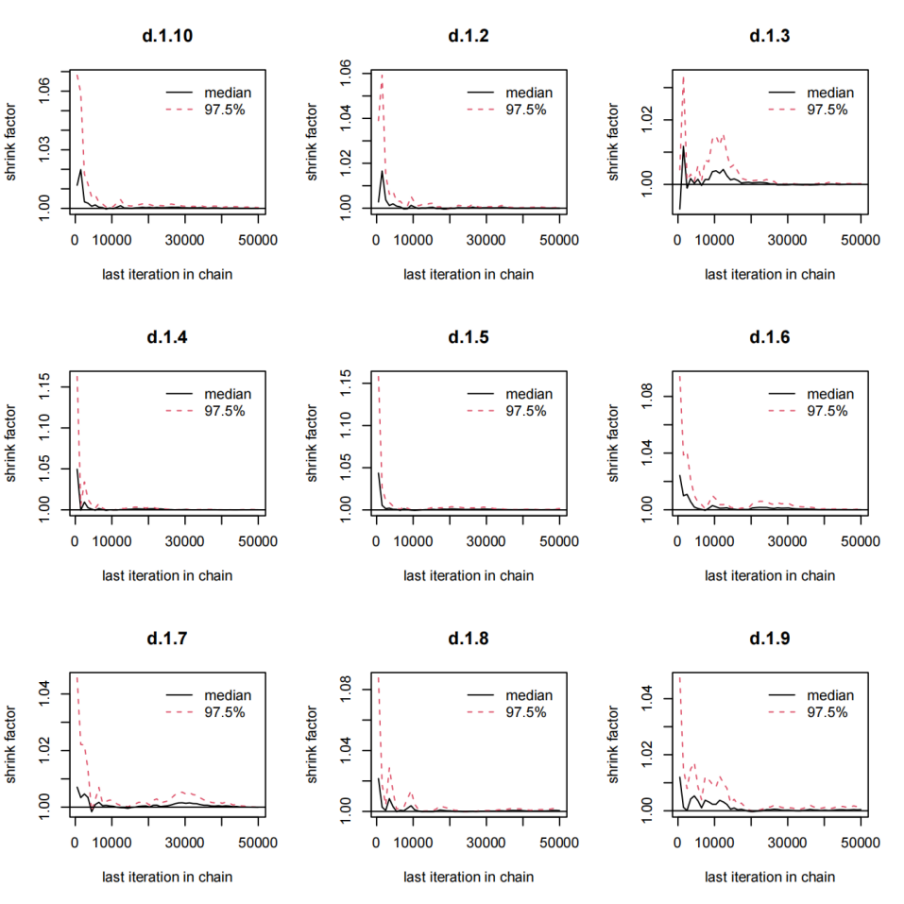


Figure S46.Convergence diagnostics for OS in NBNC advanced hepatocellular carcinoma: immunotherapy vs tyrosine kinase inhibitors.


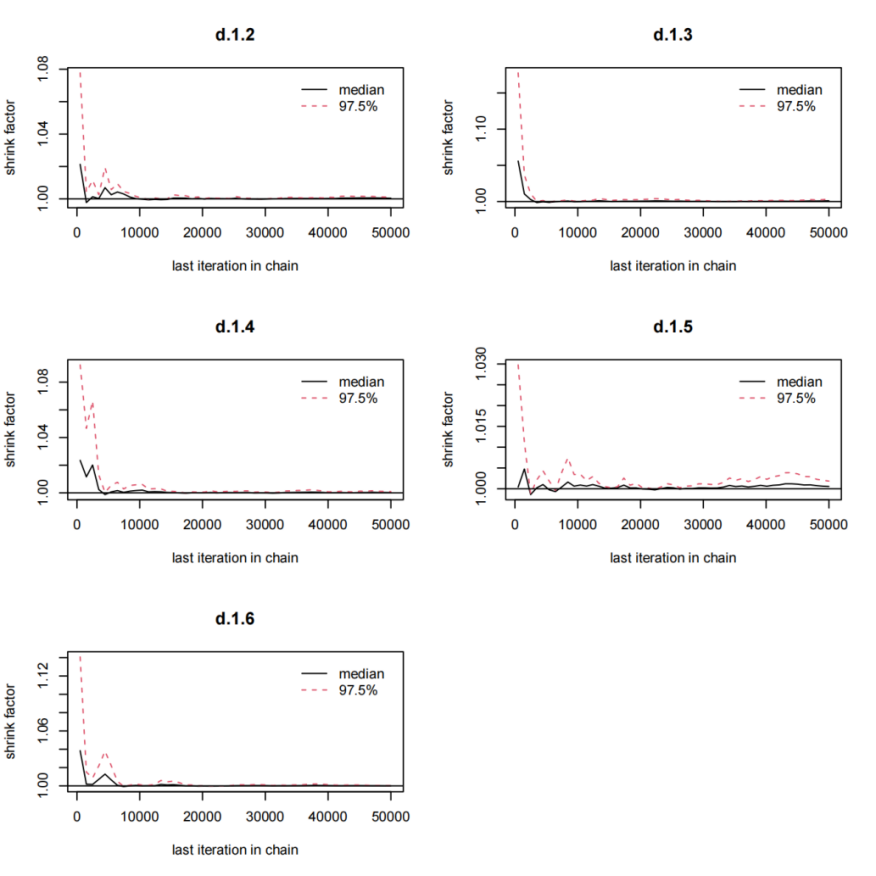


Figure S47.Convergence diagnostics for PFS in HBV-positive advanced hepatocellular carcinoma: immunotherapy vs tyrosine kinase inhibitors.


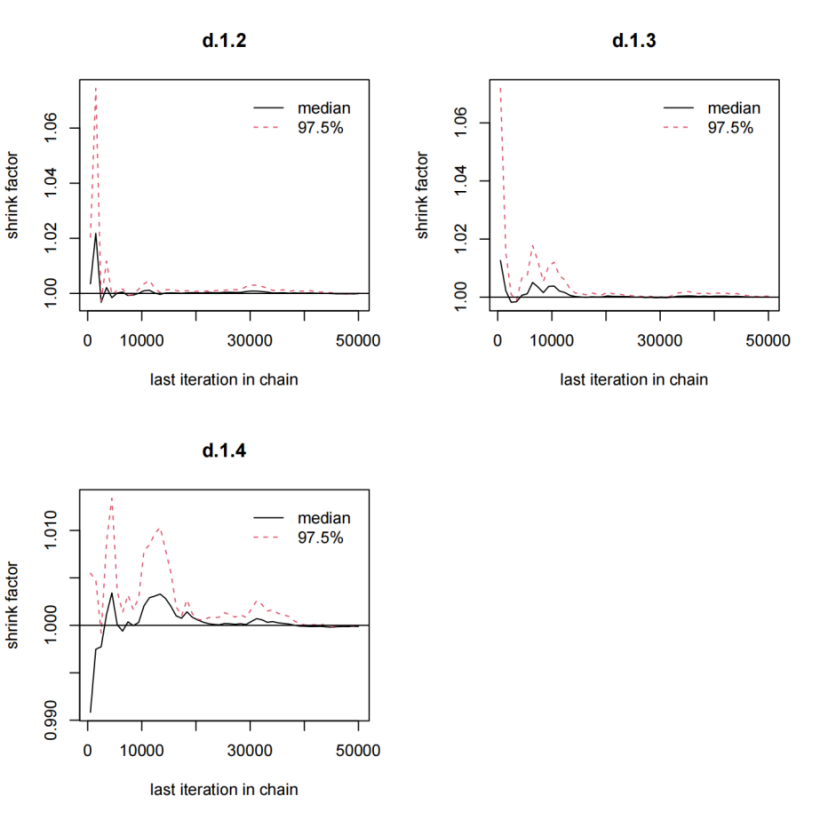


Figure S48.Convergence diagnostics for PFS in HCV-positive advanced hepatocellular carcinoma: immunotherapy vs tyrosine kinase inhibitors.


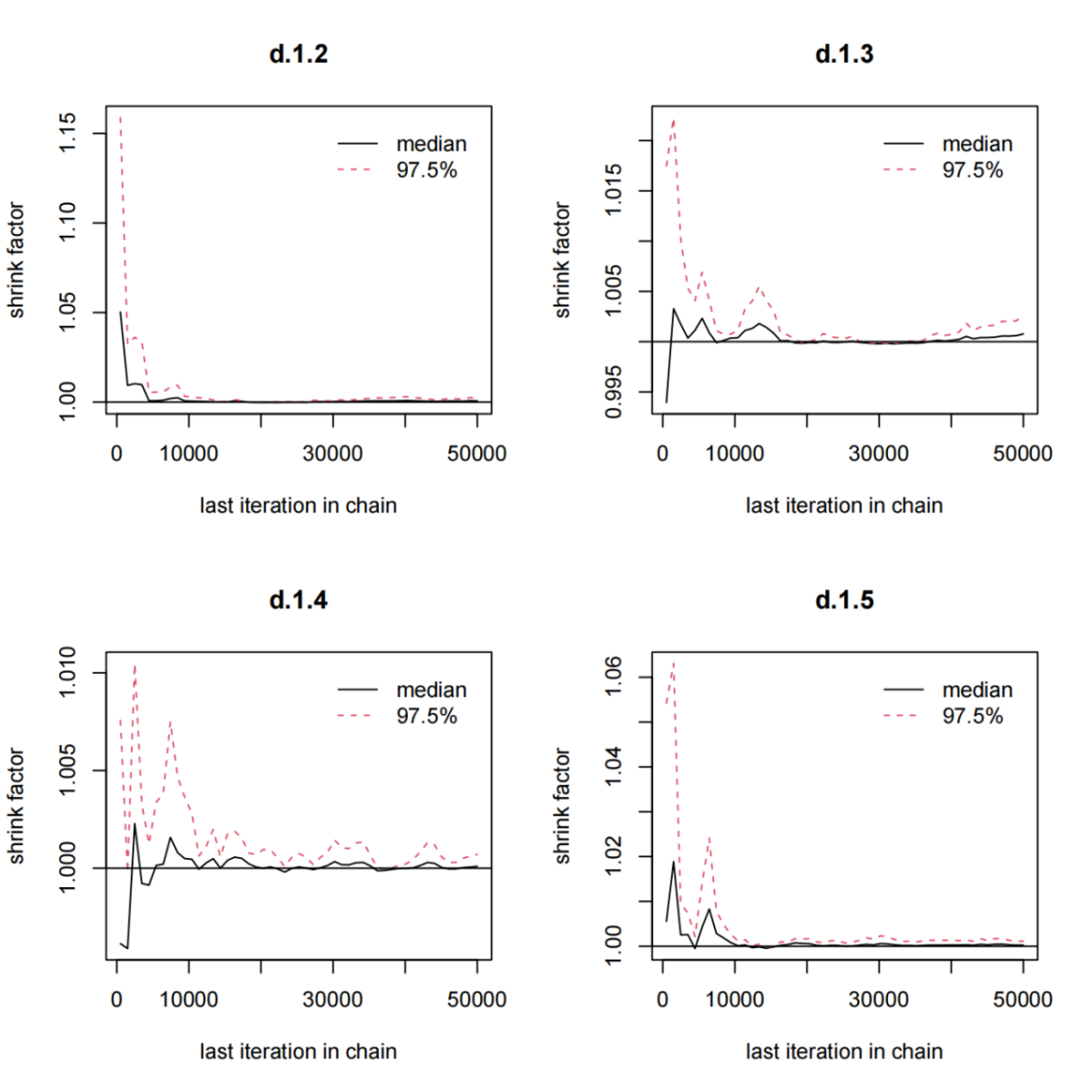


Figure S49.Convergence diagnostics for PFS in NBNC advanced hepatocellular carcinoma: immunotherapy vs tyrosine kinase inhibitors.
